# Immunological Feature and Transcriptional Signaling of Ly6C Monocyte Subsets From Transcriptome Analysis in Control and Hyperhomocysteinemic Mice

**DOI:** 10.3389/fimmu.2021.632333

**Published:** 2021-02-25

**Authors:** Pingping Yang, Lu Liu, Lizhe Sun, Pu Fang, Nathaniel Snyder, Jason Saredy, Yong Ji, Wen Shen, Xuebin Qin, Qinghua Wu, Xiaofeng Yang, Hong Wang

**Affiliations:** ^1^ Department of Cardiovascular Medicine, The Second Affiliated Hospital of Nanchang University, Nanchang, China; ^2^ Department of Pharmacology, Center for Metabolic Disease Research, Lewis Kats School of Medicine, Temple University, Philadelphia, PA, United States; ^3^ Department of Cardiovascular Medicine, The First Affiliated Hospital of Xi’an Jiaotong University, Xi’an, China; ^4^ Key Laboratory of Cardiovascular Disease and Molecular Intervention, Nanjing Medical University, Nanjing, China; ^5^ Tulane National Primate Research Center, School of Medicine, Tulane University, Covington, LA, United States

**Keywords:** lymphocyte antigen 6 complex, locus C (Ly6C) monocyte subset, hyperhomocysteinemia, transcription factor, immunological gene, immune checkpoint

## Abstract

**Background:**

Murine monocytes (MC) are classified into Ly6C^high^ and Ly6C^low^ MC. Ly6C^high^ MC is the pro-inflammatory subset and the counterpart of human CD14^++^CD16^+^ intermediate MC which contributes to systemic and tissue inflammation in various metabolic disorders, including hyperhomocysteinemia (HHcy). This study aims to explore molecule signaling mediating MC subset differentiation in HHcy and control mice.

**Methods:**

RNA-seq was performed in blood Ly6C^high^ and Ly6C^low^ MC sorted by flow cytometry from control and HHcy cystathionine β-synthase gene-deficient (*Cbs* ^-/-^) mice. Transcriptome data were analyzed by comparing Ly6C^high^ vs. Ly6C^low^ in control mice, Ly6C^high^ vs. Ly6C^low^ in *Cbs^-/-^* mice, *Cbs^-/-^* Ly6C^high^ vs. control Ly6C^high^ MC and *Cbs^-/-^* Ly6C^low^ vs. control Ly6C^low^ MC by using intensive bioinformatic strategies. Significantly differentially expressed (SDE) immunological genes and transcription factor (TF) were selected for functional pathways and transcriptional signaling identification.

**Results:**

A total of 7,928 SDE genes and 46 canonical pathways derived from it were identified. Ly6C^high^ MC exhibited activated neutrophil degranulation, lysosome, cytokine production/receptor interaction and myeloid cell activation pathways, and Ly6C^low^ MC presented features of lymphocyte immunity pathways in both mice. Twenty-four potential transcriptional regulatory pathways were identified based on SDE TFs matched with their corresponding SDE immunological genes. Ly6C^high^ MC presented downregulated co-stimulatory receptors (CD2, GITR, and TIM1) which direct immune cell proliferation, and upregulated co-stimulatory ligands (LIGHT and SEMA4A) which trigger antigen priming and differentiation. Ly6C^high^ MC expressed higher levels of macrophage (MΦ) markers, whereas, Ly6C^low^ MC highly expressed lymphocyte markers in both mice. HHcy in *Cbs* ^-/-^ mice reinforced inflammatory features in Ly6C^high^ MC by upregulating inflammatory TFs (*Ets1* and *Tbx21*) and strengthened lymphocytes functional adaptation in Ly6C^low^ MC by increased expression of CD3, DR3, ICOS, and *Fos*. Finally, we established 3 groups of transcriptional models to describe Ly6C^high^ to Ly6C^low^ MC subset differentiation, immune checkpoint regulation, Ly6C^high^ MC to MΦ subset differentiation and Ly6C^low^ MC to lymphocyte functional adaptation.

**Conclusions:**

Ly6C^high^ MC displayed enriched inflammatory pathways and favored to be differentiated into MΦ. Ly6C^low^ MC manifested activated T-cell signaling pathways and potentially can adapt the function of lymphocytes. HHcy reinforced inflammatory feature in Ly6C^high^ MC and strengthened lymphocytes functional adaptation in Ly6C^low^ MC.

## Introduction

Monocytes (MC) are bone marrow (BM) derived mononuclear phagocytes that play an important role in innate immune response and are the major immune cell population in chronic tissue inflammatory (1, 2). MC can be classified into inflammatory or anti-inflammatory subsets (1). Human MC were initially divided into three subsets based on the cell surface expression of CD14 and CD16, and recently classified based on CD40 expression (2–5). Murine MC are divided into three subsets based on surface expression of lymphocyte antigen 6 complex, locus C (Ly6C) (3, 4). Murine Ly6C^high^ and Ly6C^middle^ MC subsets perform pro-inflammatory functions, which are considered the counterpart of human CD14^++^CD16^+^ intermediate MC or CD14^+^CD40^+^ inflammatory MC (4, 5). Murine Ly6C^low^ MC perform patrolling and anti-inflammatory function, similar to human CD14^+^ CD16^++^ non-classical, CD14^++^CD16^-^ classical MC, and CD14^+^CD40^-^ anti-inflammatory MC (4, 5). Various studies support the notion that Ly6C^high^ MC can be differentiated into Ly6C^low^ MC (6–8). However, the selective impairment of Ly6C^high^ MC in *Irf8* ^−/−^ mutant murine demonstrated an independent developmental pathway for Ly6C^low^ MC (9). It was reported that certain transcription factors (TF) (e.g. NR4A1, CEBPβ) controlled Ly6C^low^ MC differentiation in the BM (10, 11). TF CEBPβ was shown to regulate Ly6C^low^ MC differentiation by controlling orphan nuclear receptor NR4A1 expression (10, 11). CEBPβ-deficient mice lacked Ly6C^low^ MC (11). However, the molecular mechanism underlying MC subset differentiation and transcriptional regulation remain to be elucidated.

Ly6C is a member of the lymphocyte antigen-6 (Ly6)/urokinase-type plasminogen activator receptor superfamily and a glycosylphosphatidylinositol-anchored glycoprotein with undefined function (12). Ly6C is first identified as an antigen shared by ∼50% of BM cells and expressed on dendritic cells (DC), macrophages (MФ), neutrophils, natural killer (NK) cells, CD4^+^ and CD8^+^ T-cell (13). It was generally accepted that tissue-specific MΦ were first derived during embryogenesis, and then mainly maintained their populations by self-renewal (14–16). Ly6C^high^ MC displays developmental plasticity and are recruited to tissues to complement MФ and DC on demand (3, 4, 17). After entering tissues, Ly6C^high^ MC can be differentiated into MФ, DC or tissue-specific MФ, including bone osteoclast (18), liver Kupffer cells (19), skin Langerhans cells (20) and kidney and intestinal MΦ (21–23), which can also self-renewal (24). Ly6C^high^ MC released proinflammatory cytokines, such as IL (interleukin)-1, IL-18, IL-15, and MCP (MC chemoattractant protein)-1 to contribute to systemic/tissue inflammation and T-cell activation (25). The molecular mechanism underlying MC plasticity and subset differentiation remain unclear.

To explore the immunological feature and transcriptional regulatory mechanism in MC subsets, we analyzed the expression pattern of four sets of immunological genes (secretome, cytokine, surface marker and immune checkpoint). Secretome is a new term to describe proteins secreted to the extracellular space mediating cell-cell interactions (17). Cytokines are small soluble signaling proteins secreted by cells, which determine immune response (26). Most cytokines have defined functions to regulate immune responses including proliferation, trafficking, and differentiation by binding to corresponding receptors (26). Cell surface markers, such as cluster of differentiation (CD) molecules, regulate adhesion, immune recognition and cell-cell interaction (27, 28). Lineage-specific cell-surface markers are characteristic molecules used to define specific lineage and stage in the differentiation process (29, 30). Recent progress in a single-cell RNA sequencing (scRNA-seq) study proposed a group of new signature genes to define novel immune cell populations (31). Immune checkpoints are cell surface molecular pairs (receptors and their ligands) classified into co-stimulatory and co-inhibitory immune checkpoint (25, 32). Co-stimulatory signals activate T-cell or antigen-presenting cell to regulate differentiation, proliferation, cytokines secretion, and receptor expression (33). Co-inhibitory signals are negative regulators of immune response to avoid immune injury or turn down the immune system (25, 34).

We previously demonstrated that hyperhomocysteinemia (HHcy), an independent risk factor for cardiovascular, diabetic and Alzheimer’s disease, induced Ly6C^high^ inflammatory MC subsets differentiation, which contributed to tissue inflammatory and accelerated arteriosclerosis and chronic kidney disease (5, 35–39). The effect of HHcy on MC subset differentiation in patient would be an interesting topic for future clinical research. Discover of regulatory mechanisms mediating HHcy-induced MC subset differentiation may lead to the discovery of novel therapeutic target. This study aims to systemically examine mRNA expression profiles of key immunological genes in Ly6C^high^ and Ly6C^low^ MC subsets by intensive bioinformatic analysis and to develop models of molecule pathways and transcriptional regulatory signaling for subset differentiation.

## Research Design and Methods

**Figure 1:**
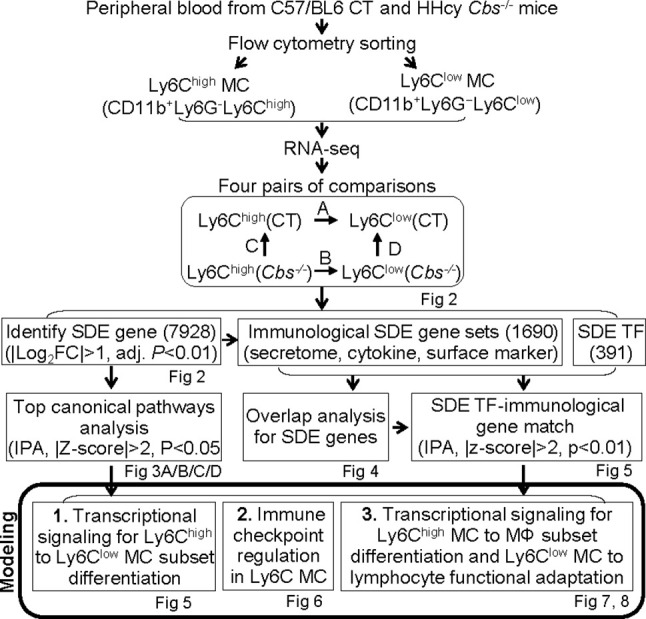
Overall strategy of the identification of Ly6C MC regulatory genes and molecule mechanism for Ly6C monocyte subset differentiation in control and *Cbs* ^-/-^ mice. RNA-seq were performed in Ly6C^high^ (CD11b^+^Ly6G^−^Ly6C^high^) and Ly6C^low^ (CD11b^+^Ly6G^−^Ly6C^low^) MC isolated by flow cytometry sorting from peripheral blood of C57/BL6 control and *Cbs* ^-/-^ mice. Transcriptome data were analyzed by performing four pairs of comparisons; **(A)** Ly6C^high^ vs. Ly6C^low^ (CT), **(B)** Ly6C^high^ vs. Ly6C^low^ (*Cbs^-/-^*), **(C)** *Cbs^-/-^* vs. CT (Ly6C^high^), **(D)** *Cbs^-/-^ vs.* CT (Ly6C^low^). We identified 7928 SDE genes using the Bioconductor suite of packages in RStudio software with the criteria of |Log_2_FC| more than 1 (2-FC) and adjusted P value less than 0.01. Top ingenuity pathways were identified by top-down analysis using IPA with |Z-score|>2, P value<0.05. Immunological SDE gene sets, including secretome, cytokine and surface marker were overlapped analysis and matched with corresponding upstream SDE TF by IPA upstream analysis. Three molecular signaling model system were developed, **1)** Transcriptional regulation for Ly6C^high^ to Ly6C^low^ MC subset differentiation, **2)** Immune checkpoint regulation in Ly6C MC. **3)** Transcriptional signaling for Ly6C^high^ MC to MΦ subset differentiation and Ly6C^low^ MC to lymphocyte functional adaptation, CT, control, HHcy, Hyperhomocysteinemia; RNA-seq, RNA-sequencing; MC, monocyte; *Cbs*, Cystathionine β-synthase; SDE, significant differentially expressed; IPA, Ingenuity Pathway Analysis, TF, transcription factor, MΦ, macrophage.

We summarized the overall study approaches and strategies in **Figure 1**.

### HHcy Mice

The Tg-h*CBS Cbs* ^-^ *^/^* ^-^ mice were created as described previously (35, 40). The human CBS transgene (*Tg-hCBS*) was introduced in *Cbs* ^-^ *^/^* ^-^ mice to rescue neonatal lethality and is under the control of a Zn-inducible metallothionein promoter (40). Mice were all born to mothers drinking ZnCl_2_ water (25 mM) to induce transgene expression (35, 40). ZnCl_2_ was withdrawn after weaning at 1 month of age to allow the development of HHcy. Animals were fed standard rodent chow diet and sacrificed at 22 weeks for blood collection after euthanization. Mouse protocols were approved by the Temple University Institutional Animal Care and Use Committee.

### Hcy Measurement

Mouse blood was collected into 1 mM ethylenediaminetetraacetic acid (EDTA)-coated tubes. A total of 50 μl of plasma was batched and stored at -20 °C for Hcy measurement as previously described (41). In brief, total Hcy levels were tested by liquid chromatography-electrospray ionization-tandem mass spectrometry.

### Flow Cytometry and Cell Sorting

Mouse peripheral blood was collected into 1 ml phosphate‐buffered saline (PBS) containing 5 μM EDTA in fluorescence-activated cell sorting (FACS) tube. White blood cells (WBC) were isolated by using (Ammonium-Chloride-Potassium) ACK lysing buffer (NH4Cl 0.15 M, KHCO3 10.0 mM, Na2 EDTA 0.1 mM) to lyse red blood cells. WBC from 11 mice were pooled and stained with antibodies against CD11b-Brilliant Violet 421 (myeloid cell marker, 0.25 μg/100 μl, clone M1/70), Ly6G-acticated protein C(APC)/Cy7 (granulocyte marker; 0.25 μg/100 μl, clone 1A8), Ly6C-APC (inflammatory MC marker, 0.25 μg/100 μl, clone HK1.4, BD Pharmingen, San Diego, CA), and subjected for flow cytometry cell sorting. CD11b^+^ Ly6G^-^ Ly6C^high^ and CD11b^+^ Ly6G^-^ Ly6C^low^ MC were sorted on a BD FACSAria III cell sorter. Fluorescent activated cells were analyzed offline with FlowJo software (Tree Star Inc, Ashland, OR, version 10) and compiled using Prism software (GraphPad, version 6). All populations were routinely backgated to verify gating and purity.

### RNA Sequencing in Monocyte Subsets

Flow cytometry sorted CD11b^+^Ly6G^-^Ly6C^high^ and CD11b^+^Ly6G^-^Ly6C^low^ cells from control and *Cbs* ^-/-^ WBC (200000/MC subset) were collected in 700 μl QIAzol Lysis Reagent (Qiagen, Germantown, MD) for total RNA extraction. Samples were quality checked on an Agilent Bioanalyzer 2100 using pico RNA chip for RNA integrity number. Total RNA (50–100 ng/sample) were used for cDNA library construction after ribosomal cDNA depletion using Takara pico-input kit. Pooled samples were run for sequencing analysis in duplication on Illumina NextSeq 500 (CT) and Illumina Hiseq 4000 sequencer (HHcy).

RNA-seq data from this study are available from the corresponding author upon reasonable request in reference to recent similar publication (42). Details for major RNA-seq data resources can be found in **Supplementary Material**.

### RNA Sequencing Data Processing

The raw reads were mapped to the mouse reference transcriptome (mouse cDNA FASTA from ensembl, website http://uswest.ensembl.org/info/data/ftp/index.html) using Kallisto, version 0.45. Genes with less than 1 count per million reads in at least 2 or more samples were filtered out. This reduced the number of genes to 16,476 normalized genes. The raw RNA-seq data was analyzed using the statistical computing environment R, the Bioconductor suite of packages for R and RStudio (tidyverse, reshape2, tximport, biomaRt, RColorBrewer, genefilter, edgeR, matrixStats, hrbrthemes, gplots, limma, DT, gt, plotly, beepr, skimr, cowplot, data.table, sva).

### Principle Component Analysis

PCA was performed to examine the variance of RNA-seq data. RNA-seq data from control and *Cbs* ^-/-^ mice were produced at different times and processed to remove batch effects and other unwanted noise using ComBat approach (43, 44). The first 2 principal components (PC1 and PC2) were used to depict the similarity between samples.

### Identification of Significantly Differentially Expressed Gene

SDE genes were identified using the Bioconductor suite of Limma packages in RStudio software with the criteria of |Log_2_ fold change (FC)| more than 1 (FC>2) and adjusted *P*-value less than 0.01. We identified genes differentially expressed (|FC|>2, *P*<0.01) in Ly6C^high^ and Ly6C^low^ MC by performing four pairs of comparisons: **A**. Ly6C^high^ vs. Ly6C^low^ (control), **B**. Ly6C^high^ vs.Ly6C^low^ (*Cbs^-/-^*), **C**. *Cbs^-/-^* vs. control (Ly6C^high^), **D**. *Cbs^-/-^ vs.* control (Ly6C^low^). We identified 2641 secretome, 1176 cytokines and 377 surface markers collected in Protein Atlas (https://www.proteinatlas.org) (45) and 49 immune checkpoint gene based on the current literature (25), and newly suggested leukocyte signature genes from recent scRNA-seq study (46, 47). SDE immunological genes were overlapped with SDE gene in immunological gene.

### Volcano Plot and Heatmap

Volcano plot was used as a scatterplot to show the differential expression of genes that shows statistical significance (-Log_10_adjust *P*-value) versus magnitude of change (Log_2_FC). Heatmap was generated in RStudio using the pheatmap package to present the expression levels of SDE genes. The color density in the heatmap indicates the average expression levels of a given gene normalized by z-score.

### Identification of Functional Pathways

We used Ingenuity Pathway Analysis (IPA) version 7.1 (IPA, Ingenuity Systems, https://www.ingenuity.com) to identify functional pathways. SDE genes were identified and uploaded into IPA for analysis. The general canonical functional pathways were established for SDE genes identified in above mentioned four comparison groups, as we have previously reported (48, 49).

### Overlap Analysis of SDE Genes

SDE genes and functional pathways identified from above mentioned four comparisons were subjected for overlapping analysis (http://bioinformatics.psb.ugent.be/webtools/Venn/). Venn diagrams were displayed to present SDE genes and pathways overlaps between comparisons. Further, functional pathways were also established for three sets of immunological SDE genes (secretome, cytokines and surface markers) and SDE TF. Functional pathways in Venn diagram were developed by using metascape website software (https://metascape.org/) for SDE gene set (>20 SDE genes).

### Identification of Transcriptional Signaling

We identified SDE TFs and matched with their corresponding SDE immunological genes by referencing TF- matched gene sets using IPA upstream analysis. The significate matches were recognized as potential transcriptional signaling (TF/targeted molecule axis) based on *p*‐values < 0.01, |*z*‐scores|>2, calculated by using Fisher’s Exact Test.

## Results

### Identification of 7928 Significantly Differentially Expressed Genes Through Four Comparisons in Sorted Blood Ly6C^high^ and Ly6C^low^ Monocytes From Control and *Cbs^-/-^* Mice

**Figure 2:**
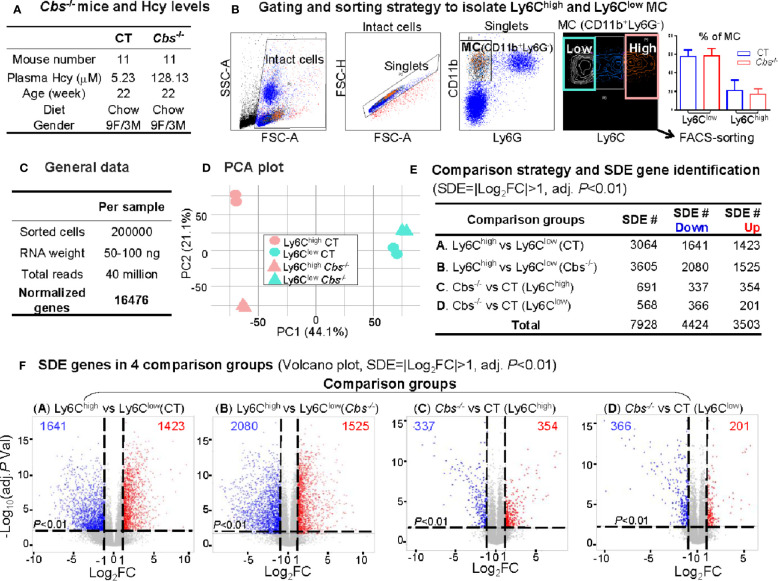
RNA-Seq analysis and SDE gene identification from blood Ly6C^high^ and Ly6C^low^ MC of C57/BL6 control and *Cbs* ^-/-^ mice. **(A)** *Cbs* ^-/-^ mice and Hcy levels. Eleven mice were used in each group. Severe HHcy were determined in *Cbs* ^-/-^ mice (plasma Hcy 128.13 μmol/L). **(B)** Gating and sorting strategy to isolate Ly6C^high^ and Ly6C^low^ MC. Mouse white blood cell were prepared from peripheral blood and stained with antibody against CD11b, Ly6G and Ly6C and subjected for flow cytometry cell sorting. Intact cells (72.8%) were recognized based on higher FSC-A (larger size). Singlets (71.0%) were identified by using FSC-H versus FSC-A appeared on a diagonal. CD11b^+^Ly6G^-^ cells (11.0%) were selected as MC. MC subsets (CD11b^+^Ly6G^-^Ly6C^high^, and CD11b^+^Ly6G^-^Ly6C^low^) were sorted based on Ly6C levels. The quantification of MC was used flow cytometry analysis for Ly6C^high^ and Ly6C^low^ MC in CT and *Cbs^-/-^*. **(C)** General data. 100 ng mRNA were obtained from 100,000 sorted cells and achieved around 30 million reads and 16,487 normalized genes per sample by mRNA-Seq analysis. **(D)** PCA plot. PCA analysis incorporated 8 samples from 4 groups of MC subsets {Ly6C^high^ (CT), Ly6C^low^ (CT), Ly6C^high^ (*Cbs^-/-^*) and Ly6C^low^ (*Cbs^-/-^*), n=2} using the R software package Seurat. PC1 versus PC2 demonstrates the close transcriptional proximity. PC1, PC2 and PC3 variance is 44.1%, 21.1% and 12.9%. PC1 (44.1%) means that the difference on the x-axis can explain 44.1% of the overall result. **(E)** Comparison strategy and SDE gene identification. We performed four group comparison **(A–D)** and identified down-regulated and up-regulated SDE genes using the criteria of |Log_2_FC| more than 1 (2-FC) and adjusted P value less than 0.01. **(F)** SDE genes in four comparison groups. Volcano plot of all genes demonstrates the expression pattern of SDE genes in four comparison groups. Down-regulated SDE genes are highlighted in green and up-regulated in red (|Log_2_FC|>1, adj. *P*<0.01), with Log_2_FC as x-axis and −Log_10_(adjust *P*-value) as y-axis. MC, monocyte; CT, control; *Cbs*, cystathionine β-synthase; HHcy, Hyperhomocysteinemia; Hcy, homocysteine; FACS, fluorescent-activated cell sorting; PCA, principal component analysis; PC, principal component; SDE, significantly differentially expressed; FC, fold change.

We obtained 40 million reads and 16476 normalized genes from RNA-Seq analysis of 200000 sorted Ly6C^high^ (CD11b^+^Ly6G^-^Ly6C^high^) and Ly6C^low^ (CD11b^+^Ly6G^-^Ly6C^low^) MC from control C57/BL6 mice and HHcy *Cbs* ^-/-^ mice (plasma Hcy 5.23 μM and 128.13 μM) (**Figures 2A–C**). PCA presented a clear separation between Ly6C^high^ and Ly6C^low^ in both control and *Cbs^-/-^* samples (**Figure 2D**). There was also a good separation in Ly6C^high^ between control and *Cbs^-/-^* mice which was absent in Ly6C^low^. The PC1 axis showed the largest variations and explained 44.1% of the variances between Ly6C^high^ and Ly6C^low^ MC subsets. The PC2 axis explains 21.1% of the variance between *Cbs^-/-^* and control mice.

A total of 7,928 SDE genes with the criteria of |Log_2_FC| more than 1 (2-FC) and adjusted *P*-value less than 0.01 (**Figure 2E**) were identified through the previously mentioned comparison pairs (**Figure 2F**). We found 1,423 upregulated and 1,641 downregulated SDE genes in Ly6C^high^ MC compared with Ly6C^low^ MC in control mice (Comparison A). We identified 1,525 upregulated and 2,080 downregulated in Ly6C^high^ MC compared with Ly6C^low^ MC in *Cbs* ^-/-^ mice (Comparison B). When compared between the same subset among the two mouse groups, we discovered that HHcy in *Cbs* ^-/-^ mice upregulated 345 and downregulated 337 SDE genes in Ly6C^high^ MC (Comparison C), and upregulated 201 and downregulated 366 SDE genes in Ly6C^low^ MC (Comparison D).

### Ly6C^high^ Monocytes Enriched With Inflammatory Pathways and Ly6C^low^ Monocytes Presented Features of T Cell Activation Based on All Significantly Differentially Expressed Genes

**Figure 3:**
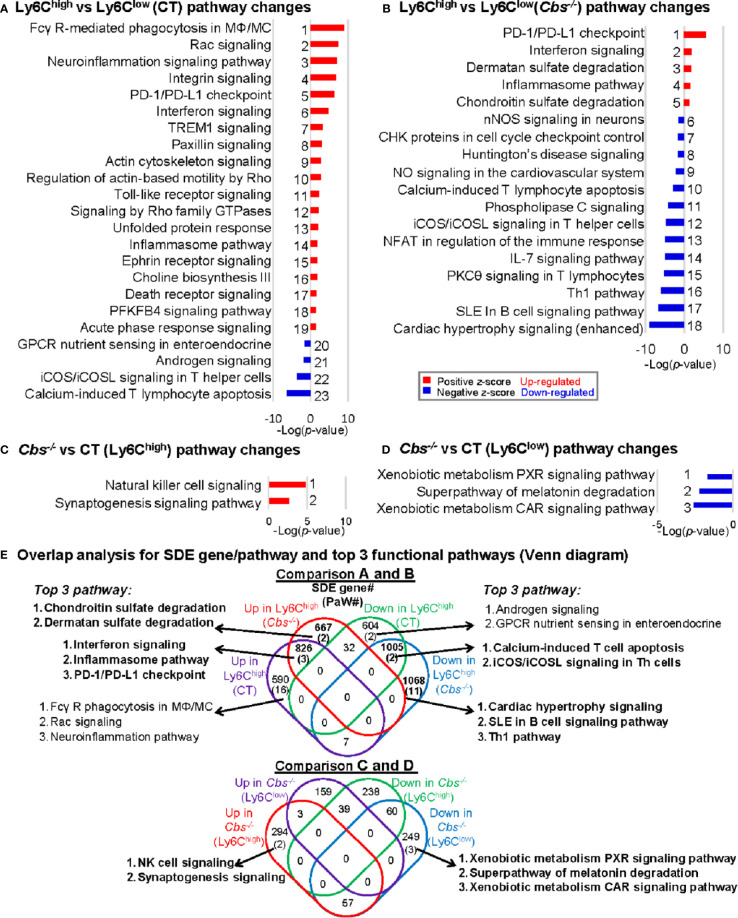
General canonical pathway analysis for SDE genes from four comparison groups. **(A)** Ly6C^high^ vs. Ly6C^low^ (CT) pathway changes; **(B)** Ly6C^high^ vs. Ly6C^low^ (*Cbs^-/-^*) pathway changes; **(C)** *Cbs^-/-^* vs. CT (Ly6C^high^) pathway changes; **(D)** *Cbs^-/-^* vs. CT (Ly6C^low^) pathway changes. Top canonical pathways were identified by top-down analysis using IPA software. Significant top IPA pathways are identified using the criteria of adjusted P value<0.05 and |Z-score|>2. Blue bar indicates a negative z-score and down-regulated pathway. Red bar indicates a positive z-score and up-regulated pathway. Representative top 40 up/down SDE genes involved in these top pathways are listed in **Supplementary Table 1**. **(E)** Overlap analysis for SDE genes in Ly6C MC subsets and top 3 functional pathways (Venn diagram). Venn diagram summarized the total SDE genes and their top 3 pathways in each SDE set in four pairs of comparisons. Numbers depict the amount of SDE genes. Numbers in the parentheses describes the number of pathways. MC, monocyte; MΦ, macrophage; TREM1, The triggering receptor expressed on myeloid cells 1; GPCRs, G-protein-coupled receptors; PFKFB4, 6-phosphofructo-2-kinase/fructose-2,6-biphosphatase 4; SLE, Systemic Lupus Erythematosus, Th1, T helper 1; PKCθ, Protein Kinase C Theta; IL-7, Interleukin 7; NFAT, Nuclear factor of activated T-cells; CHK, Csk-homologous kinase; nNOS, neuronal nitric oxide synthase; PXR, pregnane X receptor; CAR, constitutive androstane receptor.

We recognized 23, 18, 2, and 3 canonical pathways that were significantly enriched by top-down analysis using SDE gene identified from comparison groups A, B, C, and D, respectively, by using IPA software **(** **Figures 3A–D** **).** The details of the gene names, FC and molecular category of the top 40 up/down SDE genes involved in these pathways are listed in **Supplementary Table 1**.

Through overlap analysis (**Figure 3E** **)**, we discovered 21 activated pathways in Ly6C^high^ MC (16 in control mice, two in *Cbs* ^-/-^ mice, and three in both) in Comparisons A and B. These activated pathways were derived from 2084 SDE genes (590 in control, 667 in *Cbs* ^-/-^ and 826 in both). The top 3 pathways are depicted. Moreover, we found 15 suppressed pathways in Ly6C^high^ MC (2 in control, 11 in *Cbs* ^-/-^ and 2 in both). These suppressed pathways were derived from 2677 SDE genes (604 in control only, 1,068 in *Cbs* ^-/-^ only and 1,005 in both). From comparison C and D, we discovered two activated pathways and three suppressed pathways in Ly6C^high^ and Ly6C^low^ MC in *Cbs* ^-/-^ mice, respectively. The two activated pathways in *Cbs* ^-/-^ Ly6C^high^ MC were derived from 294 SDE genes. The three suppressed pathways in *Cbs* ^-/-^ Ly6C^low^ MC were derived from 249 SDE genes.

There were 3 activated pathways overlapped in Ly6C^high^ MC from both control and *Cbs* ^-/-^ mice. These include interferon, inflammasome and PD-1/PD-L1 checkpoint pathways. Two suppressed pathways, T-cell apoptosis and Th cell signaling, were overlapped in Ly6C^high^ from both control and *Cbs* ^-/-^ mice.

Specifically, sulfate degradation was activated, and Th1/B-cell pathway was suppressed only in Ly6C^high^ from *Cbs* ^-/-^ mice. Whereas, NK cell signaling were activated in Ly6C^high^ and a few metabolic pathways, including xenobiotic metabolism and melatonin degradation, were suppressed in Ly6C^low^ MC only in *Cbs* ^-/-^ mice as detailed in **Figures 3C–E**.

### Ly6C^high^ Monocytes Exhibited Activated Inflammatory and Lysosome Activation Pathways, Whereas, Ly6C^low^ Monocytes Presented Features of Lymphocyte Immunity Pathways Based on Significantly Differentially Expressed Immunological Signature Genes

**Figure 4:**
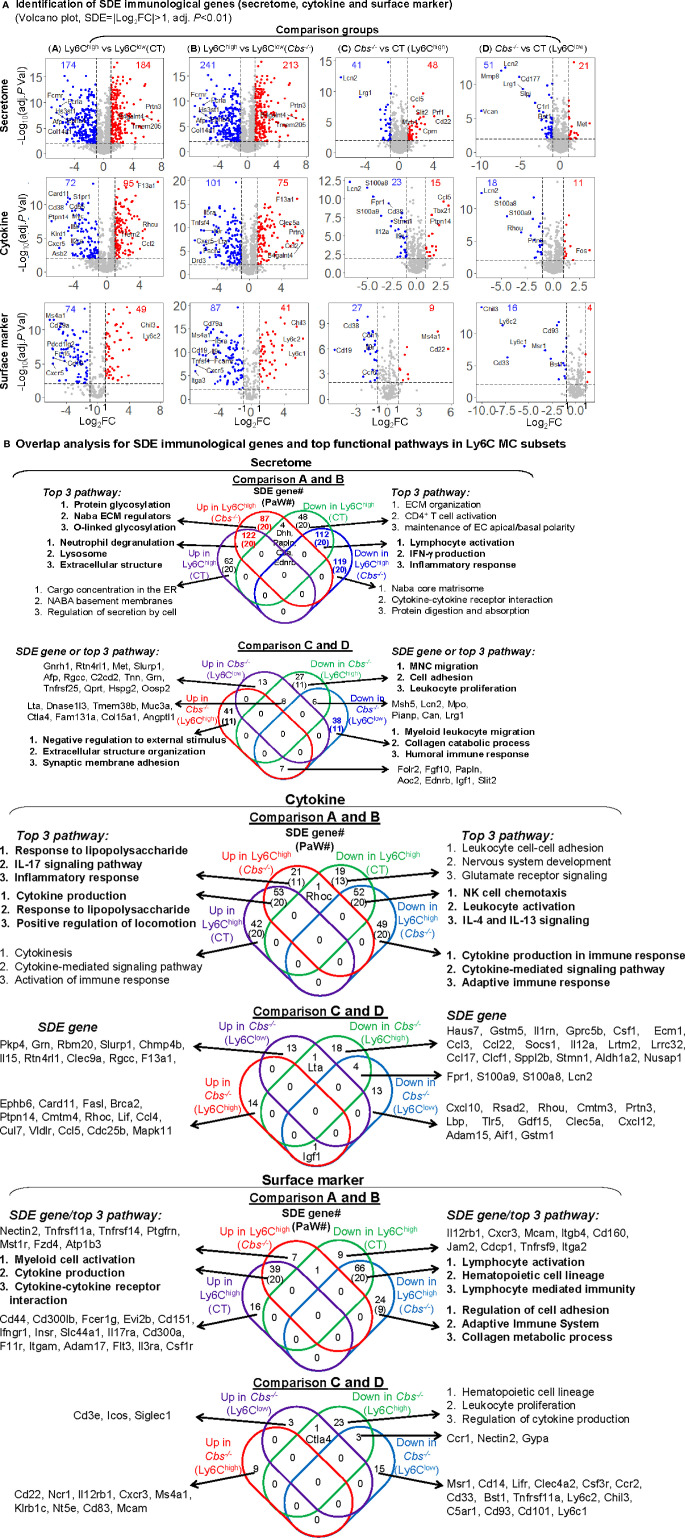
Immunological signature genes and top functional pathways in Ly6C MC subset from CT and *Cbs* ^-/-^ mice. **(A)** Identification of immunological SDE genes (secretome, cytokine and surface marker). Volcano plot of all genes demonstrates the expression pattern of SDE genes in four comparison groups. Down-regulated SDE genes are highlighted in green and up-regulated in red (|Log_2_FC|>1, adj. P<0.01), with Log_2_FC as x-axis and −Log_10_(adjust *P*-value) as y-axis. SDE secretome, cytokine and surface marker were identified using the immunological gene set established in our previous study (PMID: 32179051) from website (https://www.proteinatlas.org/). Top 25 up- and down-regulated SDE genes in all comparisons *via* IPA are listed in **Supplementary Table 2**. **(B)** Overlap analysis for SDE immunological genes in Ly6C MC subsets and top pathways. Venn diagram summarized the total SDE genes and their top three pathways in each SDE set from four pairs of comparisons. Functional pathways were developed by metascape software mainly using the GO database only in SDE set (>20 SDE genes). The top 3 functional pathways are presented. Numbers depict the amount of SDE genes. Numbers in the parentheses describes the number of pathways. A detailed list of SDE genes and pathway in each SDE set are presented in **Supplementary Table 3**. ECM, extracellular matrix; EC, extracellular; IFNγ, interferon gamma; MNC, mononuclear cell; NK, natural killer.

In comparison A, we identified 184-upregulated/174-downregulated secretome, 95-upregulated/72-downregulated cytokine, and 49-upregulated/74-downregulated surface marker SDE genes in Ly6C^high^ MC from control mice (**Figure 4A**). In comparison B, we found 213-upregulated/241-downregulated secretome, 75-upregulated/101-downregulated cytokine, 41-upregulated/87-downregulated surface marker SDE genes in Ly6C^high^ MC from *Cbs* ^-/-^ mice. When compared the same subset between the two mouse groups, we found that HHcy induced 48-upregulated/41-downregulated secretome, 15-upregulated/23-downregulated cytokine, and 8-upregulated/27-downregulated surface marker SDE genes in Ly6C^high^ MC, and 21-upregulated/51-downregulated secretome, 11-upregulated/18-downregulated cytokine, and 4-upregulated/16-downregulated surface marker SDE genes in Ly6C^low^ MC in *Cbs* ^-/-^ mice. The details and FC of the top 25 up/down immunological SDE genes were listed in **Supplementary Table 2**.

In SDE gene-derived pathway overlap analysis, presented in Venn diagram in **Figure 4B**, we found 20-activated/20-suppressed pathways from SDE secretome genes in Ly6C^high^ MC from both control and *Cbs* ^-/-^ mice (Comparisons A and B). The top pathways indicated the activation of lysosome and extracellular structure, and suppression of lymphocyte activation, IFN-γ production and inflammatory response in Ly6C^high^ MC. In addition, we identified secretome SDE gene-derived pathway specific for Ly6C^high^ for each mouse. For example, protein glycosylation and ECM regulation were activated in Ly6C^high^ only in *Cbs* ^-/-^ mice. Moreover, HHcy in *Cbs* ^-/-^ mice specifically activated extracellular structure organization and synaptic membrane adhesion, and suppressed external stimulus, MNC migration, cell adhesion and leukocyte proliferation pathways in Ly6C^high^ MC, and suppressed myeloid leukocyte migration, collagen catabolic process and humoral immune response pathways in Ly6C^low^ MC. A detailed list of SDE genes and pathway are presented in **Supplementary Table 3**.

For the SDE cytokine genes, we identified 20-activated/20-suppressed pathways in comparison A and B. The top pathways indicated the activation of cytokine production, response to lipopolysaccharide and locomotion, and the suppression of NK cell chemotaxis and leukocyte activation in Ly6C^high^ MC. Specifically, HHcy activated responses to lipopolysaccharide, IL-17 signaling pathway and inflammatory response, and suppressed cytokine production/signaling pathways and adaptive immune response in Ly6C^high^ only in *Cbs* ^-/-^ mice.

In SDE surface marker gene set, we discovered 20-activated/20-suppressed pathways in comparison A and B. The top pathways displayed the activation of myeloid cell and cytokine production, and suppression of lymphocyte activation, hematopoietic cell lineage, and lymphocyte mediated immunity in Ly6C^high^ MC. Specifically, HHcy suppressed regulation of cell adhesion, adaptive immune system and collagen metabolic process in Ly6C^high^ only in *Cbs* ^-/-^ mice.

### Identification of Significantly Differentially Expressed Transcription Factor and Establishment of Transcriptional Regulatory Model for Ly6C^high^ to Ly6C^low^ Monocyte Subset Differentiation

**Figure 5:**
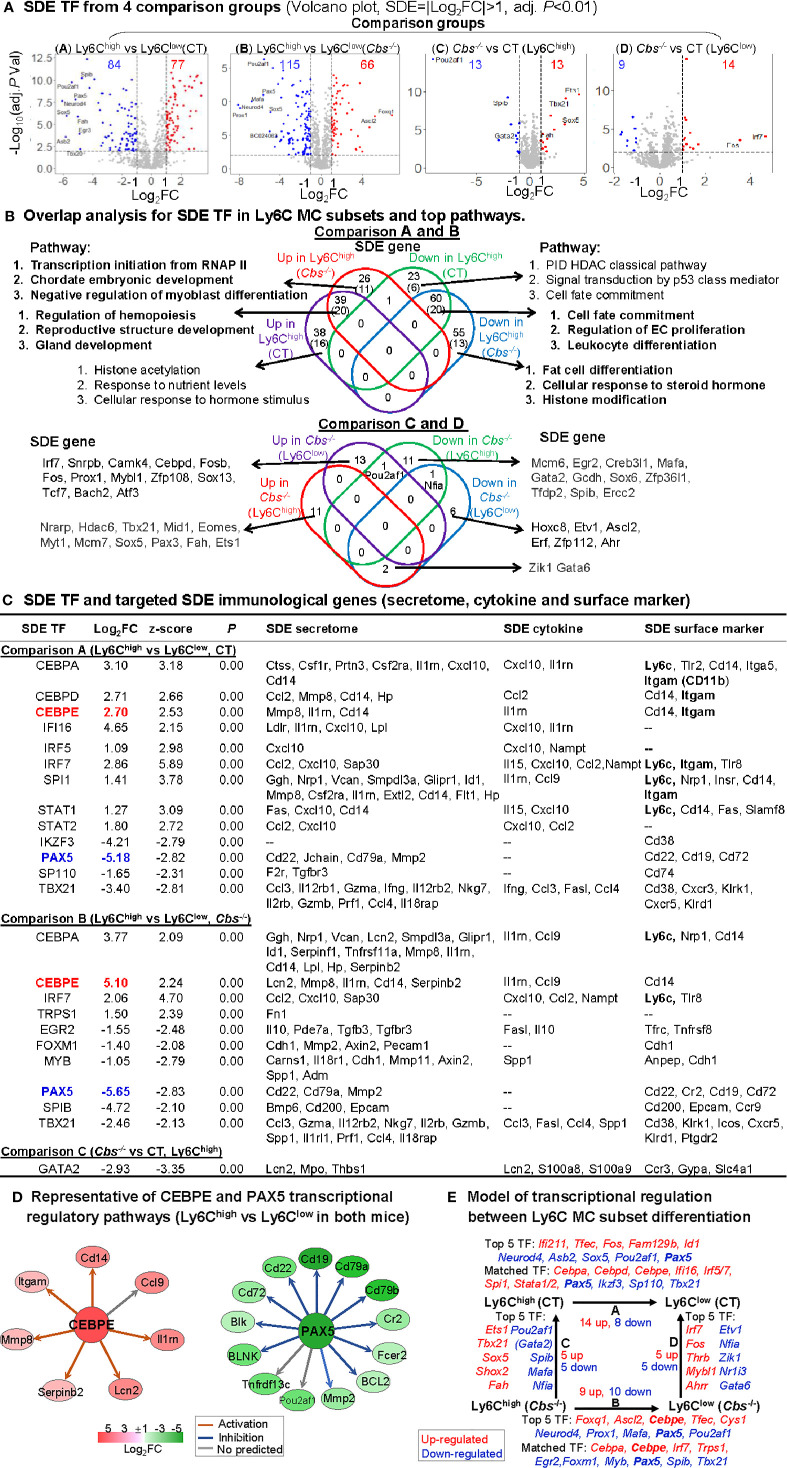
Identification of SDE TF and immunological transcriptional regulatory models. **(A)** SDE TF in four comparison groups. Volcano plot of all genes demonstrates the expression pattern of SDE TF in four comparison groups. Down-regulated SDE TF are highlighted in green and up-regulated in red (|Log_2_FC|>1, adj. *P*<0.01), with Log_2_FC as x-axis and −Log_10_(adjust *P*-value) as y-axis. Top 25 up- and down-regulated SDE TF in all comparisons *via* IPA are listed in **Supplementary Table 2**. **(B)** Overlap analysis for SDE TF in Ly6C MC subsets and top pathways. Venn diagram summarized the total SDE genes and their top 3 pathways in each SDE TF change groups from four pairs of comparisons. Functional pathways were developed by metascape software using the GO database only in SDE set (>20 SDE genes). The top 3 functional pathways are presented. Numbers depict the amount of SDE TF. Numbers in the parentheses describes the number of pathways. A detailed list of SDE TF and pathway in each SDE set are presented in **Supplementary Table 3**. **(C)** SDE TF and targeted SDE immunological genes. SDE immunological genes were matched with SDE TF by IPA upstream analysis. Transcriptional regulatory relationship between SDE TF and SDE immunological genes was justified by correspondence at the same direction (either positive or negative) and overlapped p-value<0.01 and |z-score|>2. Note that Itgam is also known as CD11b, that Ly6c refers to other Ly6 genes (Ly6.2, Ly6C, Ly6C.2, Ly6C antigen, Ly6a2, Ly6al, Ly6b, Ly6c1, Ly6c2, Ly6f, Ly6g, Ly6i). **(D)** Representative of CEBPE and PAX5 transcriptional regulatory pathways (Ly6C^high^ vs. Ly6C^low^ in both mice) CEBPE and PAX5 are used as the representative SDE TF to establish transcriptional regulatory network by using IPA upstream analysis. The corresponding expression levels of targeted SDE genes are indicated by colored nodes. **(E)** Model of transcriptional regulation between Ly6C MC subset differentiation. Model describes potential transcriptional regulatory machinery. In Comparison A, 22 SDE TF (14 up-red and 8 down-blue) are identified in Ly6C^high^ MC subset in CT mice. In Comparison B, 19 SDE TF (nine up and 10 down) are identified in *Cbs^-/-^* Ly6C^high^ MC subset. In Comparison C, 10 SDE TF (five up and five down) are identified in *Cbs^-/-^* Ly6C^high^ MC subset. While, in Comparison D, 10 SDE TF (five up and five down) are identified in *Cbs^-/-^* Ly6C^low^ MC subset. Top 5 SDE TF are indicated in italic letters, and matched SDE TF in the parentheses. Red letter highlighted the representative up-regulated gene. Blue letter highlighted down-regulated genes. Abbreviations are as that in **Figure 2**. RNAP, RNA polymerase, PID, pathway interaction database; HDAC, histone deacetylase. Abbreviation for gene names refer to list in website, https://www.genecards.org/.

As shown in volcano plots in **Figure 5A**, we identified 77-upregulated/84-downregulated, 66-upregulated/115-downregulated, 13-upregulated/13-downregulated, and 14-upregulated/9-downregulated SDE TFs in comparisons A, B, C and D, respectively. From these SDE TFs, we discovered 20-activated/20-suppressed pathways overlapped in Ly6C^high^ MC from both control and *Cbs* ^-/-^ mice (Comparisons A and B) (**Figure 4B** **)**. The top pathways displayed the activation of hemopoiesis, and suppression of cell fate commitment, proliferation and differentiation in Ly6C^high^ MC. Specifically, HHcy activated RNA polymerase II transcription initiation, chordate embryonic development and myoblast differentiation pathways, and suppressed fat cell differentiation, cellular response to steroid hormone, and histone modification pathways in Ly6C^high^ only in *Cbs* ^-/-^ mice.

To identify potential transcriptional regulatory axis in Ly6C MC subset differentiation, the SDE TFs were used to match with corresponding downstream immunological SDE genes by IPA upstream analysis. We found 24 SDE TFs matched and positively associated with various downstream SDE secretome, cytokine and surface marker genes (**Figure 5C**). These were potential transcriptional regulatory mechanisms determining differential immunological features and subset differentiation. Two representative SDE TFs were chosen to describe their relevant transcriptional regulatory axis (**Figure 5D** **)**. CCAAT/enhancer-binding protein Epsilon (Cebpe), also known as CRP1, is expressed primarily in myeloid cells, which is required for the promyelocyte-myelocyte differentiation in myeloid differentiation (50). Cebpe was upregulated by 6.5-fold and 34.3-fold in control and *Cbs^-/-^* Ly6C^high^ MC, which was associated with the upregulation of corresponding targeting secretome (*Lcn2, Mmp8, Il1rn, Cd14* and *Serpinb2*), cytokine (*Il1rn, Ccl9*), surface marker (*Cd14*) in Ly6C^high^ in both mice. Pax5, a member of the paired box (Pax) family of TF, plays an important role in B-cell differentiation and CD19 regulation in B-cell. Pax5 was downregulated by 36.2-fold and 56.2-fold in control and *Cbs^-/-^* Ly6C^high^ MC, which was associated with the downregulation of corresponding targeting TFs (*Ccnd1, Pou2af1, Mmp2*), secretome (*Cd22, Cd79a, Mmp2*), surface marker (*Cd22, Cr2, Cd19, Cd72*) in Ly6C^high^ from both mice.

We presented a model for transcriptional regulatory machinery potentially responsible for MC subset differentiation in **Figure 5E**. The top 5 up/down SDE TFs and matched TFs are depicted. In comparison A, the top upregulated SDE TFs are *Ifi211, Tfec, Fos, Fam129b*, and *Id1*) and the top downregulated SDE TFs are *Neurod4, Asb2, Sox5, Pou2af1*, and *Pax5* in Ly6C^high^ MC from control mice. Nine upregulated SDE TFs (*Cebpa, Cebpd, Cebpe, Ifi16, Irf5/7, Spi1*, and *Stata1/2*) and four downregulated SDE TFs (*Pax5, Ikzf3, Sp110*, and *Tbx21*) were found matched and positively associated with corresponding immunological genes. In comparison B, the top upregulated SDE TFs are *Foxq1, Ascl2, Cebpe, Tfec*, and *Cys1*, and the top downregulated SDE TFs are *Neurod4, Prox1, Mafa, Pax5*, and *Pou2af* in Ly6C^high^ MC from *Cbs^-/-^* mice. Four upregulated SDE TFs (*Cebpa, Cebpe, Irf7*, and *Trps1*) and six downregulated SDE TFs (*Egr2, Foxm1, Myb, Pax5, Spib*, and *Tbx21*) were found matched and positively associated with corresponding immunological genes. In comparison C, the top upregulated SDE TFs are *Ets1, Tbx21, Sox5, Shox2*, and *Fah* and the top downregulated SDE TFs are *Pou2af1, Gata2, Spib, Mafa*, and *Nfia* in Ly6C^high^ MC from *Cbs^-/-^* mice. In comparison D, the top 5 upregulated SDE TFs are *Irf7, Fos, Thrb, Mybl1* and *Ahrr* and the top 5 downregulation SDE TFs are *Etv1, Nfia, Zik1, Nr1i3*, and *Gata* in Ly6C^low^ MC from *Cbs^-/-^* mice.

### Ly6C^high^ Monocyte Presented Downregulated Co-Stimulatory Receptors for Proliferation, and Upregulated Co-Stimulatory Ligands for Antigen Priming and Differentiation

**Figure 6:**
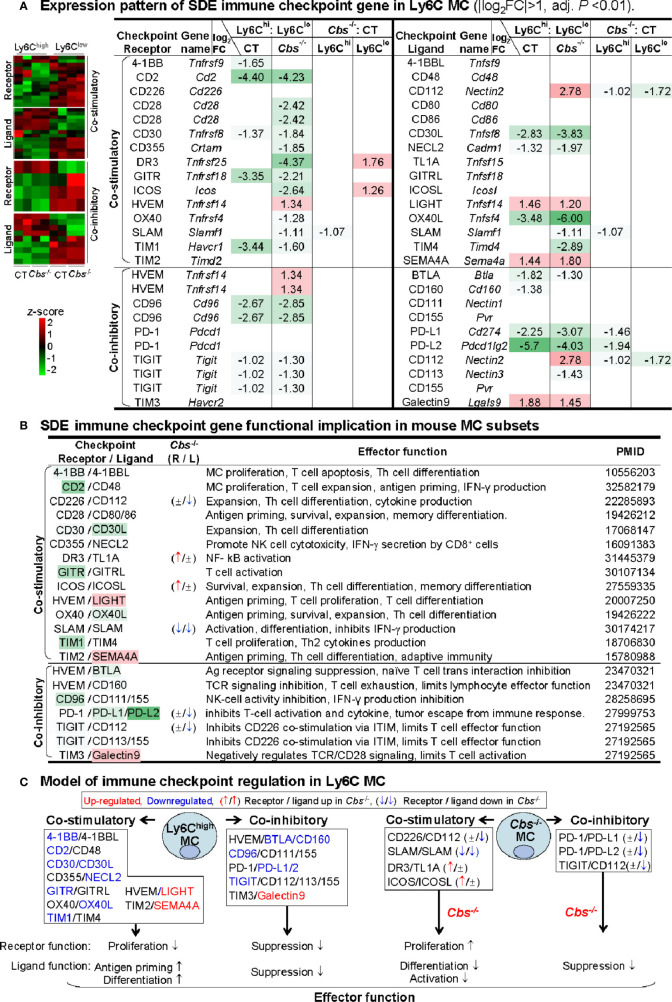
Identification of SDE immune checkpoint gene and function implication in Ly6C MC. **(A)** Expression pattern of SDE immune checkpoint gene in Ly6C MC. Heatmap shows the expression levels of the immune checkpoint gene (receptor and ligand) in Ly6C MC. The color density indicates the average expression of a given gene normalized by z-score. Fifteen pairs of SDE co-stimulatory and 10 pairs of SDE co-inhibitory molecules are identified in four comparison groups. Red-colored background numbers indicate FC>2 (log_2_FC>1). Green-colored background numbers indicate FC<0.5 (log_2_FC<-1). The completed list of Immune checkpoint genes is in **Supplementary Table 4**. **(B)** SDE immune checkpoint gene functional implication in mouse MC subsets. This table describes expression pattern and effector function of SDE immune checkpoint (ligand-receptor) in *Cbs* ^-/-^ Ly6C MC. **(C)** Model of immune checkpoint regulation in Ly6C MC and *Cbs* ^-/-^ mice. In Ly6C^high^ MC, downregulation of co-stimulatory receptor molecules implicates suppressed proliferation and upregulation of ligand molecules implicates increased antigen priming and differentiation. Co-inhibitory molecule change support similar biologic function. *Cbs* ^-/-^ MC presented feature of increased receptor cell proliferation and deceased ligand cell differentiation/activation. Upregulated SDE immune checkpoint molecules are marked in red, downregulated in blue. ↑ refers to induce expression by *Cbs* ^-/-^. ↓ refers to reduce expression by *Cbs* ^-/-^, ± refers to no changes in *Cbs* ^-/-^. NK, natural killer cells; TCR, T-cell receptor; ITIM, immunoreceptor tyrosine-based inhibition motif; Other abbreviations are as that in **Figure 2**.

To test the differential role of Ly6C MC subsets in regulating adaptive immunity, we examined the expression pattern of immune checkpoint molecules. As depicted in **Figure 6A**, 25 out of 49 checkpoint pairs displayed differential expression in Ly6C^high^ and Ly6C^low^ MC subsets. Ly6C^high^ MC expressed relative low levels of both co-stimulatory and co-inhibitory immune checkpoint receptors. A detailed list of immune checkpoint expression was presented in **Supplementary Table 4**.

Based on their differential expression and previously defined function **(** **Figure 6B** **)**, we modeled the functional implication of immune checkpoint in Ly6C MC subsets **(** **Figure 6C** **)**. In Ly6C^high^ MC, four co-stimulatory receptors (4-1BB, CD2, CD30, GITR, and TIM1) and two co-inhibitory receptors (CD96 and TIGIT) were downregulated, which imply suppressed proliferation. In addition, two co-stimulatory ligands (LIGHT and SEMA4A) were upregulated in Ly6C^high^ MC, which imply ligand function for increased antigen priming and differentiation. In *Cbs* ^-/-^ Ly6C^low^ MC, co-stimulatory receptors (DR3 and ICOS) were upregulated, which imply increased proliferation. In *Cbs* ^-/-^ Ly6C^high^ MC, co-inhibitory ligands (CD112, PD-L1/2) were downregulated which imply increased ligand function for differentiation/activation.

### Ly6C^high^ Monocyte Favored to MΦ Differentiation and Ly6C^low^ Monocyte Shared Function With Lymphocyte Subsets

**Figure 7:**
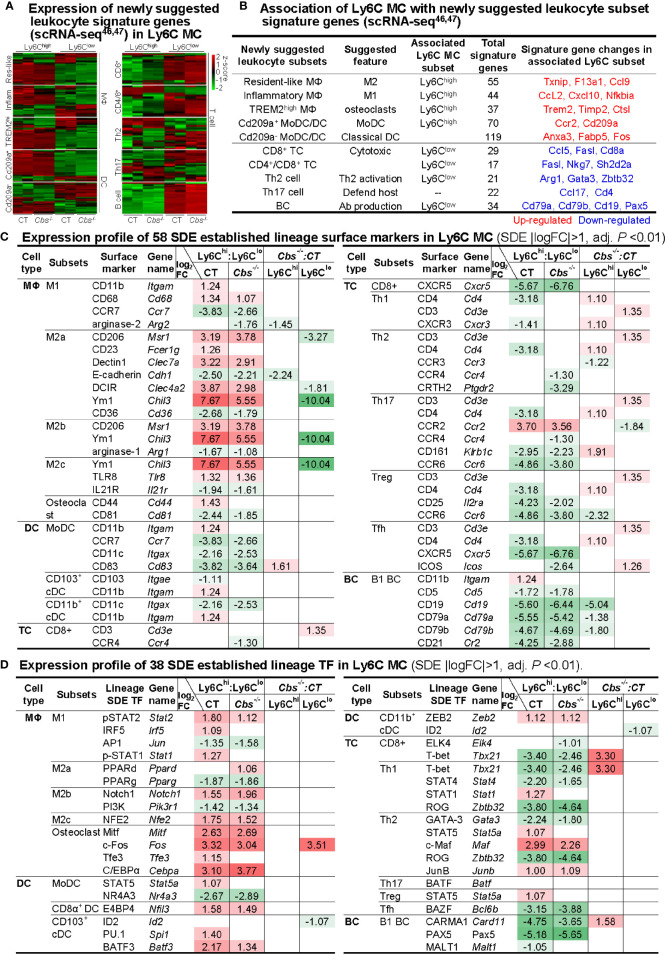
Expression profile of immune cell lineage and subset marker in Ly6C MC subset. **(A)** Expression pattern of newly suggested leukocyte signature genes in Ly6C MC. Heatmap shows the expression levels of the leukocyte signature genes, recently suggested by scRNA-seq study^46^, ^47^ in Ly6C MC. The color density indicates the average expression of a given gene normalized by z-score. Fold change of newly suggested leukocyte signature gene are present in the **Supplementary Table 5**. **(B)** Association of Ly6C MC with newly suggested leukocyte subset signature genes. Connection of the newly suggested leukocyte signature genes with Ly6C MC subsets are established based on their expression pattern in Ly6C MC subsets. **(C)** Expression profile of 58 SDE established lineage surface markers in Ly6C MC. **(D)** Expression profile of 38 SDE established lineage transcription factors in Ly6C MC. Four major immune cell type (MΦ/DC/TC/BC) and their 15 subsets are listed. Lineage SDE surface markers and TF are differentially expressed in four comparison groups in these subsets. Red-colored background numbers indicate FC>2 (log_2_FC>1). Green-colored background numbers indicate FC<0.5 (log_2_FC<-1). Justification for Leukocyte lineage specific TF/surface marker are listed in the **Supplementary Table 6**. scRNA-seq, single-cell RNA sequencing; MC, monocyte, Cbs, Cystathionine β-synthase; MΦ,macrophage; DC, dendritic cell; MoDC monocyte-derived dendritic cell; TC, T cell, T_h_2 cell, T helper 2 cell; BC, B cell; APC, antigen-presenting cells; TF, transcription factor; cDC, classical DCs, pDC, plasmacytoid DC; Treg, Regulatory T cells; Tfh, T follicular helper.

To examine the potential plasticity of Ly6C MC subsets, we first analyzed the expression pattern of newly suggested leukocyte signature genes from recent scRNA-seq studies (46, 47). Ly6C^high^ MC expressed high levels of myeloid cell (MΦ and DC) signature genes in both mice (**Figures 7A, B** **)**. Differently, Ly6C^low^ MC expressed high levels of lymphocyte (T- and B-cell) signature genes, especially that of CD8^+^ T-cell and B-cell (**Figures 7A, B** **)**. Interestingly, Ly6C^high^ MC expressed high levels of osteoclast TFs (*Cebpa, Fos, Tfe3*, and *Mitf*) and surface marker CD44, and osteoclast-like TREM2^high^ MΦ signature osteoclastogenesis gene (*Trem2, Fcer1g, Timp2*, and *Ctsl*). The details of newly suggested leukocyte signature genes deferentially expressed in Ly6C^high^ and Ly6C^low^ MC were listed in the **Supplementary Table 5**.

Further, we examined the expression of established lineage/subset TF and surface marker in Ly6C MC. MΦ surface markers (CXCL10, Ym1, and CD206) and myeloid lineage TFs (*Cebpa, c-Fos*, and *Spi1*) were highly expressed in Ly6C^high^ MC in both mice. While, lymphocyte surface markers (CD4, CD25, CD161, CD5, CD19, CD21, CD79a, and CD79b) and lymphocyte lineage TFs (*T-bet, Rog, Carma1*, and *Pax5*) were preferentially expressed in Ly6C^low^ MC in both mice **(** **Figures 7C, D** **)**. Specifically, CD3, a T-cell receptor involved in activating both cytotoxic T-cell and T helper (Th) cells, was upregulated by *Cbs* ^-/-^ in Ly6C^low^ MC (Comparison D). Literature justification and designation of TFs and surface markers for leukocyte subsets are provided in the **Supplementary Table 6**. Expression change and function implication of SDE cytokine genes in Ly6C MC were presented in **Supplementary Table 7**.

## Discussion

Mouse MC are classified into inflammatory Ly6C^high^ and anti-inflammatory Ly6C^low^ subsets. However, the molecular mechanism underlying MC subset differentiation remains unclear, and functional features of MC subsets have not been systemically investigated. This study established transcription profiles of flow cytometry sorted Ly6C^high^ and Ly6C^low^ MC subsets from control and HHcy *Cbs* ^-/-^ mice and examined their functional features and transcriptional regulatory pathways by performing intensive bioinformatic analysis and literature integration. We have 6 major findings: 1) Ly6C^high^ MC showed enriched inflammatory pathways, whereas Ly6C^low^ MC displayed activated lymphocyte immunity pathways in both control and *Cbs* ^-/-^ mice. 2) Identified SDE TFs and their corresponding targeted SDE genes in Ly6C MC subset from both mice. 3) Ly6C^high^ MC presented downregulated immune checkpoint receptor-directed immune cell proliferation, and upregulated ligand-triggered antigen priming and differentiation. 4) Ly6C^high^ MC preferentially expressed MΦ and osteoclast markers, whereas Ly6C^low^ MC expressed higher levels of lymphocyte subsets markers. 5) HHcy in *Cbs* ^-/-^ mice reinforced the inflammatory response in Ly6C^high^ MC, but promoted functional adaptation of lymphocytes in Ly6C^low^ MC. 6) We established 3 groups of hypothetic molecular signaling models. The first model described transcriptional regulatory mechanism of Ly6C^high^ to Ly6C^low^ MC subset differentiation. These include SDE immunological gene and their regulatory SDE TFs. The second model was for immune checkpoint molecular alteration and function connection in MC subset. The third model summarized the potential molecular mechanism regulating Ly6C^high^ MC to MΦ subset differentiation and Ly6C^low^ MC to lymphocyte functional adaptation. Our findings provide important insights into the understanding of molecule and functional features of MC subsets.

Our study emphasized that inflammatory pathways were enriched in Ly6C^high^ MC and Ly6C^low^ MC presented features of lymphocyte immunity activation **(** **Figures 3** and **4** **)**. Ly6C^high^ MC from both mice displayed elevated interferon, inflammasome, neutrophil degranulation, lysosome, cytokine production/receptor interaction and myeloid cell activation pathways. This is consistent with previous findings showing that Ly6C^high^ MC are rapidly recruited to sites of inflammation and releasing proinflammatory cytokines, such as type I interferon (IFN-I), IL-1, IL-6, IL-8, TNF-α, and MCP-1 (4, 51–55). It was reported that Ly6C^high^ MC coordinates the innate immune response through inflammasome activation following exposure to pathogen-, damage-associated molecular patterns (PAMP, DAMP) and metabolic-associated danger signals (MADS) (25, 32, 56). Lysosomal activity is a new feature of Ly6C^high^ MC, which implies enhance function of endocytosis and autophagia, and molecule degradation (57). Phagocytic features of Ly6C^high^ MC were connected with high lysosomal activity (3, 58).

Our data suggested that 9 SDE TFs (*Cebpa, Cebpd, Cebpe, Irf5/7, Ifi16, Spi1*, and *Stat1/2*) are potentially involved in Ly6C^high^ MC generation and responsible for the immunological features in control mice (**Figure 5C**). We and others have reported that CEBPα and CEBPδ were enriched in Ly6C^high^ MC (11, 38). CEBPα binds to the *Ly6c* promoter and its expression was elevated and synergistically increased in HHcy and Type 2 Diabetes Mellitus mice (38). We found PU.1 (encoded by *Spi1* gene) was increased by 2.66-fold in Ly6C^high^ MC in control mice. PU.1 was a critical lineage determining TF for both myeloid and lymphoid cell development as PU.1-deficient mice lack MC, granulocytes and B-cells (3, 59). PU.1 can transactivate other TFs (e.g., CEBPα, CEBPβ, IRF proteins, c-Jun, JunB) to regulate subset differentiation (60). Upregulation of Irf7 by 7.26-fold in Ly6C^high^ MC in control mice may be related with their function towards MΦ differentiation. This is supported by IRF-7 overexpression-induced MC differentiation to MΦ in U937 and HL60 cells (61).

We found that CEBPα, Irf7, PU.1 and Stat1 were *Ly6c* TFs and positively associated with *Ly6c* expression. They are strong candidate determining Ly6C^high^ MC generation. Other upregulated TFs in Ly6C^high^ MC are also potentially responsible for Ly6C^high^ MC generation, for example, the top 5 TFs (*Ifi211, Tfec, Fos, Fam129b*, and *Id1*) listed in **Figure 5E**. Under homeostasis, classical Ly6C^high^ MC in blood reduces the expression of Ly6C and becomes non-classical Ly6C^low^ MC (7, 15). We proposed that downregulated TFs in Ly6C^high^ MC are possible regulators determining Ly6C^high^ MC to Ly6C^low^ MC differentiation. The top 4 downregulated TFs (*Neurod4, Asb2, Sox5* and *Pou2af1*) and 2 matched TFs *Pax5* and *Tbx21* represented potential general transcriptional mechanism for Ly6C^high^ MC to Ly6C^low^ MC differentiation. Pax5 plays a crucial role in the commitment of BM multipotent progenitor cells to the B-lymphoid lineages. It has been shown that, except for B-cell lineage, other hemopoietic lineages develop normally in *Pax5*-deficient mice (62). T-bet (encoded by the *Tbx21* gene) controlled IFN-γ expression in CD4^+^ T-cell, and was reported recently to be expressed in human MC (63). Lack of Tbx21 reduces monocytic interleukin-12 formation and accelerates thrombus resolution in deep vein thrombosis (64). Overall, TFs (*Pax5* and *Tbx21*) were previously thought as lymphocyte lineage-specific TF, but their role in regulating MC differentiation remains to be addressed.

Interestingly, Ly6C^high^ MC expressed lower levels of co-stimulatory receptors (4-1BB, CD2, CD30, GITR and TIM1), which direct cell proliferation **(** **Figure 6C** **)**. Multiple evidence showed that the activation of GITR, 4-1BB (also termed as ILA/CD137) and TIM1 induces MC/MΦ proliferation (65–68). TNF/TNFR family members 4-1BB, GITR and CD30, TIM1, and CD2 have been shown to promote T-cell (effector and memory) activation in mouse models (65, 66, 69–71). Low levels of CD2 and CD30 have been described in activated MC (71, 72). Taken together, Ly6C^high^ MC has a lower proliferative potential based on co-stimulatory receptor expression pattern.

The upregulation of co-stimulatory ligands (LIGHT and SEMA4A) in Ly6C^high^ MC led us to hypothesize that Ly6C^high^ MC presents high activity of antigen priming and differentiation. LIGHT/HVEM engagement promotes T-cell priming and differentiation (73, 74). During viral infection, LIGHT are induced by IFN‐γ on MC‐derived cells (75). High level expression of Sema4A was found on Ly6C^high^ MC (76). Sema4A-deficient mice exhibit defective Th1 responses and impaired antigen-specific T-cell priming and antibody response against T-cell-dependent antigens (76). These findings suggested a key role for Ly6C^high^ MC in the regulation of T-cell immunity and may provide new insights into development of more effective therapies for diseases in which T-cell has an important role.

**Figure 8:**
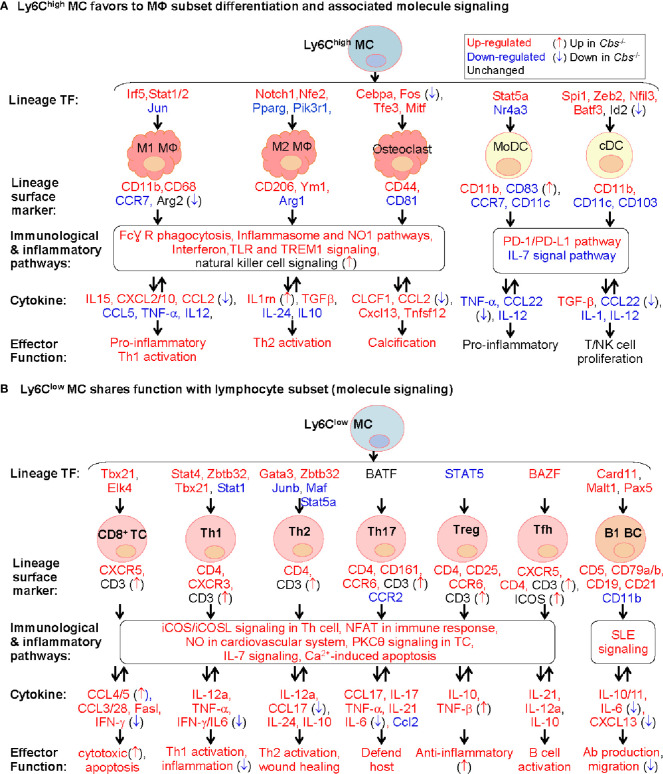
Molecule signaling of Ly6C MC to MΦ subset differentiation and to lymphocyte subset functional adaptation. We established two models for molecule signaling of MC differentiation based on their preferential expression of lineage signature TF, surface marker and cytokine using information extracted from **Figures 3**, **5**, and **7**. **(A)** Ly6C^high^ MC favors to MΦ subset differentiation and associated molecule signaling. Ly6C^high^ MC preferentially expressed lineage signature TF genes of MΦ/DC subsets, suggesting their potential differentiation to MΦ. The indicated immunological and inflammatory pathways lead to various changes of cytokines production, and effector function including T/NK cell proliferation, inflammatory response and calcification. *Cbs* ^-/-^ Ly6C^high^ MC exhibited inflammatory cytokine production. **(B)** Ly6C^low^ MC shares function with lymphocyte subset (molecule signaling). Ly6C^low^ MC preferentially expressed lineage signature TF genes of B/T cell subsets, suggesting their potential functional adaptation to lymphocyte subsets. The indicated immunological and inflammatory pathways lead to various changes of cytokines attributed to increased T/B cell activation, host defend, wound healing and anti-inflammatory responds. *Cbs* ^-/-^ Ly6C^low^ MC exhibited enhance T/B cell activation potential. Expression change and function implication of SDE cytokine genes in Ly6C MC were presented in **Supplementary Table 7**. MC, monocyte; DC, dendritic cell; MΦ, macrophage; TREM1, the triggering receptor expressed on myeloid cells; NK, natural killer, TC, T cell; Th1, T helper 1 cell; Tfh, T follicular helper; BC, B cell, NFAT, Ca^2+^, Calcium; SLE, systemic lupus erythematosus, IL-7, Interleukin 7; NFAT, nuclear factor of activated T-cells; nNOS, neuronal nitric oxide synthase.

As illustrated in **Figure 8A**, our study provides evidence to support a model that Ly6C^high^ MC favors to differentiate to MΦ, but not to DC. This is based on Ly6C^high^ MC expressed high levels of inflammatory cytokine (IL15, CXCL2/10, and CCL2) and MΦ specific markers, including M1 MΦ surface marker (CD11b and CD68) and TFs (*Irf5* and *Stat1/2*), and M2 MΦ TFs (*Notch1* and *Nfe2*) and surface marker (CD206 and Ym1). Whereas, Ly6C^high^ MC exhibited inconsistent changes for DC lineage markers.

Although the fate and mechanism underlying Ly6C^high^ MC differentiation is unclear, a more common postulation is that Ly6C^high^ MC tend to differentiate into M1 MΦ, but Ly6C^low^ MC to M2 MΦ (37, 77). It is suggested that Ly6C^high^ MC may be primed to differentiate into Ly6C^low^ MC, or infiltrated into tissues to develop specific tissue MC-derived cells (3, 4, 17, 78). It was shown that continued recruitment of Ly6C^high^ MC and their differentiation to M2 rather than M1 MΦ are required for resolution of atherosclerotic inflammation and plaque regression (46, 79). The destiny of Ly6C^high^ MC differentiation may vary under different microenvironment. Details presented in **Figure 8A** provide important insights for molecular pathways underlying Ly6C^high^ MC to MΦ differentiation.

Based on the high levels of osteoclast TFs, surface marker and osteoclast-like TREM2^high^ MΦ signature genes in Ly6C^high^ MC, we proposed that Ly6C^high^ MC is a precursor of osteoclasts. Osteoclasts contribute to vascular calcification, which causes local tissue stress and plaque instability (80). Like MΦ, osteoclasts are derived from MC precursors in chronic inflammatory conditions and required 2 main cytokines (CSF1 and RANKL) and 4 TFs (*Cebpa, Fos, Tfe3*, and *Mitf*) (81, 82). Our data is in good accordance with previous finding showing that Ly6C^high^ MC, but not Ly6C^low^, differentiate into osteoclast in arthritis bone erosion (18, 83). Taken together, we hypothesize that inflammatory MC subset can be differentiate to osteoclasts and contribute to tissue calcification in inflammatory condition and chronic disease.

We promoted a model for Ly6C^low^ MC to lymphocyte subsets functional adaptation according to their preferential express of T-cell specific surface markers, lineage TFs and checkpoint receptor, and their associated T-cell-related effector function (**Figure 8B**). The classical road map of immune cell differentiation describes that lymphoid progenitor lineages segregate from myelo-erythroid (ME) in hematopoietic stem cells. However, the ‘myeloid-based model’ suggested that myeloid cell can also be generated from myeloid-T progenitor and myeloid-B progenitor, which is derived from common myelo-lymphoid progenitor (84, 85). Recent evidence suggested that early pro-B-cell can give rise to either MC-derived MΦ or tissue-specific MΦ during tissue homeostasis and inflammation (86). Evidence for myeloid to lymphoid lineage differentiation and function adaptation is absent. Our study, for the first time, provide evidence of Ly6C^low^ MC to lymphocyte functional adaptation.

Our data demonstrated that HHcy in *Cbs* ^-/-^ mice reinforced inflammatory and immunological responses in Ly6C^high^ MC by upregulating inflammatory TFs (*Ets1, Tbx21* and *Sox5*) and downregulating co-inhibitory checkpoint (CD112 and PD-L1/2). The TF *Ets1* has been shown to regulate genes (VCAM1 and MCP-1) involved in vascular inflammation (87). *Tbx21* ^-/-^ mice exhibited reduced IFN-γ and IL-17 expression in CD8^+^ T-cell and inflammation in gut and peripheral joint (88). The TF *Sox5* was related with inflammatory response in rheumatoid arthritis fibroblast-like synoviocytes (89). Further, immune checkpoint ligand molecules (CD112 and PD-L1/2) was suppressed in *Cbs* ^-/-^ Ly6C^high^ MC intimated activation and differentiation. CD112 transduces stimulatory signal by binding to CD226, while transduces suppressive and anti-inflammatory signal by binding to TIGIT (90, 91). Engagement of PD-1 by its ligands (PD-L1/2) induces suppressive signal to inhibit T-cell proliferation, cytokine production and cytotoxic activity (92, 93). These evidences supported our conclusion that HHcy reinforced inflammatory and immunological response in Ly6C^high^ MC.

Our data also suggested that HHcy further strengthened Ly6C^low^ MC to lymphocytes functional adaptation by upregulating surface marker CD3, co-stimulatory checkpoint (DR3, ICOS) and TF *Fos*. CD3 complexes with T-cell receptor contributing to antigen recognition (94). The ligation of immune checkpoint receptor DR3 with TL1A exerts activation and differentiation in immune cell, including Th and T-reg cell (95). ICOS regulates the differentiation and maintenance of Tfh cells (96), which helps B-cells to form germinal centers and differentiate into plasma cells and memory B-cell for high affinity antibody production (96, 97). TF *Fos* plays a central role in nuclear factor of activated T-cell (NFAT) complex formation which involved in cell proliferation, differentiation and tumor progression (98–100). This evidence supports the notion that HHcy promoted lymphocytes functional adaptation in Ly6C^low^ MC.

In conclusion, our study, for the first time, demonstrated that Ly6C^high^ MC displayed enriched inflammatory pathways, immune checkpoint molecules for suppressed proliferation and increased antigen priming, and demonstrated the potential to differentiate into MΦ and osteoclast. Ly6C^low^ MC manifested activated T-cell signal pathways and potentially can adapt the function of lymphocytes. HHcy in *Cbs* ^-/-^ mice reinforced inflammatory response in Ly6C^high^ MC and strengthened lymphocytes functional adaptation in Ly6C^low^ MC.

## Data Availability Statement

The data present in the study are deposited in the Gene Expression Omnibus (GEO) repository under the accession number GEO:GSE165879.

## Ethics Statement

The animal study was reviewed and approved by the Temple University Institutional Animal Care and Use Committee (IACUC).

## Author Contributions

PY analyzed the data, drafted and participated in preparing all figures and manuscript. LL conducted the bioinformatics analyses. LS participated in data analysis and some part of manuscript preparation. PF isolated MC subsets from mice and designed RNA-Seq analysis. JS and WS participated in some of data analysis and provided editing assistance. NS, YJ and XQ provided intellectual and data analysis support. QW and XY provided strong intellectual and data analysis support. HW designed the study, supervised the project and prepared the manuscript. and All authors contributed to the article and approved the submitted version.

## Funding

This work was supported in part by the National Institutes of Health (NIH) grants HL82774, HL-110764, HL130233, HL131460, DK104114, DK113775, and HL131460 to HW.

## Conflict of Interest

The authors declare that the research was conducted in the absence of any commercial or financial relationships that could be construed as a potential conflict of interest.

## Abbreviations

MC, Monocyte; HHcy, Hyperhomocysteinemia; Cbs-/-, Cystathionine β-synthase gene-deficient; SDE, Significantly differentially expressed; TF, Transcription factor; Ly6C, Lymphocyte antigen 6 complex, locus C; BM, Bone marrow; DC, Dendritic cells; Mϕ, Macrophages; NK, Natural killer; IL, Interleukin; MCP, Monocyte chemoattractant protein; CD, Cluster of differentiation; RNA-seq, RNA sequencing; scRNA-seq, Single-cell RNA sequencing; Tg-hCBS, Human CBS transgenic; FACS, Fluorescence-activated cell sorting; PBS, Phosphate‐buffered saline; EDTA, Ethylenediaminetetraacetic acid; FC, Fold change; PCA, Principle components analysis; PC, Principal components; IPA, Ingenuity Pathway Analysis; IFN-I, Type I interferon; PAMP, Pathogen-associated molecular pattern; DAMP, Damage-associated molecular pattern; MADS, Metabolic-associated danger signals; Th, T helper; Tfh, T follicular helper; T-reg, Regulatory T; ME, Myelo-erythroid.
