# Supplementary material for: Immunological Feature and Transcriptional Signaling of Ly6C Monocyte Subsets From Transcriptome Analysis in Control and Hyperhomocysteinemic Mice
Source: Front Immunol. 2021 Feb 25;12:632333. doi: 10.3389/fimmu.2021.632333 (PMC7947624; doi:10.3389/fimmu.2021.632333)
Supplement: Supplementary file 1 [file Presentation_1.pptx]

## Slide 1
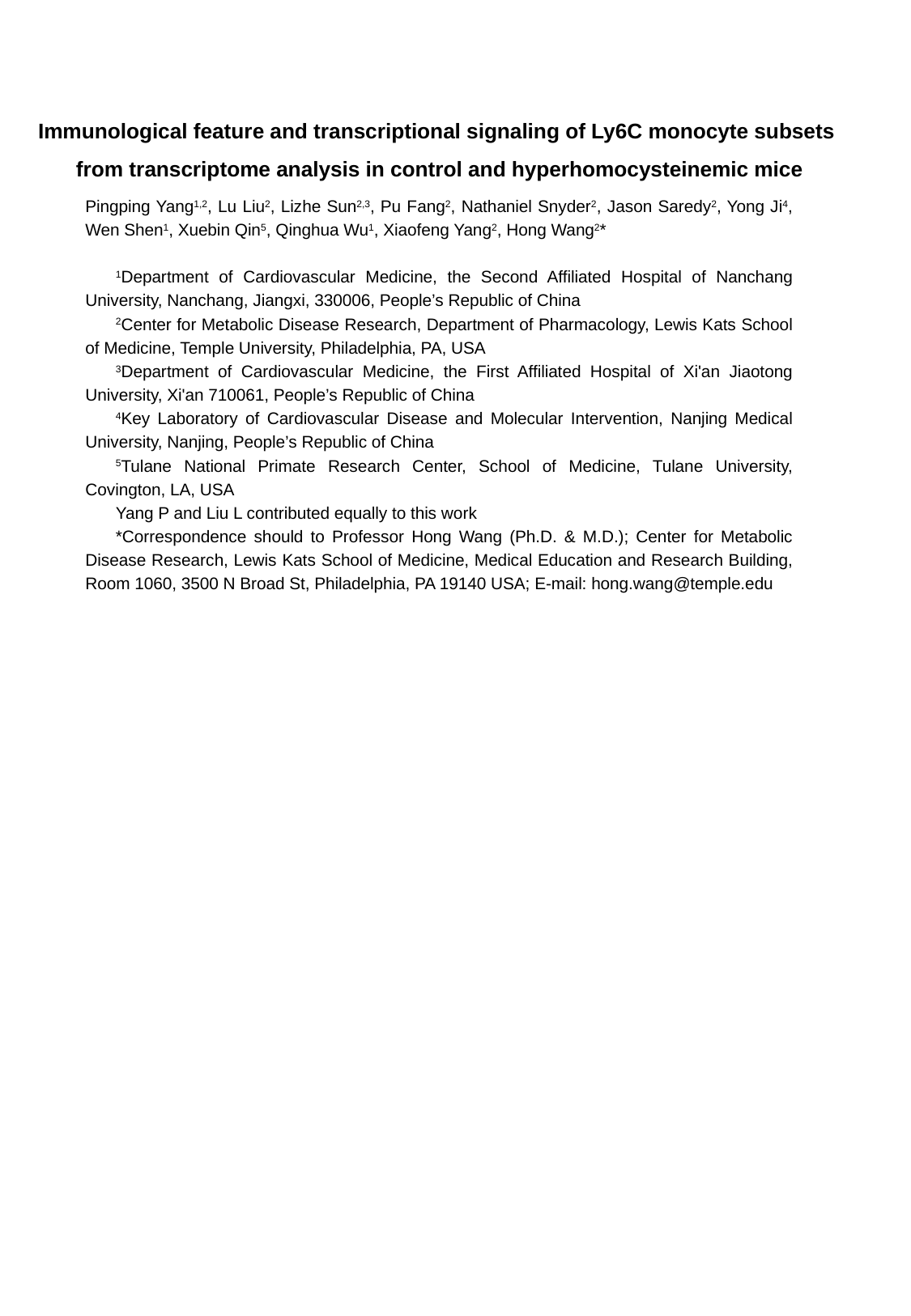

Immunological feature and transcriptional signaling of Ly6C monocyte subsets
from transcriptome analysis in control and hyperhomocysteinemic mice
Pingping Yang1,2, Lu Liu2, Lizhe Sun2,3, Pu Fang2, Nathaniel Snyder2, Jason Saredy2, Yong Ji4, Wen Shen1, Xuebin Qin5, Qinghua Wu1, Xiaofeng Yang2, Hong Wang2*
1Department of Cardiovascular Medicine, the Second Affiliated Hospital of Nanchang University, Nanchang, Jiangxi, 330006, People’s Republic of China
2Center for Metabolic Disease Research, Department of Pharmacology, Lewis Kats School of Medicine, Temple University, Philadelphia, PA, USA
3Department of Cardiovascular Medicine, the First Affiliated Hospital of Xi'an Jiaotong University, Xi'an 710061, People’s Republic of China
4Key Laboratory of Cardiovascular Disease and Molecular Intervention, Nanjing Medical University, Nanjing, People’s Republic of China
5Tulane National Primate Research Center, School of Medicine, Tulane University, Covington, LA, USA
Yang P and Liu L contributed equally to this work
*Correspondence should to Professor Hong Wang (Ph.D. & M.D.); Center for Metabolic Disease Research, Lewis Kats School of Medicine, Medical Education and Research Building, Room 1060, 3500 N Broad St, Philadelphia, PA 19140 USA; E-mail: hong.wang@temple.edu

## Slide 2
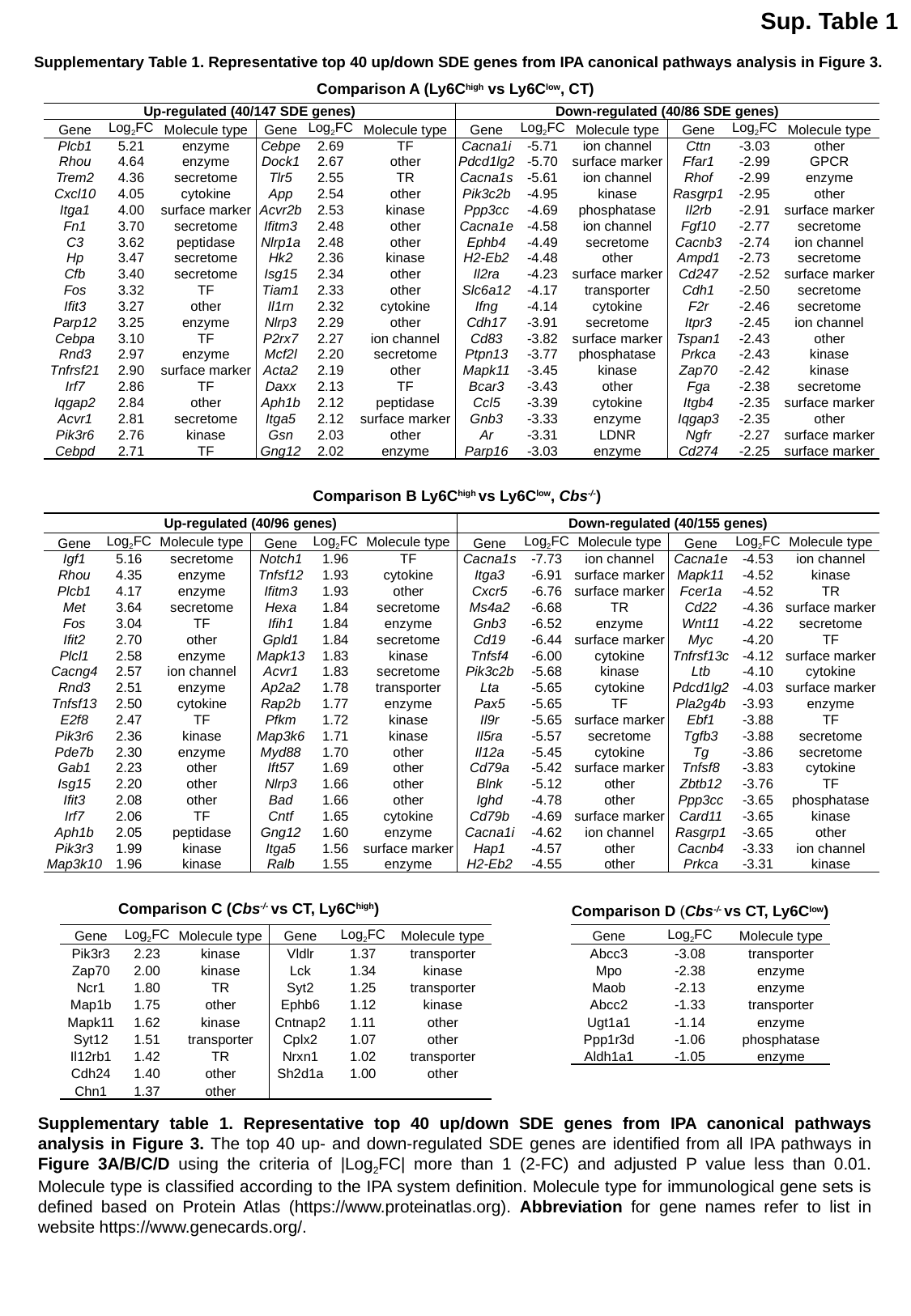

Sup. Table 1
Supplementary Table 1. Representative top 40 up/down SDE genes from IPA canonical pathways analysis in Figure 3.
Comparison A (Ly6Chigh vs Ly6Clow, CT)
| Up-regulated (40/147 SDE genes) | | | | | | Down-regulated (40/86 SDE genes) | | | | | |
| --- | --- | --- | --- | --- | --- | --- | --- | --- | --- | --- | --- |
| Gene | Log2FC | Molecule type | Gene | Log2FC | Molecule type | Gene | Log2FC | Molecule type | Gene | Log2FC | Molecule type |
| Plcb1 | 5.21 | enzyme | Cebpe | 2.69 | TF | Cacna1i | -5.71 | ion channel | Cttn | -3.03 | other |
| Rhou | 4.64 | enzyme | Dock1 | 2.67 | other | Pdcd1lg2 | -5.70 | surface marker | Ffar1 | -2.99 | GPCR |
| Trem2 | 4.36 | secretome | Tlr5 | 2.55 | TR | Cacna1s | -5.61 | ion channel | Rhof | -2.99 | enzyme |
| Cxcl10 | 4.05 | cytokine | App | 2.54 | other | Pik3c2b | -4.95 | kinase | Rasgrp1 | -2.95 | other |
| Itga1 | 4.00 | surface marker | Acvr2b | 2.53 | kinase | Ppp3cc | -4.69 | phosphatase | Il2rb | -2.91 | surface marker |
| Fn1 | 3.70 | secretome | Ifitm3 | 2.48 | other | Cacna1e | -4.58 | ion channel | Fgf10 | -2.77 | secretome |
| C3 | 3.62 | peptidase | Nlrp1a | 2.48 | other | Ephb4 | -4.49 | secretome | Cacnb3 | -2.74 | ion channel |
| Hp | 3.47 | secretome | Hk2 | 2.36 | kinase | H2-Eb2 | -4.48 | other | Ampd1 | -2.73 | secretome |
| Cfb | 3.40 | secretome | Isg15 | 2.34 | other | Il2ra | -4.23 | surface marker | Cd247 | -2.52 | surface marker |
| Fos | 3.32 | TF | Tiam1 | 2.33 | other | Slc6a12 | -4.17 | transporter | Cdh1 | -2.50 | secretome |
| Ifit3 | 3.27 | other | Il1rn | 2.32 | cytokine | Ifng | -4.14 | cytokine | F2r | -2.46 | secretome |
| Parp12 | 3.25 | enzyme | Nlrp3 | 2.29 | other | Cdh17 | -3.91 | secretome | Itpr3 | -2.45 | ion channel |
| Cebpa | 3.10 | TF | P2rx7 | 2.27 | ion channel | Cd83 | -3.82 | surface marker | Tspan1 | -2.43 | other |
| Rnd3 | 2.97 | enzyme | Mcf2l | 2.20 | secretome | Ptpn13 | -3.77 | phosphatase | Prkca | -2.43 | kinase |
| Tnfrsf21 | 2.90 | surface marker | Acta2 | 2.19 | other | Mapk11 | -3.45 | kinase | Zap70 | -2.42 | kinase |
| Irf7 | 2.86 | TF | Daxx | 2.13 | TF | Bcar3 | -3.43 | other | Fga | -2.38 | secretome |
| Iqgap2 | 2.84 | other | Aph1b | 2.12 | peptidase | Ccl5 | -3.39 | cytokine | Itgb4 | -2.35 | surface marker |
| Acvr1 | 2.81 | secretome | Itga5 | 2.12 | surface marker | Gnb3 | -3.33 | enzyme | Iqgap3 | -2.35 | other |
| Pik3r6 | 2.76 | kinase | Gsn | 2.03 | other | Ar | -3.31 | LDNR | Ngfr | -2.27 | surface marker |
| Cebpd | 2.71 | TF | Gng12 | 2.02 | enzyme | Parp16 | -3.03 | enzyme | Cd274 | -2.25 | surface marker |
Comparison B Ly6Chigh vs Ly6Clow, Cbs-/-)
| Up-regulated (40/96 genes) | | | | | | Down-regulated (40/155 genes) | | | | | |
| --- | --- | --- | --- | --- | --- | --- | --- | --- | --- | --- | --- |
| Gene | Log2FC | Molecule type | Gene | Log2FC | Molecule type | Gene | Log2FC | Molecule type | Gene | Log2FC | Molecule type |
| Igf1 | 5.16 | secretome | Notch1 | 1.96 | TF | Cacna1s | -7.73 | ion channel | Cacna1e | -4.53 | ion channel |
| Rhou | 4.35 | enzyme | Tnfsf12 | 1.93 | cytokine | Itga3 | -6.91 | surface marker | Mapk11 | -4.52 | kinase |
| Plcb1 | 4.17 | enzyme | Ifitm3 | 1.93 | other | Cxcr5 | -6.76 | surface marker | Fcer1a | -4.52 | TR |
| Met | 3.64 | secretome | Hexa | 1.84 | secretome | Ms4a2 | -6.68 | TR | Cd22 | -4.36 | surface marker |
| Fos | 3.04 | TF | Ifih1 | 1.84 | enzyme | Gnb3 | -6.52 | enzyme | Wnt11 | -4.22 | secretome |
| Ifit2 | 2.70 | other | Gpld1 | 1.84 | secretome | Cd19 | -6.44 | surface marker | Myc | -4.20 | TF |
| Plcl1 | 2.58 | enzyme | Mapk13 | 1.83 | kinase | Tnfsf4 | -6.00 | cytokine | Tnfrsf13c | -4.12 | surface marker |
| Cacng4 | 2.57 | ion channel | Acvr1 | 1.83 | secretome | Pik3c2b | -5.68 | kinase | Ltb | -4.10 | cytokine |
| Rnd3 | 2.51 | enzyme | Ap2a2 | 1.78 | transporter | Lta | -5.65 | cytokine | Pdcd1lg2 | -4.03 | surface marker |
| Tnfsf13 | 2.50 | cytokine | Rap2b | 1.77 | enzyme | Pax5 | -5.65 | TF | Pla2g4b | -3.93 | enzyme |
| E2f8 | 2.47 | TF | Pfkm | 1.72 | kinase | Il9r | -5.65 | surface marker | Ebf1 | -3.88 | TF |
| Pik3r6 | 2.36 | kinase | Map3k6 | 1.71 | kinase | Il5ra | -5.57 | secretome | Tgfb3 | -3.88 | secretome |
| Pde7b | 2.30 | enzyme | Myd88 | 1.70 | other | Il12a | -5.45 | cytokine | Tg | -3.86 | secretome |
| Gab1 | 2.23 | other | Ift57 | 1.69 | other | Cd79a | -5.42 | surface marker | Tnfsf8 | -3.83 | cytokine |
| Isg15 | 2.20 | other | Nlrp3 | 1.66 | other | Blnk | -5.12 | other | Zbtb12 | -3.76 | TF |
| Ifit3 | 2.08 | other | Bad | 1.66 | other | Ighd | -4.78 | other | Ppp3cc | -3.65 | phosphatase |
| Irf7 | 2.06 | TF | Cntf | 1.65 | cytokine | Cd79b | -4.69 | surface marker | Card11 | -3.65 | kinase |
| Aph1b | 2.05 | peptidase | Gng12 | 1.60 | enzyme | Cacna1i | -4.62 | ion channel | Rasgrp1 | -3.65 | other |
| Pik3r3 | 1.99 | kinase | Itga5 | 1.56 | surface marker | Hap1 | -4.57 | other | Cacnb4 | -3.33 | ion channel |
| Map3k10 | 1.96 | kinase | Ralb | 1.55 | enzyme | H2-Eb2 | -4.55 | other | Prkca | -3.31 | kinase |
Comparison C (Cbs-/- vs CT, Ly6Chigh)
Comparison D (Cbs-/- vs CT, Ly6Clow)
| Gene | Log2FC | Molecule type | Gene | Log2FC | Molecule type |
| --- | --- | --- | --- | --- | --- |
| Pik3r3 | 2.23 | kinase | Vldlr | 1.37 | transporter |
| Zap70 | 2.00 | kinase | Lck | 1.34 | kinase |
| Ncr1 | 1.80 | TR | Syt2 | 1.25 | transporter |
| Map1b | 1.75 | other | Ephb6 | 1.12 | kinase |
| Mapk11 | 1.62 | kinase | Cntnap2 | 1.11 | other |
| Syt12 | 1.51 | transporter | Cplx2 | 1.07 | other |
| Il12rb1 | 1.42 | TR | Nrxn1 | 1.02 | transporter |
| Cdh24 | 1.40 | other | Sh2d1a | 1.00 | other |
| Chn1 | 1.37 | other | | | |
| Gene | Log2FC | Molecule type |
| --- | --- | --- |
| Abcc3 | -3.08 | transporter |
| Mpo | -2.38 | enzyme |
| Maob | -2.13 | enzyme |
| Abcc2 | -1.33 | transporter |
| Ugt1a1 | -1.14 | enzyme |
| Ppp1r3d | -1.06 | phosphatase |
| Aldh1a1 | -1.05 | enzyme |
Supplementary table 1. Representative top 40 up/down SDE genes from IPA canonical pathways analysis in Figure 3. The top 40 up- and down-regulated SDE genes are identified from all IPA pathways in Figure 3A/B/C/D using the criteria of |Log2FC| more than 1 (2-FC) and adjusted P value less than 0.01. Molecule type is classified according to the IPA system definition. Molecule type for immunological gene sets is defined based on Protein Atlas (https://www.proteinatlas.org). Abbreviation for gene names refer to list in website https://www.genecards.org/.

## Slide 3
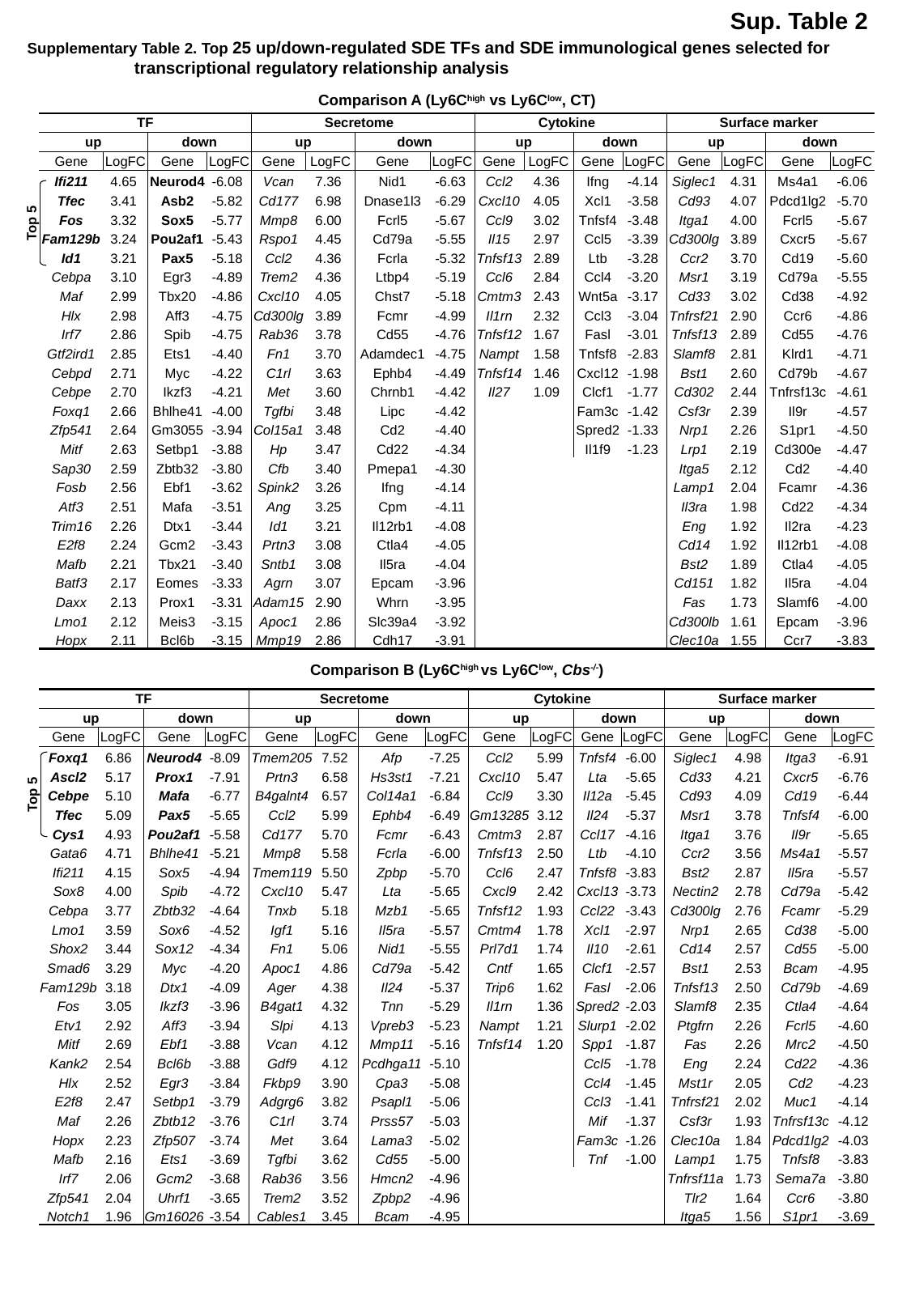

Sup. Table 2
Supplementary Table 2. Top 25 up/down-regulated SDE TFs and SDE immunological genes selected for
 transcriptional regulatory relationship analysis
Comparison A (Ly6Chigh vs Ly6Clow, CT)
| TF | | | | Secretome | | | | Cytokine | | | | Surface marker | | | |
| --- | --- | --- | --- | --- | --- | --- | --- | --- | --- | --- | --- | --- | --- | --- | --- |
| up | | down | | up | | down | | up | | down | | up | | down | |
| Gene | LogFC | Gene | LogFC | Gene | LogFC | Gene | LogFC | Gene | LogFC | Gene | LogFC | Gene | LogFC | Gene | LogFC |
| Ifi211 | 4.65 | Neurod4 | -6.08 | Vcan | 7.36 | Nid1 | -6.63 | Ccl2 | 4.36 | Ifng | -4.14 | Siglec1 | 4.31 | Ms4a1 | -6.06 |
| Tfec | 3.41 | Asb2 | -5.82 | Cd177 | 6.98 | Dnase1l3 | -6.29 | Cxcl10 | 4.05 | Xcl1 | -3.58 | Cd93 | 4.07 | Pdcd1lg2 | -5.70 |
| Fos | 3.32 | Sox5 | -5.77 | Mmp8 | 6.00 | Fcrl5 | -5.67 | Ccl9 | 3.02 | Tnfsf4 | -3.48 | Itga1 | 4.00 | Fcrl5 | -5.67 |
| Fam129b | 3.24 | Pou2af1 | -5.43 | Rspo1 | 4.45 | Cd79a | -5.55 | Il15 | 2.97 | Ccl5 | -3.39 | Cd300lg | 3.89 | Cxcr5 | -5.67 |
| Id1 | 3.21 | Pax5 | -5.18 | Ccl2 | 4.36 | Fcrla | -5.32 | Tnfsf13 | 2.89 | Ltb | -3.28 | Ccr2 | 3.70 | Cd19 | -5.60 |
| Cebpa | 3.10 | Egr3 | -4.89 | Trem2 | 4.36 | Ltbp4 | -5.19 | Ccl6 | 2.84 | Ccl4 | -3.20 | Msr1 | 3.19 | Cd79a | -5.55 |
| Maf | 2.99 | Tbx20 | -4.86 | Cxcl10 | 4.05 | Chst7 | -5.18 | Cmtm3 | 2.43 | Wnt5a | -3.17 | Cd33 | 3.02 | Cd38 | -4.92 |
| Hlx | 2.98 | Aff3 | -4.75 | Cd300lg | 3.89 | Fcmr | -4.99 | Il1rn | 2.32 | Ccl3 | -3.04 | Tnfrsf21 | 2.90 | Ccr6 | -4.86 |
| Irf7 | 2.86 | Spib | -4.75 | Rab36 | 3.78 | Cd55 | -4.76 | Tnfsf12 | 1.67 | Fasl | -3.01 | Tnfsf13 | 2.89 | Cd55 | -4.76 |
| Gtf2ird1 | 2.85 | Ets1 | -4.40 | Fn1 | 3.70 | Adamdec1 | -4.75 | Nampt | 1.58 | Tnfsf8 | -2.83 | Slamf8 | 2.81 | Klrd1 | -4.71 |
| Cebpd | 2.71 | Myc | -4.22 | C1rl | 3.63 | Ephb4 | -4.49 | Tnfsf14 | 1.46 | Cxcl12 | -1.98 | Bst1 | 2.60 | Cd79b | -4.67 |
| Cebpe | 2.70 | Ikzf3 | -4.21 | Met | 3.60 | Chrnb1 | -4.42 | Il27 | 1.09 | Clcf1 | -1.77 | Cd302 | 2.44 | Tnfrsf13c | -4.61 |
| Foxq1 | 2.66 | Bhlhe41 | -4.00 | Tgfbi | 3.48 | Lipc | -4.42 | | | Fam3c | -1.42 | Csf3r | 2.39 | Il9r | -4.57 |
| Zfp541 | 2.64 | Gm3055 | -3.94 | Col15a1 | 3.48 | Cd2 | -4.40 | | | Spred2 | -1.33 | Nrp1 | 2.26 | S1pr1 | -4.50 |
| Mitf | 2.63 | Setbp1 | -3.88 | Hp | 3.47 | Cd22 | -4.34 | | | Il1f9 | -1.23 | Lrp1 | 2.19 | Cd300e | -4.47 |
| Sap30 | 2.59 | Zbtb32 | -3.80 | Cfb | 3.40 | Pmepa1 | -4.30 | | | | | Itga5 | 2.12 | Cd2 | -4.40 |
| Fosb | 2.56 | Ebf1 | -3.62 | Spink2 | 3.26 | Ifng | -4.14 | | | | | Lamp1 | 2.04 | Fcamr | -4.36 |
| Atf3 | 2.51 | Mafa | -3.51 | Ang | 3.25 | Cpm | -4.11 | | | | | Il3ra | 1.98 | Cd22 | -4.34 |
| Trim16 | 2.26 | Dtx1 | -3.44 | Id1 | 3.21 | Il12rb1 | -4.08 | | | | | Eng | 1.92 | Il2ra | -4.23 |
| E2f8 | 2.24 | Gcm2 | -3.43 | Prtn3 | 3.08 | Ctla4 | -4.05 | | | | | Cd14 | 1.92 | Il12rb1 | -4.08 |
| Mafb | 2.21 | Tbx21 | -3.40 | Sntb1 | 3.08 | Il5ra | -4.04 | | | | | Bst2 | 1.89 | Ctla4 | -4.05 |
| Batf3 | 2.17 | Eomes | -3.33 | Agrn | 3.07 | Epcam | -3.96 | | | | | Cd151 | 1.82 | Il5ra | -4.04 |
| Daxx | 2.13 | Prox1 | -3.31 | Adam15 | 2.90 | Whrn | -3.95 | | | | | Fas | 1.73 | Slamf6 | -4.00 |
| Lmo1 | 2.12 | Meis3 | -3.15 | Apoc1 | 2.86 | Slc39a4 | -3.92 | | | | | Cd300lb | 1.61 | Epcam | -3.96 |
| Hopx | 2.11 | Bcl6b | -3.15 | Mmp19 | 2.86 | Cdh17 | -3.91 | | | | | Clec10a | 1.55 | Ccr7 | -3.83 |
Top 5
Comparison B (Ly6Chigh vs Ly6Clow, Cbs-/-)
| TF | | | | Secretome | | | | Cytokine | | | | Surface marker | | | |
| --- | --- | --- | --- | --- | --- | --- | --- | --- | --- | --- | --- | --- | --- | --- | --- |
| up | | down | | up | | down | | up | | down | | up | | down | |
| Gene | LogFC | Gene | LogFC | Gene | LogFC | Gene | LogFC | Gene | LogFC | Gene | LogFC | Gene | LogFC | Gene | LogFC |
| Foxq1 | 6.86 | Neurod4 | -8.09 | Tmem205 | 7.52 | Afp | -7.25 | Ccl2 | 5.99 | Tnfsf4 | -6.00 | Siglec1 | 4.98 | Itga3 | -6.91 |
| Ascl2 | 5.17 | Prox1 | -7.91 | Prtn3 | 6.58 | Hs3st1 | -7.21 | Cxcl10 | 5.47 | Lta | -5.65 | Cd33 | 4.21 | Cxcr5 | -6.76 |
| Cebpe | 5.10 | Mafa | -6.77 | B4galnt4 | 6.57 | Col14a1 | -6.84 | Ccl9 | 3.30 | Il12a | -5.45 | Cd93 | 4.09 | Cd19 | -6.44 |
| Tfec | 5.09 | Pax5 | -5.65 | Ccl2 | 5.99 | Ephb4 | -6.49 | Gm13285 | 3.12 | Il24 | -5.37 | Msr1 | 3.78 | Tnfsf4 | -6.00 |
| Cys1 | 4.93 | Pou2af1 | -5.58 | Cd177 | 5.70 | Fcmr | -6.43 | Cmtm3 | 2.87 | Ccl17 | -4.16 | Itga1 | 3.76 | Il9r | -5.65 |
| Gata6 | 4.71 | Bhlhe41 | -5.21 | Mmp8 | 5.58 | Fcrla | -6.00 | Tnfsf13 | 2.50 | Ltb | -4.10 | Ccr2 | 3.56 | Ms4a1 | -5.57 |
| Ifi211 | 4.15 | Sox5 | -4.94 | Tmem119 | 5.50 | Zpbp | -5.70 | Ccl6 | 2.47 | Tnfsf8 | -3.83 | Bst2 | 2.87 | Il5ra | -5.57 |
| Sox8 | 4.00 | Spib | -4.72 | Cxcl10 | 5.47 | Lta | -5.65 | Cxcl9 | 2.42 | Cxcl13 | -3.73 | Nectin2 | 2.78 | Cd79a | -5.42 |
| Cebpa | 3.77 | Zbtb32 | -4.64 | Tnxb | 5.18 | Mzb1 | -5.65 | Tnfsf12 | 1.93 | Ccl22 | -3.43 | Cd300lg | 2.76 | Fcamr | -5.29 |
| Lmo1 | 3.59 | Sox6 | -4.52 | Igf1 | 5.16 | Il5ra | -5.57 | Cmtm4 | 1.78 | Xcl1 | -2.97 | Nrp1 | 2.65 | Cd38 | -5.00 |
| Shox2 | 3.44 | Sox12 | -4.34 | Fn1 | 5.06 | Nid1 | -5.55 | Prl7d1 | 1.74 | Il10 | -2.61 | Cd14 | 2.57 | Cd55 | -5.00 |
| Smad6 | 3.29 | Myc | -4.20 | Apoc1 | 4.86 | Cd79a | -5.42 | Cntf | 1.65 | Clcf1 | -2.57 | Bst1 | 2.53 | Bcam | -4.95 |
| Fam129b | 3.18 | Dtx1 | -4.09 | Ager | 4.38 | Il24 | -5.37 | Trip6 | 1.62 | Fasl | -2.06 | Tnfsf13 | 2.50 | Cd79b | -4.69 |
| Fos | 3.05 | Ikzf3 | -3.96 | B4gat1 | 4.32 | Tnn | -5.29 | Il1rn | 1.36 | Spred2 | -2.03 | Slamf8 | 2.35 | Ctla4 | -4.64 |
| Etv1 | 2.92 | Aff3 | -3.94 | Slpi | 4.13 | Vpreb3 | -5.23 | Nampt | 1.21 | Slurp1 | -2.02 | Ptgfrn | 2.26 | Fcrl5 | -4.60 |
| Mitf | 2.69 | Ebf1 | -3.88 | Vcan | 4.12 | Mmp11 | -5.16 | Tnfsf14 | 1.20 | Spp1 | -1.87 | Fas | 2.26 | Mrc2 | -4.50 |
| Kank2 | 2.54 | Bcl6b | -3.88 | Gdf9 | 4.12 | Pcdhga11 | -5.10 | | | Ccl5 | -1.78 | Eng | 2.24 | Cd22 | -4.36 |
| Hlx | 2.52 | Egr3 | -3.84 | Fkbp9 | 3.90 | Cpa3 | -5.08 | | | Ccl4 | -1.45 | Mst1r | 2.05 | Cd2 | -4.23 |
| E2f8 | 2.47 | Setbp1 | -3.79 | Adgrg6 | 3.82 | Psapl1 | -5.06 | | | Ccl3 | -1.41 | Tnfrsf21 | 2.02 | Muc1 | -4.14 |
| Maf | 2.26 | Zbtb12 | -3.76 | C1rl | 3.74 | Prss57 | -5.03 | | | Mif | -1.37 | Csf3r | 1.93 | Tnfrsf13c | -4.12 |
| Hopx | 2.23 | Zfp507 | -3.74 | Met | 3.64 | Lama3 | -5.02 | | | Fam3c | -1.26 | Clec10a | 1.84 | Pdcd1lg2 | -4.03 |
| Mafb | 2.16 | Ets1 | -3.69 | Tgfbi | 3.62 | Cd55 | -5.00 | | | Tnf | -1.00 | Lamp1 | 1.75 | Tnfsf8 | -3.83 |
| Irf7 | 2.06 | Gcm2 | -3.68 | Rab36 | 3.56 | Hmcn2 | -4.96 | | | | | Tnfrsf11a | 1.73 | Sema7a | -3.80 |
| Zfp541 | 2.04 | Uhrf1 | -3.65 | Trem2 | 3.52 | Zpbp2 | -4.96 | | | | | Tlr2 | 1.64 | Ccr6 | -3.80 |
| Notch1 | 1.96 | Gm16026 | -3.54 | Cables1 | 3.45 | Bcam | -4.95 | | | | | Itga5 | 1.56 | S1pr1 | -3.69 |
Top 5

## Slide 4
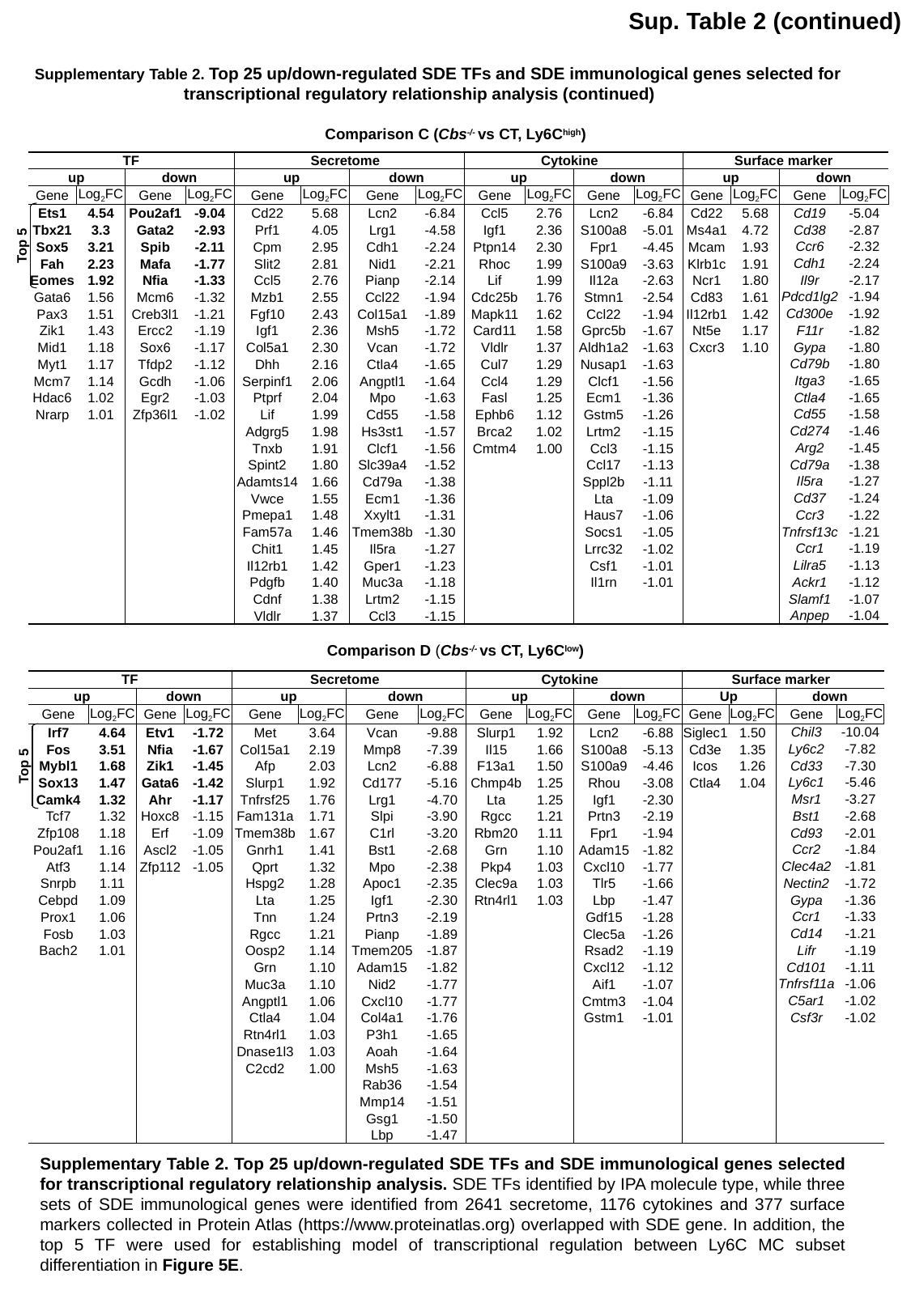

Sup. Table 2 (continued)
Supplementary Table 2. Top 25 up/down-regulated SDE TFs and SDE immunological genes selected for
 transcriptional regulatory relationship analysis (continued)
Comparison C (Cbs-/- vs CT, Ly6Chigh)
| TF | | | | Secretome | | | | Cytokine | | | | Surface marker | | | |
| --- | --- | --- | --- | --- | --- | --- | --- | --- | --- | --- | --- | --- | --- | --- | --- |
| up | | down | | up | | down | | up | | down | | up | | down | |
| Gene | Log2FC | Gene | Log2FC | Gene | Log2FC | Gene | Log2FC | Gene | Log2FC | Gene | Log2FC | Gene | Log2FC | Gene | Log2FC |
| Ets1 | 4.54 | Pou2af1 | -9.04 | Cd22 | 5.68 | Lcn2 | -6.84 | Ccl5 | 2.76 | Lcn2 | -6.84 | Cd22 | 5.68 | Cd19 | -5.04 |
| Tbx21 | 3.3 | Gata2 | -2.93 | Prf1 | 4.05 | Lrg1 | -4.58 | Igf1 | 2.36 | S100a8 | -5.01 | Ms4a1 | 4.72 | Cd38 | -2.87 |
| Sox5 | 3.21 | Spib | -2.11 | Cpm | 2.95 | Cdh1 | -2.24 | Ptpn14 | 2.30 | Fpr1 | -4.45 | Mcam | 1.93 | Ccr6 | -2.32 |
| Fah | 2.23 | Mafa | -1.77 | Slit2 | 2.81 | Nid1 | -2.21 | Rhoc | 1.99 | S100a9 | -3.63 | Klrb1c | 1.91 | Cdh1 | -2.24 |
| Eomes | 1.92 | Nfia | -1.33 | Ccl5 | 2.76 | Pianp | -2.14 | Lif | 1.99 | Il12a | -2.63 | Ncr1 | 1.80 | Il9r | -2.17 |
| Gata6 | 1.56 | Mcm6 | -1.32 | Mzb1 | 2.55 | Ccl22 | -1.94 | Cdc25b | 1.76 | Stmn1 | -2.54 | Cd83 | 1.61 | Pdcd1lg2 | -1.94 |
| Pax3 | 1.51 | Creb3l1 | -1.21 | Fgf10 | 2.43 | Col15a1 | -1.89 | Mapk11 | 1.62 | Ccl22 | -1.94 | Il12rb1 | 1.42 | Cd300e | -1.92 |
| Zik1 | 1.43 | Ercc2 | -1.19 | Igf1 | 2.36 | Msh5 | -1.72 | Card11 | 1.58 | Gprc5b | -1.67 | Nt5e | 1.17 | F11r | -1.82 |
| Mid1 | 1.18 | Sox6 | -1.17 | Col5a1 | 2.30 | Vcan | -1.72 | Vldlr | 1.37 | Aldh1a2 | -1.63 | Cxcr3 | 1.10 | Gypa | -1.80 |
| Myt1 | 1.17 | Tfdp2 | -1.12 | Dhh | 2.16 | Ctla4 | -1.65 | Cul7 | 1.29 | Nusap1 | -1.63 | | | Cd79b | -1.80 |
| Mcm7 | 1.14 | Gcdh | -1.06 | Serpinf1 | 2.06 | Angptl1 | -1.64 | Ccl4 | 1.29 | Clcf1 | -1.56 | | | Itga3 | -1.65 |
| Hdac6 | 1.02 | Egr2 | -1.03 | Ptprf | 2.04 | Mpo | -1.63 | Fasl | 1.25 | Ecm1 | -1.36 | | | Ctla4 | -1.65 |
| Nrarp | 1.01 | Zfp36l1 | -1.02 | Lif | 1.99 | Cd55 | -1.58 | Ephb6 | 1.12 | Gstm5 | -1.26 | | | Cd55 | -1.58 |
| | | | | Adgrg5 | 1.98 | Hs3st1 | -1.57 | Brca2 | 1.02 | Lrtm2 | -1.15 | | | Cd274 | -1.46 |
| | | | | Tnxb | 1.91 | Clcf1 | -1.56 | Cmtm4 | 1.00 | Ccl3 | -1.15 | | | Arg2 | -1.45 |
| | | | | Spint2 | 1.80 | Slc39a4 | -1.52 | | | Ccl17 | -1.13 | | | Cd79a | -1.38 |
| | | | | Adamts14 | 1.66 | Cd79a | -1.38 | | | Sppl2b | -1.11 | | | Il5ra | -1.27 |
| | | | | Vwce | 1.55 | Ecm1 | -1.36 | | | Lta | -1.09 | | | Cd37 | -1.24 |
| | | | | Pmepa1 | 1.48 | Xxylt1 | -1.31 | | | Haus7 | -1.06 | | | Ccr3 | -1.22 |
| | | | | Fam57a | 1.46 | Tmem38b | -1.30 | | | Socs1 | -1.05 | | | Tnfrsf13c | -1.21 |
| | | | | Chit1 | 1.45 | Il5ra | -1.27 | | | Lrrc32 | -1.02 | | | Ccr1 | -1.19 |
| | | | | Il12rb1 | 1.42 | Gper1 | -1.23 | | | Csf1 | -1.01 | | | Lilra5 | -1.13 |
| | | | | Pdgfb | 1.40 | Muc3a | -1.18 | | | Il1rn | -1.01 | | | Ackr1 | -1.12 |
| | | | | Cdnf | 1.38 | Lrtm2 | -1.15 | | | | | | | Slamf1 | -1.07 |
| | | | | Vldlr | 1.37 | Ccl3 | -1.15 | | | | | | | Anpep | -1.04 |
Top 5
Comparison D (Cbs-/- vs CT, Ly6Clow)
| TF | | | | Secretome | | | | Cytokine | | | | Surface marker | | | |
| --- | --- | --- | --- | --- | --- | --- | --- | --- | --- | --- | --- | --- | --- | --- | --- |
| up | | down | | up | | down | | up | | down | | Up | | down | |
| Gene | Log2FC | Gene | Log2FC | Gene | Log2FC | Gene | Log2FC | Gene | Log2FC | Gene | Log2FC | Gene | Log2FC | Gene | Log2FC |
| Irf7 | 4.64 | Etv1 | -1.72 | Met | 3.64 | Vcan | -9.88 | Slurp1 | 1.92 | Lcn2 | -6.88 | Siglec1 | 1.50 | Chil3 | -10.04 |
| Fos | 3.51 | Nfia | -1.67 | Col15a1 | 2.19 | Mmp8 | -7.39 | Il15 | 1.66 | S100a8 | -5.13 | Cd3e | 1.35 | Ly6c2 | -7.82 |
| Mybl1 | 1.68 | Zik1 | -1.45 | Afp | 2.03 | Lcn2 | -6.88 | F13a1 | 1.50 | S100a9 | -4.46 | Icos | 1.26 | Cd33 | -7.30 |
| Sox13 | 1.47 | Gata6 | -1.42 | Slurp1 | 1.92 | Cd177 | -5.16 | Chmp4b | 1.25 | Rhou | -3.08 | Ctla4 | 1.04 | Ly6c1 | -5.46 |
| Camk4 | 1.32 | Ahr | -1.17 | Tnfrsf25 | 1.76 | Lrg1 | -4.70 | Lta | 1.25 | Igf1 | -2.30 | | | Msr1 | -3.27 |
| Tcf7 | 1.32 | Hoxc8 | -1.15 | Fam131a | 1.71 | Slpi | -3.90 | Rgcc | 1.21 | Prtn3 | -2.19 | | | Bst1 | -2.68 |
| Zfp108 | 1.18 | Erf | -1.09 | Tmem38b | 1.67 | C1rl | -3.20 | Rbm20 | 1.11 | Fpr1 | -1.94 | | | Cd93 | -2.01 |
| Pou2af1 | 1.16 | Ascl2 | -1.05 | Gnrh1 | 1.41 | Bst1 | -2.68 | Grn | 1.10 | Adam15 | -1.82 | | | Ccr2 | -1.84 |
| Atf3 | 1.14 | Zfp112 | -1.05 | Qprt | 1.32 | Mpo | -2.38 | Pkp4 | 1.03 | Cxcl10 | -1.77 | | | Clec4a2 | -1.81 |
| Snrpb | 1.11 | | | Hspg2 | 1.28 | Apoc1 | -2.35 | Clec9a | 1.03 | Tlr5 | -1.66 | | | Nectin2 | -1.72 |
| Cebpd | 1.09 | | | Lta | 1.25 | Igf1 | -2.30 | Rtn4rl1 | 1.03 | Lbp | -1.47 | | | Gypa | -1.36 |
| Prox1 | 1.06 | | | Tnn | 1.24 | Prtn3 | -2.19 | | | Gdf15 | -1.28 | | | Ccr1 | -1.33 |
| Fosb | 1.03 | | | Rgcc | 1.21 | Pianp | -1.89 | | | Clec5a | -1.26 | | | Cd14 | -1.21 |
| Bach2 | 1.01 | | | Oosp2 | 1.14 | Tmem205 | -1.87 | | | Rsad2 | -1.19 | | | Lifr | -1.19 |
| | | | | Grn | 1.10 | Adam15 | -1.82 | | | Cxcl12 | -1.12 | | | Cd101 | -1.11 |
| | | | | Muc3a | 1.10 | Nid2 | -1.77 | | | Aif1 | -1.07 | | | Tnfrsf11a | -1.06 |
| | | | | Angptl1 | 1.06 | Cxcl10 | -1.77 | | | Cmtm3 | -1.04 | | | C5ar1 | -1.02 |
| | | | | Ctla4 | 1.04 | Col4a1 | -1.76 | | | Gstm1 | -1.01 | | | Csf3r | -1.02 |
| | | | | Rtn4rl1 | 1.03 | P3h1 | -1.65 | | | | | | | | |
| | | | | Dnase1l3 | 1.03 | Aoah | -1.64 | | | | | | | | |
| | | | | C2cd2 | 1.00 | Msh5 | -1.63 | | | | | | | | |
| | | | | | | Rab36 | -1.54 | | | | | | | | |
| | | | | | | Mmp14 | -1.51 | | | | | | | | |
| | | | | | | Gsg1 | -1.50 | | | | | | | | |
| | | | | | | Lbp | -1.47 | | | | | | | | |
Top 5
Supplementary Table 2. Top 25 up/down-regulated SDE TFs and SDE immunological genes selected for transcriptional regulatory relationship analysis. SDE TFs identified by IPA molecule type, while three sets of SDE immunological genes were identified from 2641 secretome, 1176 cytokines and 377 surface markers collected in Protein Atlas (https://www.proteinatlas.org) overlapped with SDE gene. In addition, the top 5 TF were used for establishing model of transcriptional regulation between Ly6C MC subset differentiation in Figure 5E.

## Slide 5
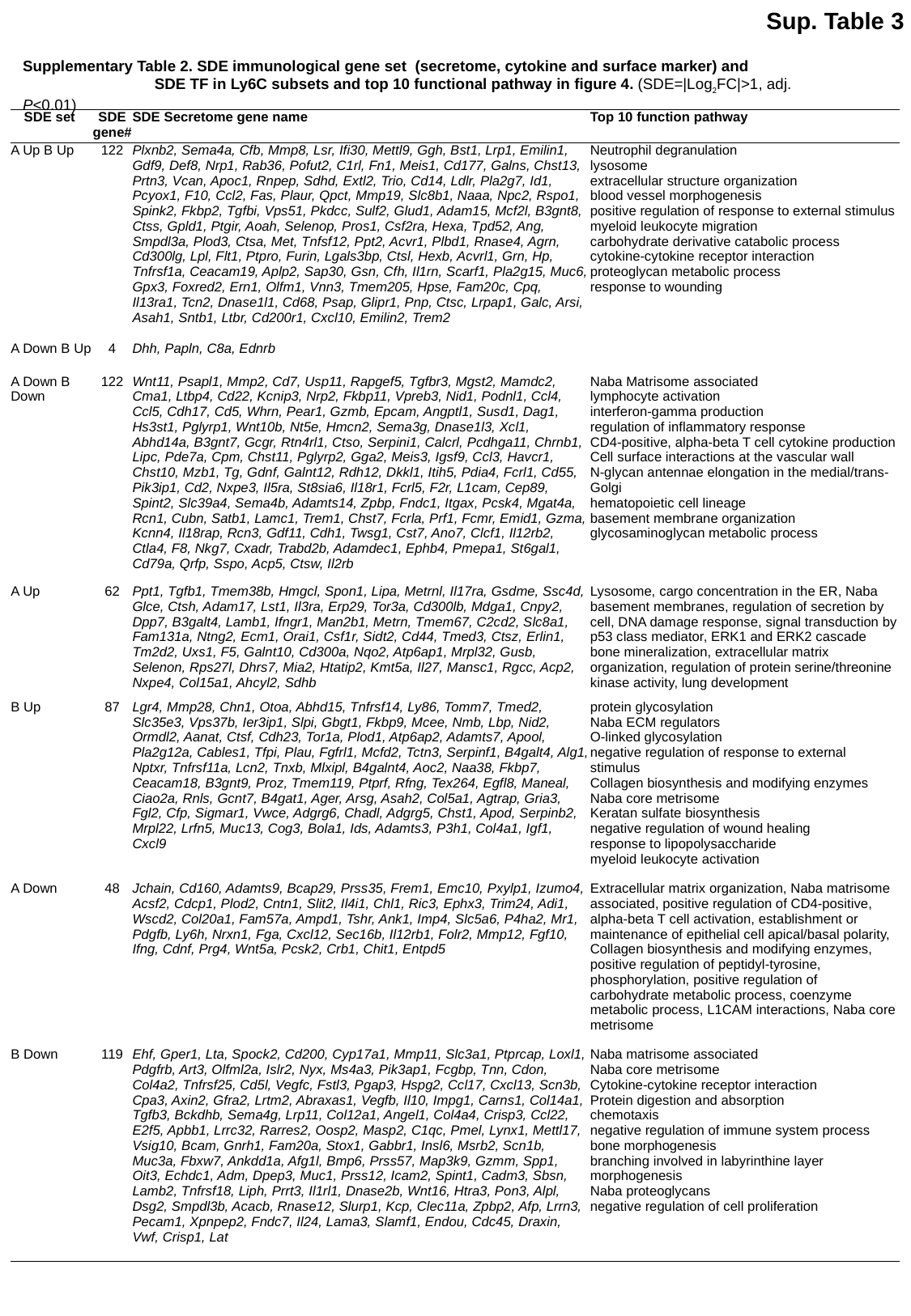

Sup. Table 3
Supplementary Table 2. SDE immunological gene set (secretome, cytokine and surface marker) and
 SDE TF in Ly6C subsets and top 10 functional pathway in figure 4. (SDE=|Log2FC|>1, adj. P<0.01)
| SDE set | SDE gene# | SDE Secretome gene name | Top 10 function pathway |
| --- | --- | --- | --- |
| A Up B Up | 122 | Plxnb2, Sema4a, Cfb, Mmp8, Lsr, Ifi30, Mettl9, Ggh, Bst1, Lrp1, Emilin1, Gdf9, Def8, Nrp1, Rab36, Pofut2, C1rl, Fn1, Meis1, Cd177, Galns, Chst13, Prtn3, Vcan, Apoc1, Rnpep, Sdhd, Extl2, Trio, Cd14, Ldlr, Pla2g7, Id1, Pcyox1, F10, Ccl2, Fas, Plaur, Qpct, Mmp19, Slc8b1, Naaa, Npc2, Rspo1, Spink2, Fkbp2, Tgfbi, Vps51, Pkdcc, Sulf2, Glud1, Adam15, Mcf2l, B3gnt8, Ctss, Gpld1, Ptgir, Aoah, Selenop, Pros1, Csf2ra, Hexa, Tpd52, Ang, Smpdl3a, Plod3, Ctsa, Met, Tnfsf12, Ppt2, Acvr1, Plbd1, Rnase4, Agrn, Cd300lg, Lpl, Flt1, Ptpro, Furin, Lgals3bp, Ctsl, Hexb, Acvrl1, Grn, Hp, Tnfrsf1a, Ceacam19, Aplp2, Sap30, Gsn, Cfh, Il1rn, Scarf1, Pla2g15, Muc6, Gpx3, Foxred2, Ern1, Olfm1, Vnn3, Tmem205, Hpse, Fam20c, Cpq, Il13ra1, Tcn2, Dnase1l1, Cd68, Psap, Glipr1, Pnp, Ctsc, Lrpap1, Galc, Arsi, Asah1, Sntb1, Ltbr, Cd200r1, Cxcl10, Emilin2, Trem2 | Neutrophil degranulation lysosome extracellular structure organization blood vessel morphogenesis positive regulation of response to external stimulus myeloid leukocyte migration carbohydrate derivative catabolic process cytokine-cytokine receptor interaction proteoglycan metabolic process response to wounding |
| A Down B Up | 4 | Dhh, Papln, C8a, Ednrb | |
| A Down B Down | 122 | Wnt11, Psapl1, Mmp2, Cd7, Usp11, Rapgef5, Tgfbr3, Mgst2, Mamdc2, Cma1, Ltbp4, Cd22, Kcnip3, Nrp2, Fkbp11, Vpreb3, Nid1, Podnl1, Ccl4, Ccl5, Cdh17, Cd5, Whrn, Pear1, Gzmb, Epcam, Angptl1, Susd1, Dag1, Hs3st1, Pglyrp1, Wnt10b, Nt5e, Hmcn2, Sema3g, Dnase1l3, Xcl1, Abhd14a, B3gnt7, Gcgr, Rtn4rl1, Ctso, Serpini1, Calcrl, Pcdhga11, Chrnb1, Lipc, Pde7a, Cpm, Chst11, Pglyrp2, Gga2, Meis3, Igsf9, Ccl3, Havcr1, Chst10, Mzb1, Tg, Gdnf, Galnt12, Rdh12, Dkkl1, Itih5, Pdia4, Fcrl1, Cd55, Pik3ip1, Cd2, Nxpe3, Il5ra, St8sia6, Il18r1, Fcrl5, F2r, L1cam, Cep89, Spint2, Slc39a4, Sema4b, Adamts14, Zpbp, Fndc1, Itgax, Pcsk4, Mgat4a, Rcn1, Cubn, Satb1, Lamc1, Trem1, Chst7, Fcrla, Prf1, Fcmr, Emid1, Gzma, Kcnn4, Il18rap, Rcn3, Gdf11, Cdh1, Twsg1, Cst7, Ano7, Clcf1, Il12rb2, Ctla4, F8, Nkg7, Cxadr, Trabd2b, Adamdec1, Ephb4, Pmepa1, St6gal1, Cd79a, Qrfp, Sspo, Acp5, Ctsw, Il2rb | Naba Matrisome associated lymphocyte activation interferon-gamma production regulation of inflammatory response CD4-positive, alpha-beta T cell cytokine production Cell surface interactions at the vascular wall N-glycan antennae elongation in the medial/trans-Golgi hematopoietic cell lineage basement membrane organization glycosaminoglycan metabolic process |
| A Up | 62 | Ppt1, Tgfb1, Tmem38b, Hmgcl, Spon1, Lipa, Metrnl, Il17ra, Gsdme, Ssc4d, Glce, Ctsh, Adam17, Lst1, Il3ra, Erp29, Tor3a, Cd300lb, Mdga1, Cnpy2, Dpp7, B3galt4, Lamb1, Ifngr1, Man2b1, Metrn, Tmem67, C2cd2, Slc8a1, Fam131a, Ntng2, Ecm1, Orai1, Csf1r, Sidt2, Cd44, Tmed3, Ctsz, Erlin1, Tm2d2, Uxs1, F5, Galnt10, Cd300a, Nqo2, Atp6ap1, Mrpl32, Gusb, Selenon, Rps27l, Dhrs7, Mia2, Htatip2, Kmt5a, Il27, Mansc1, Rgcc, Acp2, Nxpe4, Col15a1, Ahcyl2, Sdhb | Lysosome, cargo concentration in the ER, Naba basement membranes, regulation of secretion by cell, DNA damage response, signal transduction by p53 class mediator, ERK1 and ERK2 cascade bone mineralization, extracellular matrix organization, regulation of protein serine/threonine kinase activity, lung development |
| B Up | 87 | Lgr4, Mmp28, Chn1, Otoa, Abhd15, Tnfrsf14, Ly86, Tomm7, Tmed2, Slc35e3, Vps37b, Ier3ip1, Slpi, Gbgt1, Fkbp9, Mcee, Nmb, Lbp, Nid2, Ormdl2, Aanat, Ctsf, Cdh23, Tor1a, Plod1, Atp6ap2, Adamts7, Apool, Pla2g12a, Cables1, Tfpi, Plau, Fgfrl1, Mcfd2, Tctn3, Serpinf1, B4galt4, Alg1, Nptxr, Tnfrsf11a, Lcn2, Tnxb, Mlxipl, B4galnt4, Aoc2, Naa38, Fkbp7, Ceacam18, B3gnt9, Proz, Tmem119, Ptprf, Rfng, Tex264, Egfl8, Maneal, Ciao2a, Rnls, Gcnt7, B4gat1, Ager, Arsg, Asah2, Col5a1, Agtrap, Gria3, Fgl2, Cfp, Sigmar1, Vwce, Adgrg6, Chadl, Adgrg5, Chst1, Apod, Serpinb2, Mrpl22, Lrfn5, Muc13, Cog3, Bola1, Ids, Adamts3, P3h1, Col4a1, Igf1, Cxcl9 | protein glycosylation Naba ECM regulators O-linked glycosylation negative regulation of response to external stimulus Collagen biosynthesis and modifying enzymes Naba core metrisome Keratan sulfate biosynthesis negative regulation of wound healing response to lipopolysaccharide myeloid leukocyte activation |
| A Down | 48 | Jchain, Cd160, Adamts9, Bcap29, Prss35, Frem1, Emc10, Pxylp1, Izumo4, Acsf2, Cdcp1, Plod2, Cntn1, Slit2, Il4i1, Chl1, Ric3, Ephx3, Trim24, Adi1, Wscd2, Col20a1, Fam57a, Ampd1, Tshr, Ank1, Imp4, Slc5a6, P4ha2, Mr1, Pdgfb, Ly6h, Nrxn1, Fga, Cxcl12, Sec16b, Il12rb1, Folr2, Mmp12, Fgf10, Ifng, Cdnf, Prg4, Wnt5a, Pcsk2, Crb1, Chit1, Entpd5 | Extracellular matrix organization, Naba matrisome associated, positive regulation of CD4-positive, alpha-beta T cell activation, establishment or maintenance of epithelial cell apical/basal polarity, Collagen biosynthesis and modifying enzymes, positive regulation of peptidyl-tyrosine, phosphorylation, positive regulation of carbohydrate metabolic process, coenzyme metabolic process, L1CAM interactions, Naba core metrisome |
| B Down | 119 | Ehf, Gper1, Lta, Spock2, Cd200, Cyp17a1, Mmp11, Slc3a1, Ptprcap, Loxl1, Pdgfrb, Art3, Olfml2a, Islr2, Nyx, Ms4a3, Pik3ap1, Fcgbp, Tnn, Cdon, Col4a2, Tnfrsf25, Cd5l, Vegfc, Fstl3, Pgap3, Hspg2, Ccl17, Cxcl13, Scn3b, Cpa3, Axin2, Gfra2, Lrtm2, Abraxas1, Vegfb, Il10, Impg1, Carns1, Col14a1, Tgfb3, Bckdhb, Sema4g, Lrp11, Col12a1, Angel1, Col4a4, Crisp3, Ccl22, E2f5, Apbb1, Lrrc32, Rarres2, Oosp2, Masp2, C1qc, Pmel, Lynx1, Mettl17, Vsig10, Bcam, Gnrh1, Fam20a, Stox1, Gabbr1, Insl6, Msrb2, Scn1b, Muc3a, Fbxw7, Ankdd1a, Afg1l, Bmp6, Prss57, Map3k9, Gzmm, Spp1, Oit3, Echdc1, Adm, Dpep3, Muc1, Prss12, Icam2, Spint1, Cadm3, Sbsn, Lamb2, Tnfrsf18, Liph, Prrt3, Il1rl1, Dnase2b, Wnt16, Htra3, Pon3, Alpl, Dsg2, Smpdl3b, Acacb, Rnase12, Slurp1, Kcp, Clec11a, Zpbp2, Afp, Lrrn3, Pecam1, Xpnpep2, Fndc7, Il24, Lama3, Slamf1, Endou, Cdc45, Draxin, Vwf, Crisp1, Lat | Naba matrisome associated Naba core metrisome Cytokine-cytokine receptor interaction Protein digestion and absorption chemotaxis negative regulation of immune system process bone morphogenesis branching involved in labyrinthine layer morphogenesis Naba proteoglycans negative regulation of cell proliferation |

## Slide 6
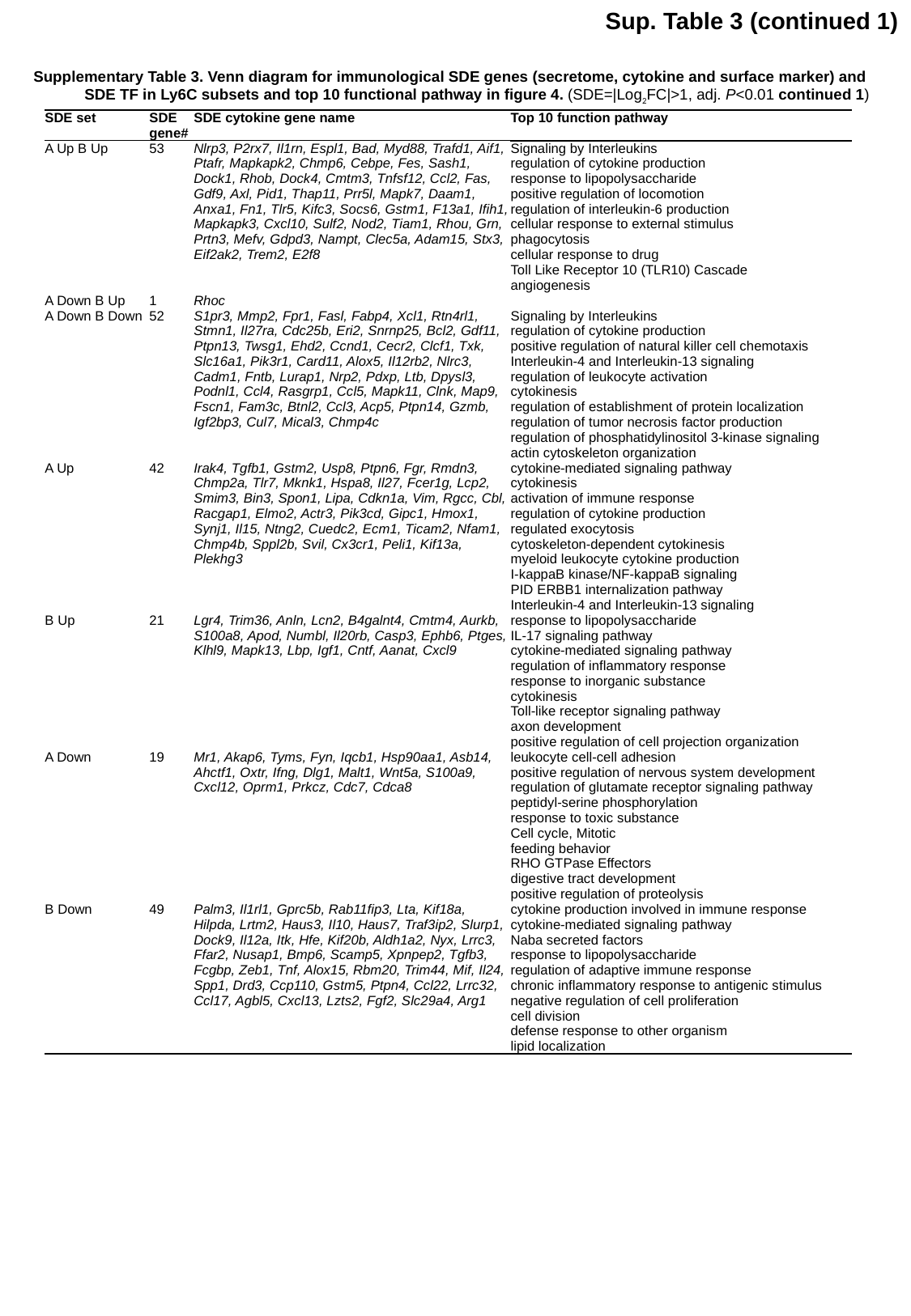

Sup. Table 3 (continued 1)
Supplementary Table 3. Venn diagram for immunological SDE genes (secretome, cytokine and surface marker) and
 SDE TF in Ly6C subsets and top 10 functional pathway in figure 4. (SDE=|Log2FC|>1, adj. P<0.01 continued 1)
| SDE set | SDE gene# | SDE cytokine gene name | Top 10 function pathway |
| --- | --- | --- | --- |
| A Up B Up | 53 | Nlrp3, P2rx7, Il1rn, Espl1, Bad, Myd88, Trafd1, Aif1, Ptafr, Mapkapk2, Chmp6, Cebpe, Fes, Sash1, Dock1, Rhob, Dock4, Cmtm3, Tnfsf12, Ccl2, Fas, Gdf9, Axl, Pid1, Thap11, Prr5l, Mapk7, Daam1, Anxa1, Fn1, Tlr5, Kifc3, Socs6, Gstm1, F13a1, Ifih1, Mapkapk3, Cxcl10, Sulf2, Nod2, Tiam1, Rhou, Grn, Prtn3, Mefv, Gdpd3, Nampt, Clec5a, Adam15, Stx3, Eif2ak2, Trem2, E2f8 | Signaling by Interleukins regulation of cytokine production response to lipopolysaccharide positive regulation of locomotion regulation of interleukin-6 production cellular response to external stimulus phagocytosis cellular response to drug Toll Like Receptor 10 (TLR10) Cascade angiogenesis |
| A Down B Up | 1 | Rhoc | |
| A Down B Down | 52 | S1pr3, Mmp2, Fpr1, Fasl, Fabp4, Xcl1, Rtn4rl1, Stmn1, Il27ra, Cdc25b, Eri2, Snrnp25, Bcl2, Gdf11, Ptpn13, Twsg1, Ehd2, Ccnd1, Cecr2, Clcf1, Txk, Slc16a1, Pik3r1, Card11, Alox5, Il12rb2, Nlrc3, Cadm1, Fntb, Lurap1, Nrp2, Pdxp, Ltb, Dpysl3, Podnl1, Ccl4, Rasgrp1, Ccl5, Mapk11, Clnk, Map9, Fscn1, Fam3c, Btnl2, Ccl3, Acp5, Ptpn14, Gzmb, Igf2bp3, Cul7, Mical3, Chmp4c | Signaling by Interleukins regulation of cytokine production positive regulation of natural killer cell chemotaxis Interleukin-4 and Interleukin-13 signaling regulation of leukocyte activation cytokinesis regulation of establishment of protein localization regulation of tumor necrosis factor production regulation of phosphatidylinositol 3-kinase signaling actin cytoskeleton organization |
| A Up | 42 | Irak4, Tgfb1, Gstm2, Usp8, Ptpn6, Fgr, Rmdn3, Chmp2a, Tlr7, Mknk1, Hspa8, Il27, Fcer1g, Lcp2, Smim3, Bin3, Spon1, Lipa, Cdkn1a, Vim, Rgcc, Cbl, Racgap1, Elmo2, Actr3, Pik3cd, Gipc1, Hmox1, Synj1, Il15, Ntng2, Cuedc2, Ecm1, Ticam2, Nfam1, Chmp4b, Sppl2b, Svil, Cx3cr1, Peli1, Kif13a, Plekhg3 | cytokine-mediated signaling pathway cytokinesis activation of immune response regulation of cytokine production regulated exocytosis cytoskeleton-dependent cytokinesis myeloid leukocyte cytokine production I-kappaB kinase/NF-kappaB signaling PID ERBB1 internalization pathway Interleukin-4 and Interleukin-13 signaling |
| B Up | 21 | Lgr4, Trim36, Anln, Lcn2, B4galnt4, Cmtm4, Aurkb, S100a8, Apod, Numbl, Il20rb, Casp3, Ephb6, Ptges, Klhl9, Mapk13, Lbp, Igf1, Cntf, Aanat, Cxcl9 | response to lipopolysaccharide IL-17 signaling pathway cytokine-mediated signaling pathway regulation of inflammatory response response to inorganic substance cytokinesis Toll-like receptor signaling pathway axon development positive regulation of cell projection organization |
| A Down | 19 | Mr1, Akap6, Tyms, Fyn, Iqcb1, Hsp90aa1, Asb14, Ahctf1, Oxtr, Ifng, Dlg1, Malt1, Wnt5a, S100a9, Cxcl12, Oprm1, Prkcz, Cdc7, Cdca8 | leukocyte cell-cell adhesion positive regulation of nervous system development regulation of glutamate receptor signaling pathway peptidyl-serine phosphorylation response to toxic substance Cell cycle, Mitotic feeding behavior RHO GTPase Effectors digestive tract development positive regulation of proteolysis |
| B Down | 49 | Palm3, Il1rl1, Gprc5b, Rab11fip3, Lta, Kif18a, Hilpda, Lrtm2, Haus3, Il10, Haus7, Traf3ip2, Slurp1, Dock9, Il12a, Itk, Hfe, Kif20b, Aldh1a2, Nyx, Lrrc3, Ffar2, Nusap1, Bmp6, Scamp5, Xpnpep2, Tgfb3, Fcgbp, Zeb1, Tnf, Alox15, Rbm20, Trim44, Mif, Il24, Spp1, Drd3, Ccp110, Gstm5, Ptpn4, Ccl22, Lrrc32, Ccl17, Agbl5, Cxcl13, Lzts2, Fgf2, Slc29a4, Arg1 | cytokine production involved in immune response cytokine-mediated signaling pathway Naba secreted factors response to lipopolysaccharide regulation of adaptive immune response chronic inflammatory response to antigenic stimulus negative regulation of cell proliferation cell division defense response to other organism lipid localization |

## Slide 7
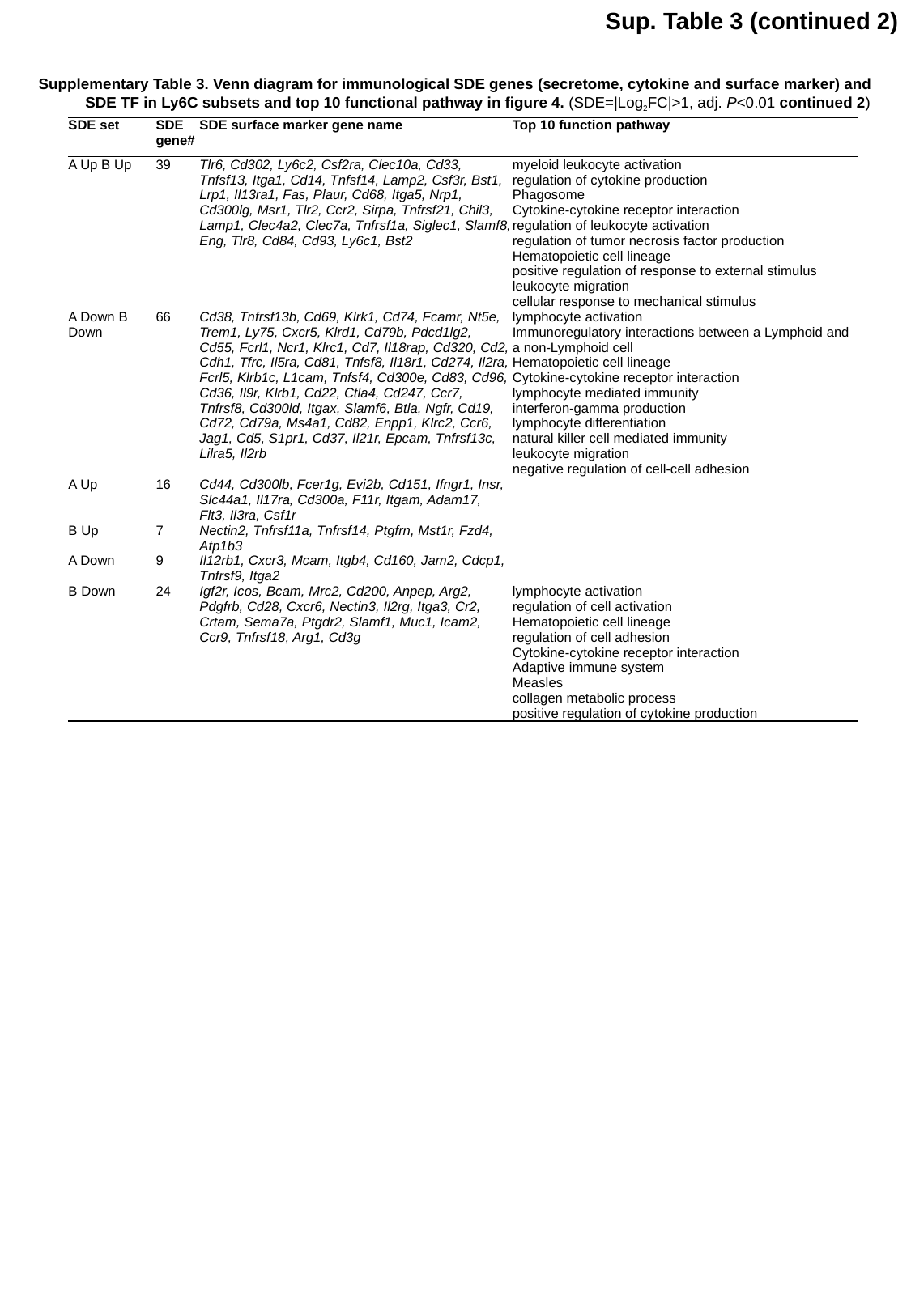

Sup. Table 3 (continued 2)
Supplementary Table 3. Venn diagram for immunological SDE genes (secretome, cytokine and surface marker) and
 SDE TF in Ly6C subsets and top 10 functional pathway in figure 4. (SDE=|Log2FC|>1, adj. P<0.01 continued 2)
| SDE set | SDE gene# | SDE surface marker gene name | Top 10 function pathway |
| --- | --- | --- | --- |
| A Up B Up | 39 | Tlr6, Cd302, Ly6c2, Csf2ra, Clec10a, Cd33, Tnfsf13, Itga1, Cd14, Tnfsf14, Lamp2, Csf3r, Bst1, Lrp1, Il13ra1, Fas, Plaur, Cd68, Itga5, Nrp1, Cd300lg, Msr1, Tlr2, Ccr2, Sirpa, Tnfrsf21, Chil3, Lamp1, Clec4a2, Clec7a, Tnfrsf1a, Siglec1, Slamf8, Eng, Tlr8, Cd84, Cd93, Ly6c1, Bst2 | myeloid leukocyte activation regulation of cytokine production Phagosome Cytokine-cytokine receptor interaction regulation of leukocyte activation regulation of tumor necrosis factor production Hematopoietic cell lineage positive regulation of response to external stimulus leukocyte migration cellular response to mechanical stimulus |
| A Down B Down | 66 | Cd38, Tnfrsf13b, Cd69, Klrk1, Cd74, Fcamr, Nt5e, Trem1, Ly75, Cxcr5, Klrd1, Cd79b, Pdcd1lg2, Cd55, Fcrl1, Ncr1, Klrc1, Cd7, Il18rap, Cd320, Cd2, Cdh1, Tfrc, Il5ra, Cd81, Tnfsf8, Il18r1, Cd274, Il2ra, Fcrl5, Klrb1c, L1cam, Tnfsf4, Cd300e, Cd83, Cd96, Cd36, Il9r, Klrb1, Cd22, Ctla4, Cd247, Ccr7, Tnfrsf8, Cd300ld, Itgax, Slamf6, Btla, Ngfr, Cd19, Cd72, Cd79a, Ms4a1, Cd82, Enpp1, Klrc2, Ccr6, Jag1, Cd5, S1pr1, Cd37, Il21r, Epcam, Tnfrsf13c, Lilra5, Il2rb | lymphocyte activation Immunoregulatory interactions between a Lymphoid and a non-Lymphoid cell Hematopoietic cell lineage Cytokine-cytokine receptor interaction lymphocyte mediated immunity interferon-gamma production lymphocyte differentiation natural killer cell mediated immunity leukocyte migration negative regulation of cell-cell adhesion |
| A Up | 16 | Cd44, Cd300lb, Fcer1g, Evi2b, Cd151, Ifngr1, Insr, Slc44a1, Il17ra, Cd300a, F11r, Itgam, Adam17, Flt3, Il3ra, Csf1r | |
| B Up | 7 | Nectin2, Tnfrsf11a, Tnfrsf14, Ptgfrn, Mst1r, Fzd4, Atp1b3 | |
| A Down | 9 | Il12rb1, Cxcr3, Mcam, Itgb4, Cd160, Jam2, Cdcp1, Tnfrsf9, Itga2 | |
| B Down | 24 | Igf2r, Icos, Bcam, Mrc2, Cd200, Anpep, Arg2, Pdgfrb, Cd28, Cxcr6, Nectin3, Il2rg, Itga3, Cr2, Crtam, Sema7a, Ptgdr2, Slamf1, Muc1, Icam2, Ccr9, Tnfrsf18, Arg1, Cd3g | lymphocyte activation regulation of cell activation Hematopoietic cell lineage regulation of cell adhesion Cytokine-cytokine receptor interaction Adaptive immune system Measles collagen metabolic process positive regulation of cytokine production |

## Slide 8
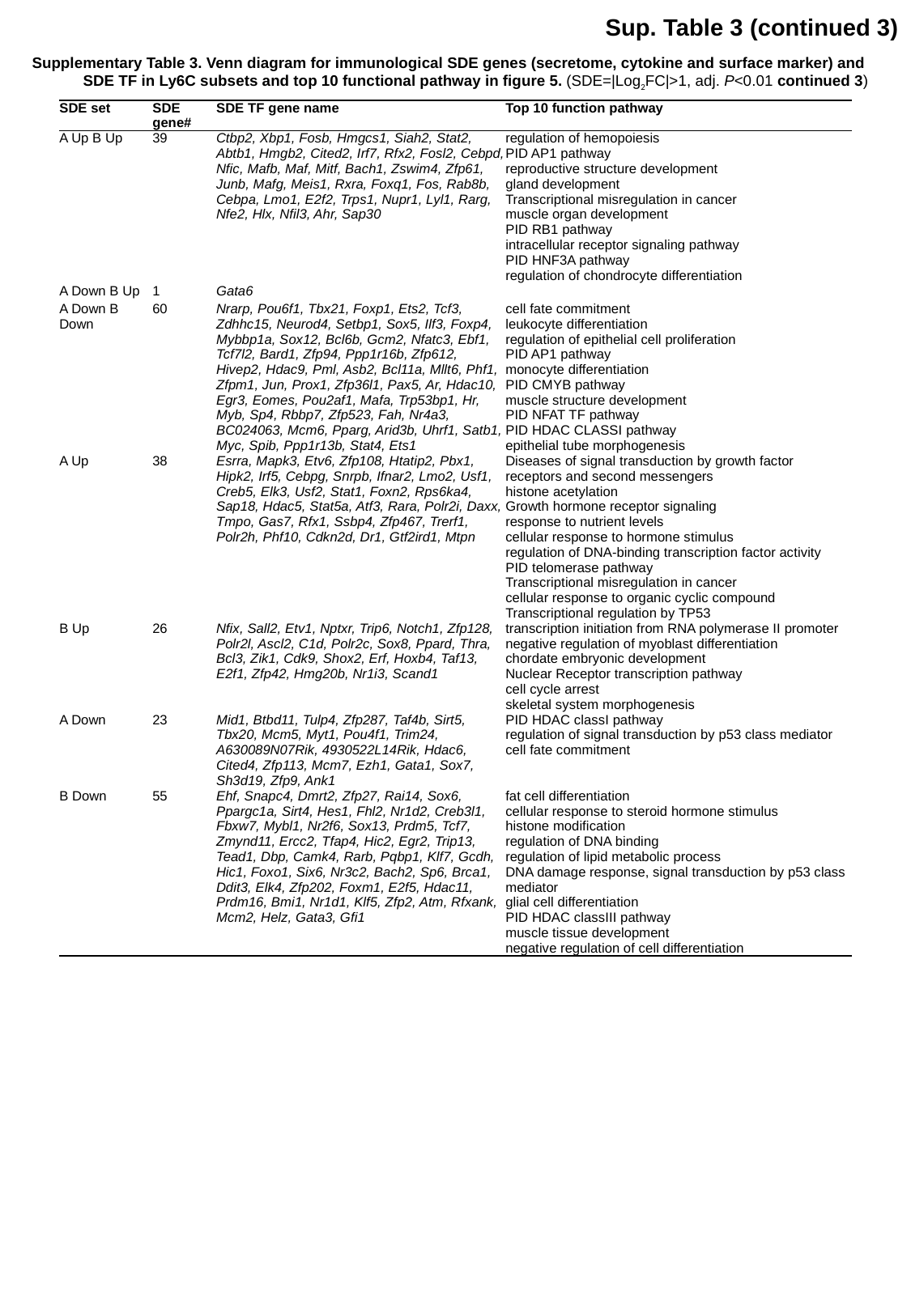

Sup. Table 3 (continued 3)
Supplementary Table 3. Venn diagram for immunological SDE genes (secretome, cytokine and surface marker) and
 SDE TF in Ly6C subsets and top 10 functional pathway in figure 5. (SDE=|Log2FC|>1, adj. P<0.01 continued 3)
| SDE set | SDE gene# | SDE TF gene name | Top 10 function pathway |
| --- | --- | --- | --- |
| A Up B Up | 39 | Ctbp2, Xbp1, Fosb, Hmgcs1, Siah2, Stat2, Abtb1, Hmgb2, Cited2, Irf7, Rfx2, Fosl2, Cebpd, Nfic, Mafb, Maf, Mitf, Bach1, Zswim4, Zfp61, Junb, Mafg, Meis1, Rxra, Foxq1, Fos, Rab8b, Cebpa, Lmo1, E2f2, Trps1, Nupr1, Lyl1, Rarg, Nfe2, Hlx, Nfil3, Ahr, Sap30 | regulation of hemopoiesis PID AP1 pathway reproductive structure development gland development Transcriptional misregulation in cancer muscle organ development PID RB1 pathway intracellular receptor signaling pathway PID HNF3A pathway regulation of chondrocyte differentiation |
| A Down B Up | 1 | Gata6 | |
| A Down B Down | 60 | Nrarp, Pou6f1, Tbx21, Foxp1, Ets2, Tcf3, Zdhhc15, Neurod4, Setbp1, Sox5, Ilf3, Foxp4, Mybbp1a, Sox12, Bcl6b, Gcm2, Nfatc3, Ebf1, Tcf7l2, Bard1, Zfp94, Ppp1r16b, Zfp612, Hivep2, Hdac9, Pml, Asb2, Bcl11a, Mllt6, Phf1, Zfpm1, Jun, Prox1, Zfp36l1, Pax5, Ar, Hdac10, Egr3, Eomes, Pou2af1, Mafa, Trp53bp1, Hr, Myb, Sp4, Rbbp7, Zfp523, Fah, Nr4a3, BC024063, Mcm6, Pparg, Arid3b, Uhrf1, Satb1, Myc, Spib, Ppp1r13b, Stat4, Ets1 | cell fate commitment leukocyte differentiation regulation of epithelial cell proliferation PID AP1 pathway monocyte differentiation PID CMYB pathway muscle structure development PID NFAT TF pathway PID HDAC CLASSI pathway epithelial tube morphogenesis |
| A Up | 38 | Esrra, Mapk3, Etv6, Zfp108, Htatip2, Pbx1, Hipk2, Irf5, Cebpg, Snrpb, Ifnar2, Lmo2, Usf1, Creb5, Elk3, Usf2, Stat1, Foxn2, Rps6ka4, Sap18, Hdac5, Stat5a, Atf3, Rara, Polr2i, Daxx, Tmpo, Gas7, Rfx1, Ssbp4, Zfp467, Trerf1, Polr2h, Phf10, Cdkn2d, Dr1, Gtf2ird1, Mtpn | Diseases of signal transduction by growth factor receptors and second messengers histone acetylation Growth hormone receptor signaling response to nutrient levels cellular response to hormone stimulus regulation of DNA-binding transcription factor activity PID telomerase pathway Transcriptional misregulation in cancer cellular response to organic cyclic compound Transcriptional regulation by TP53 |
| B Up | 26 | Nfix, Sall2, Etv1, Nptxr, Trip6, Notch1, Zfp128, Polr2l, Ascl2, C1d, Polr2c, Sox8, Ppard, Thra, Bcl3, Zik1, Cdk9, Shox2, Erf, Hoxb4, Taf13, E2f1, Zfp42, Hmg20b, Nr1i3, Scand1 | transcription initiation from RNA polymerase II promoter negative regulation of myoblast differentiation chordate embryonic development Nuclear Receptor transcription pathway cell cycle arrest skeletal system morphogenesis |
| A Down | 23 | Mid1, Btbd11, Tulp4, Zfp287, Taf4b, Sirt5, Tbx20, Mcm5, Myt1, Pou4f1, Trim24, A630089N07Rik, 4930522L14Rik, Hdac6, Cited4, Zfp113, Mcm7, Ezh1, Gata1, Sox7, Sh3d19, Zfp9, Ank1 | PID HDAC classI pathway regulation of signal transduction by p53 class mediator cell fate commitment |
| B Down | 55 | Ehf, Snapc4, Dmrt2, Zfp27, Rai14, Sox6, Ppargc1a, Sirt4, Hes1, Fhl2, Nr1d2, Creb3l1, Fbxw7, Mybl1, Nr2f6, Sox13, Prdm5, Tcf7, Zmynd11, Ercc2, Tfap4, Hic2, Egr2, Trip13, Tead1, Dbp, Camk4, Rarb, Pqbp1, Klf7, Gcdh, Hic1, Foxo1, Six6, Nr3c2, Bach2, Sp6, Brca1, Ddit3, Elk4, Zfp202, Foxm1, E2f5, Hdac11, Prdm16, Bmi1, Nr1d1, Klf5, Zfp2, Atm, Rfxank, Mcm2, Helz, Gata3, Gfi1 | fat cell differentiation cellular response to steroid hormone stimulus histone modification regulation of DNA binding regulation of lipid metabolic process DNA damage response, signal transduction by p53 class mediator glial cell differentiation PID HDAC classIII pathway muscle tissue development negative regulation of cell differentiation |

## Slide 9
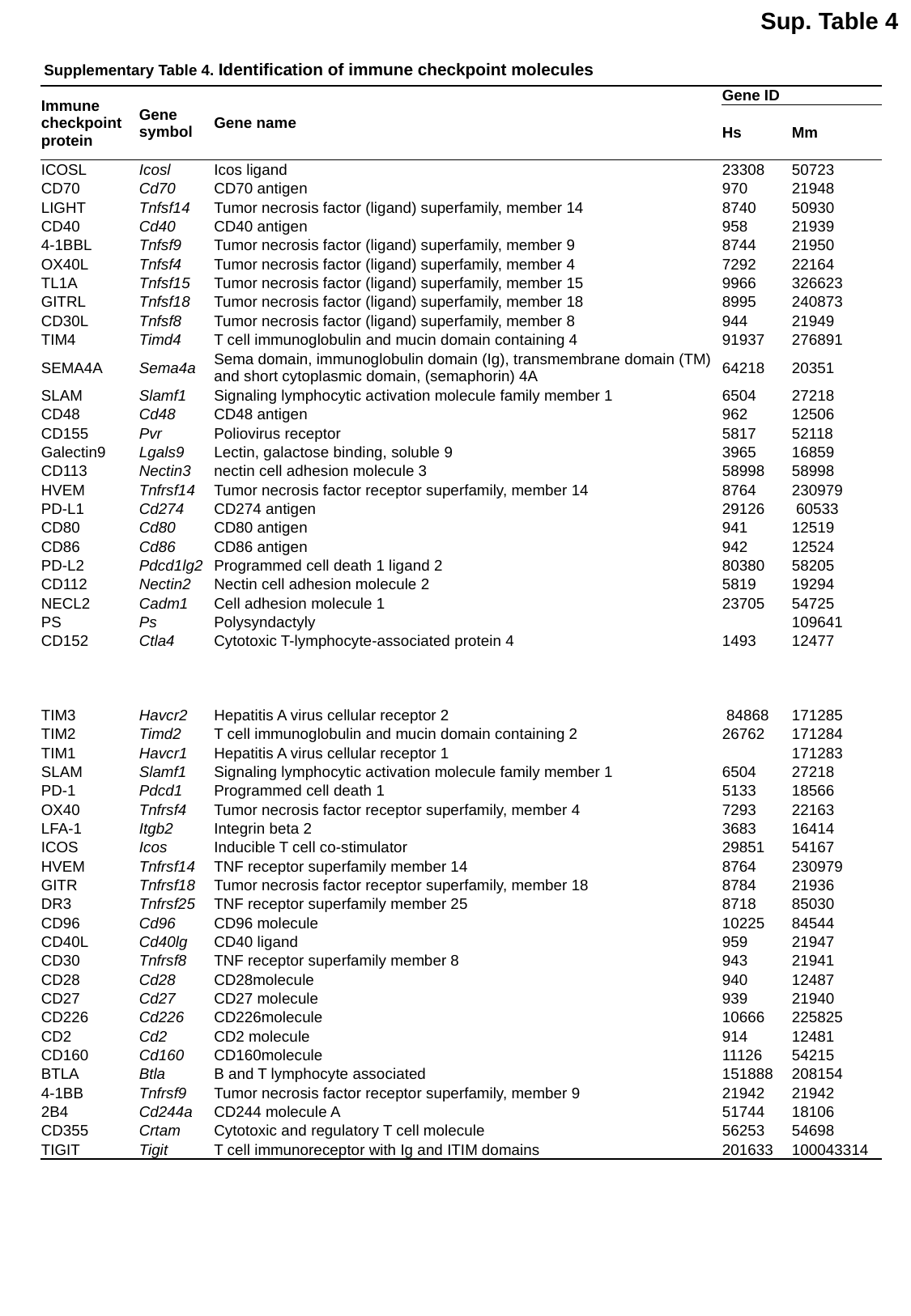

Sup. Table 4
Supplementary Table 4. Identification of immune checkpoint molecules
| Immune checkpoint protein | Gene symbol | Gene name | Gene ID | |
| --- | --- | --- | --- | --- |
| | | | Hs | Mm |
| ICOSL | Icosl | Icos ligand | 23308 | 50723 |
| CD70 | Cd70 | CD70 antigen | 970 | 21948 |
| LIGHT | Tnfsf14 | Tumor necrosis factor (ligand) superfamily, member 14 | 8740 | 50930 |
| CD40 | Cd40 | CD40 antigen | 958 | 21939 |
| 4-1BBL | Tnfsf9 | Tumor necrosis factor (ligand) superfamily, member 9 | 8744 | 21950 |
| OX40L | Tnfsf4 | Tumor necrosis factor (ligand) superfamily, member 4 | 7292 | 22164 |
| TL1A | Tnfsf15 | Tumor necrosis factor (ligand) superfamily, member 15 | 9966 | 326623 |
| GITRL | Tnfsf18 | Tumor necrosis factor (ligand) superfamily, member 18 | 8995 | 240873 |
| CD30L | Tnfsf8 | Tumor necrosis factor (ligand) superfamily, member 8 | 944 | 21949 |
| TIM4 | Timd4 | T cell immunoglobulin and mucin domain containing 4 | 91937 | 276891 |
| SEMA4A | Sema4a | Sema domain, immunoglobulin domain (Ig), transmembrane domain (TM) and short cytoplasmic domain, (semaphorin) 4A | 64218 | 20351 |
| SLAM | Slamf1 | Signaling lymphocytic activation molecule family member 1 | 6504 | 27218 |
| CD48 | Cd48 | CD48 antigen | 962 | 12506 |
| CD155 | Pvr | Poliovirus receptor | 5817 | 52118 |
| Galectin9 | Lgals9 | Lectin, galactose binding, soluble 9 | 3965 | 16859 |
| CD113 | Nectin3 | nectin cell adhesion molecule 3 | 58998 | 58998 |
| HVEM | Tnfrsf14 | Tumor necrosis factor receptor superfamily, member 14 | 8764 | 230979 |
| PD-L1 | Cd274 | CD274 antigen | 29126 | 60533 |
| CD80 | Cd80 | CD80 antigen | 941 | 12519 |
| CD86 | Cd86 | CD86 antigen | 942 | 12524 |
| PD-L2 | Pdcd1lg2 | Programmed cell death 1 ligand 2 | 80380 | 58205 |
| CD112 | Nectin2 | Nectin cell adhesion molecule 2 | 5819 | 19294 |
| NECL2 | Cadm1 | Cell adhesion molecule 1 | 23705 | 54725 |
| PS | Ps | Polysyndactyly | | 109641 |
| CD152 | Ctla4 | Cytotoxic T-lymphocyte-associated protein 4 | 1493 | 12477 |
| | | | | |
| TIM3 | Havcr2 | Hepatitis A virus cellular receptor 2 | 84868 | 171285 |
| TIM2 | Timd2 | T cell immunoglobulin and mucin domain containing 2 | 26762 | 171284 |
| TIM1 | Havcr1 | Hepatitis A virus cellular receptor 1 | | 171283 |
| SLAM | Slamf1 | Signaling lymphocytic activation molecule family member 1 | 6504 | 27218 |
| PD-1 | Pdcd1 | Programmed cell death 1 | 5133 | 18566 |
| OX40 | Tnfrsf4 | Tumor necrosis factor receptor superfamily, member 4 | 7293 | 22163 |
| LFA-1 | Itgb2 | Integrin beta 2 | 3683 | 16414 |
| ICOS | Icos | Inducible T cell co-stimulator | 29851 | 54167 |
| HVEM | Tnfrsf14 | TNF receptor superfamily member 14 | 8764 | 230979 |
| GITR | Tnfrsf18 | Tumor necrosis factor receptor superfamily, member 18 | 8784 | 21936 |
| DR3 | Tnfrsf25 | TNF receptor superfamily member 25 | 8718 | 85030 |
| CD96 | Cd96 | CD96 molecule | 10225 | 84544 |
| CD40L | Cd40lg | CD40 ligand | 959 | 21947 |
| CD30 | Tnfrsf8 | TNF receptor superfamily member 8 | 943 | 21941 |
| CD28 | Cd28 | CD28molecule | 940 | 12487 |
| CD27 | Cd27 | CD27 molecule | 939 | 21940 |
| CD226 | Cd226 | CD226molecule | 10666 | 225825 |
| CD2 | Cd2 | CD2 molecule | 914 | 12481 |
| CD160 | Cd160 | CD160molecule | 11126 | 54215 |
| BTLA | Btla | B and T lymphocyte associated | 151888 | 208154 |
| 4-1BB | Tnfrsf9 | Tumor necrosis factor receptor superfamily, member 9 | 21942 | 21942 |
| 2B4 | Cd244a | CD244 molecule A | 51744 | 18106 |
| CD355 | Crtam | Cytotoxic and regulatory T cell molecule | 56253 | 54698 |
| TIGIT | Tigit | T cell immunoreceptor with Ig and ITIM domains | 201633 | 100043314 |

## Slide 10
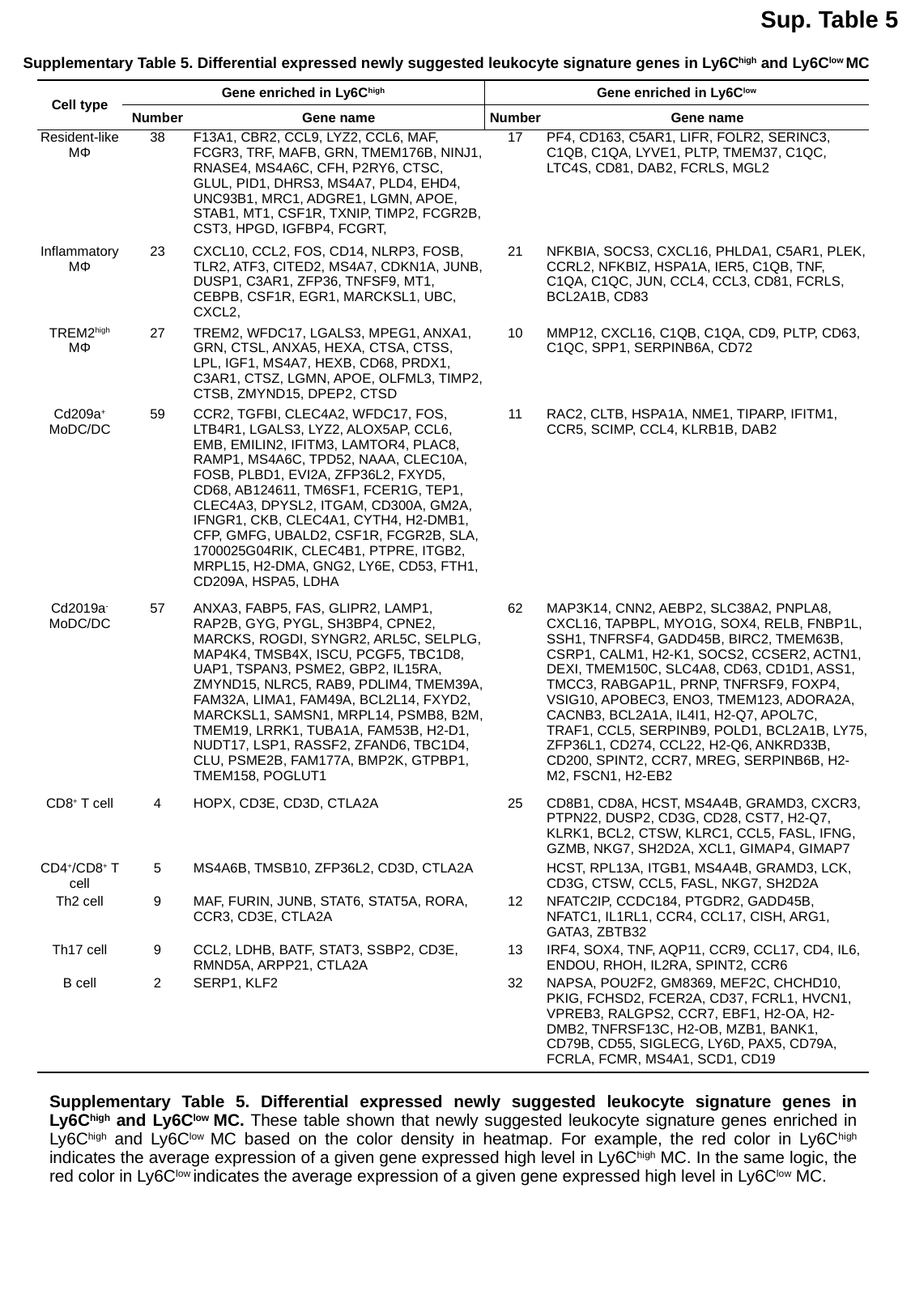

Sup. Table 5
Supplementary Table 5. Differential expressed newly suggested leukocyte signature genes in Ly6Chigh and Ly6Clow MC
| Cell type | Gene enriched in Ly6Chigh | | Gene enriched in Ly6Clow | |
| --- | --- | --- | --- | --- |
| | Number | Gene name | Number | Gene name |
| Resident-like MΦ | 38 | F13A1, CBR2, CCL9, LYZ2, CCL6, MAF, FCGR3, TRF, MAFB, GRN, TMEM176B, NINJ1, RNASE4, MS4A6C, CFH, P2RY6, CTSC, GLUL, PID1, DHRS3, MS4A7, PLD4, EHD4, UNC93B1, MRC1, ADGRE1, LGMN, APOE, STAB1, MT1, CSF1R, TXNIP, TIMP2, FCGR2B, CST3, HPGD, IGFBP4, FCGRT, | 17 | PF4, CD163, C5AR1, LIFR, FOLR2, SERINC3, C1QB, C1QA, LYVE1, PLTP, TMEM37, C1QC, LTC4S, CD81, DAB2, FCRLS, MGL2 |
| Inflammatory MΦ | 23 | CXCL10, CCL2, FOS, CD14, NLRP3, FOSB, TLR2, ATF3, CITED2, MS4A7, CDKN1A, JUNB, DUSP1, C3AR1, ZFP36, TNFSF9, MT1, CEBPB, CSF1R, EGR1, MARCKSL1, UBC, CXCL2, | 21 | NFKBIA, SOCS3, CXCL16, PHLDA1, C5AR1, PLEK, CCRL2, NFKBIZ, HSPA1A, IER5, C1QB, TNF, C1QA, C1QC, JUN, CCL4, CCL3, CD81, FCRLS, BCL2A1B, CD83 |
| TREM2high MΦ | 27 | TREM2, WFDC17, LGALS3, MPEG1, ANXA1, GRN, CTSL, ANXA5, HEXA, CTSA, CTSS, LPL, IGF1, MS4A7, HEXB, CD68, PRDX1, C3AR1, CTSZ, LGMN, APOE, OLFML3, TIMP2, CTSB, ZMYND15, DPEP2, CTSD | 10 | MMP12, CXCL16, C1QB, C1QA, CD9, PLTP, CD63, C1QC, SPP1, SERPINB6A, CD72 |
| Cd209a+ MoDC/DC | 59 | CCR2, TGFBI, CLEC4A2, WFDC17, FOS, LTB4R1, LGALS3, LYZ2, ALOX5AP, CCL6, EMB, EMILIN2, IFITM3, LAMTOR4, PLAC8, RAMP1, MS4A6C, TPD52, NAAA, CLEC10A, FOSB, PLBD1, EVI2A, ZFP36L2, FXYD5, CD68, AB124611, TM6SF1, FCER1G, TEP1, CLEC4A3, DPYSL2, ITGAM, CD300A, GM2A, IFNGR1, CKB, CLEC4A1, CYTH4, H2-DMB1, CFP, GMFG, UBALD2, CSF1R, FCGR2B, SLA, 1700025G04RIK, CLEC4B1, PTPRE, ITGB2, MRPL15, H2-DMA, GNG2, LY6E, CD53, FTH1, CD209A, HSPA5, LDHA | 11 | RAC2, CLTB, HSPA1A, NME1, TIPARP, IFITM1, CCR5, SCIMP, CCL4, KLRB1B, DAB2 |
| Cd2019a-MoDC/DC | 57 | ANXA3, FABP5, FAS, GLIPR2, LAMP1, RAP2B, GYG, PYGL, SH3BP4, CPNE2, MARCKS, ROGDI, SYNGR2, ARL5C, SELPLG, MAP4K4, TMSB4X, ISCU, PCGF5, TBC1D8, UAP1, TSPAN3, PSME2, GBP2, IL15RA, ZMYND15, NLRC5, RAB9, PDLIM4, TMEM39A, FAM32A, LIMA1, FAM49A, BCL2L14, FXYD2, MARCKSL1, SAMSN1, MRPL14, PSMB8, B2M, TMEM19, LRRK1, TUBA1A, FAM53B, H2-D1, NUDT17, LSP1, RASSF2, ZFAND6, TBC1D4, CLU, PSME2B, FAM177A, BMP2K, GTPBP1, TMEM158, POGLUT1 | 62 | MAP3K14, CNN2, AEBP2, SLC38A2, PNPLA8, CXCL16, TAPBPL, MYO1G, SOX4, RELB, FNBP1L, SSH1, TNFRSF4, GADD45B, BIRC2, TMEM63B, CSRP1, CALM1, H2-K1, SOCS2, CCSER2, ACTN1, DEXI, TMEM150C, SLC4A8, CD63, CD1D1, ASS1, TMCC3, RABGAP1L, PRNP, TNFRSF9, FOXP4, VSIG10, APOBEC3, ENO3, TMEM123, ADORA2A, CACNB3, BCL2A1A, IL4I1, H2-Q7, APOL7C, TRAF1, CCL5, SERPINB9, POLD1, BCL2A1B, LY75, ZFP36L1, CD274, CCL22, H2-Q6, ANKRD33B, CD200, SPINT2, CCR7, MREG, SERPINB6B, H2-M2, FSCN1, H2-EB2 |
| CD8+ T cell | 4 | HOPX, CD3E, CD3D, CTLA2A | 25 | CD8B1, CD8A, HCST, MS4A4B, GRAMD3, CXCR3, PTPN22, DUSP2, CD3G, CD28, CST7, H2-Q7, KLRK1, BCL2, CTSW, KLRC1, CCL5, FASL, IFNG, GZMB, NKG7, SH2D2A, XCL1, GIMAP4, GIMAP7 |
| CD4+/CD8+ T cell | 5 | MS4A6B, TMSB10, ZFP36L2, CD3D, CTLA2A | | HCST, RPL13A, ITGB1, MS4A4B, GRAMD3, LCK, CD3G, CTSW, CCL5, FASL, NKG7, SH2D2A |
| Th2 cell | 9 | MAF, FURIN, JUNB, STAT6, STAT5A, RORA, CCR3, CD3E, CTLA2A | 12 | NFATC2IP, CCDC184, PTGDR2, GADD45B, NFATC1, IL1RL1, CCR4, CCL17, CISH, ARG1, GATA3, ZBTB32 |
| Th17 cell | 9 | CCL2, LDHB, BATF, STAT3, SSBP2, CD3E, RMND5A, ARPP21, CTLA2A | 13 | IRF4, SOX4, TNF, AQP11, CCR9, CCL17, CD4, IL6, ENDOU, RHOH, IL2RA, SPINT2, CCR6 |
| B cell | 2 | SERP1, KLF2 | 32 | NAPSA, POU2F2, GM8369, MEF2C, CHCHD10, PKIG, FCHSD2, FCER2A, CD37, FCRL1, HVCN1, VPREB3, RALGPS2, CCR7, EBF1, H2-OA, H2-DMB2, TNFRSF13C, H2-OB, MZB1, BANK1, CD79B, CD55, SIGLECG, LY6D, PAX5, CD79A, FCRLA, FCMR, MS4A1, SCD1, CD19 |
Supplementary Table 5. Differential expressed newly suggested leukocyte signature genes in Ly6Chigh and Ly6Clow MC. These table shown that newly suggested leukocyte signature genes enriched in Ly6Chigh and Ly6Clow MC based on the color density in heatmap. For example, the red color in Ly6Chigh indicates the average expression of a given gene expressed high level in Ly6Chigh MC. In the same logic, the red color in Ly6Clow indicates the average expression of a given gene expressed high level in Ly6Clow MC.

## Slide 11
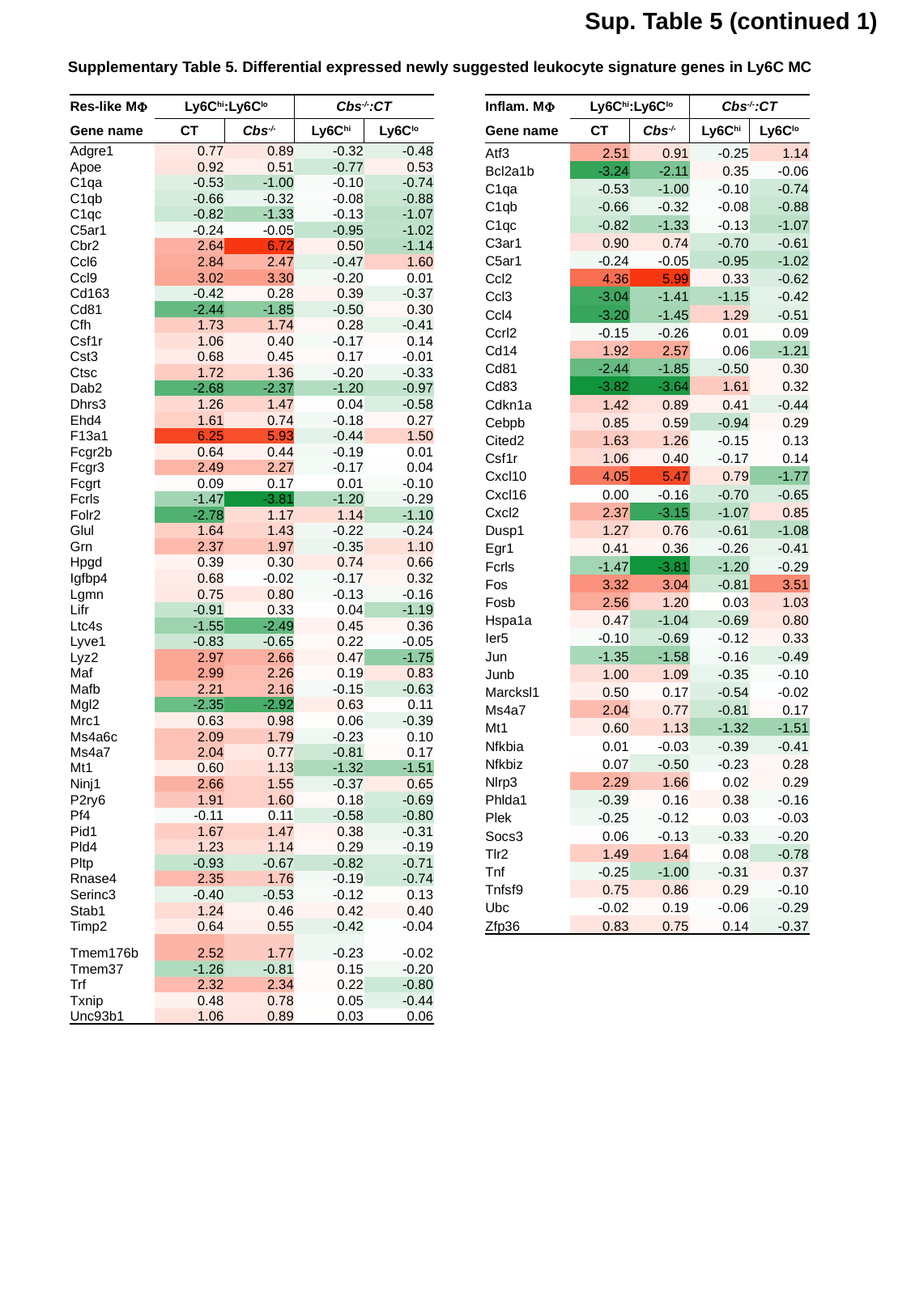

Sup. Table 5 (continued 1)
Supplementary Table 5. Differential expressed newly suggested leukocyte signature genes in Ly6C MC
| Res-like M | Ly6Chi:Ly6Clo | | Cbs-/-:CT | |
| --- | --- | --- | --- | --- |
| Gene name | CT | Cbs-/- | Ly6Chi | Ly6Clo |
| Adgre1 | 0.77 | 0.89 | -0.32 | -0.48 |
| Apoe | 0.92 | 0.51 | -0.77 | 0.53 |
| C1qa | -0.53 | -1.00 | -0.10 | -0.74 |
| C1qb | -0.66 | -0.32 | -0.08 | -0.88 |
| C1qc | -0.82 | -1.33 | -0.13 | -1.07 |
| C5ar1 | -0.24 | -0.05 | -0.95 | -1.02 |
| Cbr2 | 2.64 | 6.72 | 0.50 | -1.14 |
| Ccl6 | 2.84 | 2.47 | -0.47 | 1.60 |
| Ccl9 | 3.02 | 3.30 | -0.20 | 0.01 |
| Cd163 | -0.42 | 0.28 | 0.39 | -0.37 |
| Cd81 | -2.44 | -1.85 | -0.50 | 0.30 |
| Cfh | 1.73 | 1.74 | 0.28 | -0.41 |
| Csf1r | 1.06 | 0.40 | -0.17 | 0.14 |
| Cst3 | 0.68 | 0.45 | 0.17 | -0.01 |
| Ctsc | 1.72 | 1.36 | -0.20 | -0.33 |
| Dab2 | -2.68 | -2.37 | -1.20 | -0.97 |
| Dhrs3 | 1.26 | 1.47 | 0.04 | -0.58 |
| Ehd4 | 1.61 | 0.74 | -0.18 | 0.27 |
| F13a1 | 6.25 | 5.93 | -0.44 | 1.50 |
| Fcgr2b | 0.64 | 0.44 | -0.19 | 0.01 |
| Fcgr3 | 2.49 | 2.27 | -0.17 | 0.04 |
| Fcgrt | 0.09 | 0.17 | 0.01 | -0.10 |
| Fcrls | -1.47 | -3.81 | -1.20 | -0.29 |
| Folr2 | -2.78 | 1.17 | 1.14 | -1.10 |
| Glul | 1.64 | 1.43 | -0.22 | -0.24 |
| Grn | 2.37 | 1.97 | -0.35 | 1.10 |
| Hpgd | 0.39 | 0.30 | 0.74 | 0.66 |
| Igfbp4 | 0.68 | -0.02 | -0.17 | 0.32 |
| Lgmn | 0.75 | 0.80 | -0.13 | -0.16 |
| Lifr | -0.91 | 0.33 | 0.04 | -1.19 |
| Ltc4s | -1.55 | -2.49 | 0.45 | 0.36 |
| Lyve1 | -0.83 | -0.65 | 0.22 | -0.05 |
| Lyz2 | 2.97 | 2.66 | 0.47 | -1.75 |
| Maf | 2.99 | 2.26 | 0.19 | 0.83 |
| Mafb | 2.21 | 2.16 | -0.15 | -0.63 |
| Mgl2 | -2.35 | -2.92 | 0.63 | 0.11 |
| Mrc1 | 0.63 | 0.98 | 0.06 | -0.39 |
| Ms4a6c | 2.09 | 1.79 | -0.23 | 0.10 |
| Ms4a7 | 2.04 | 0.77 | -0.81 | 0.17 |
| Mt1 | 0.60 | 1.13 | -1.32 | -1.51 |
| Ninj1 | 2.66 | 1.55 | -0.37 | 0.65 |
| P2ry6 | 1.91 | 1.60 | 0.18 | -0.69 |
| Pf4 | -0.11 | 0.11 | -0.58 | -0.80 |
| Pid1 | 1.67 | 1.47 | 0.38 | -0.31 |
| Pld4 | 1.23 | 1.14 | 0.29 | -0.19 |
| Pltp | -0.93 | -0.67 | -0.82 | -0.71 |
| Rnase4 | 2.35 | 1.76 | -0.19 | -0.74 |
| Serinc3 | -0.40 | -0.53 | -0.12 | 0.13 |
| Stab1 | 1.24 | 0.46 | 0.42 | 0.40 |
| Timp2 | 0.64 | 0.55 | -0.42 | -0.04 |
| Tmem176b | 2.52 | 1.77 | -0.23 | -0.02 |
| Tmem37 | -1.26 | -0.81 | 0.15 | -0.20 |
| Trf | 2.32 | 2.34 | 0.22 | -0.80 |
| Txnip | 0.48 | 0.78 | 0.05 | -0.44 |
| Unc93b1 | 1.06 | 0.89 | 0.03 | 0.06 |
| Inflam. M | Ly6Chi:Ly6Clo | | Cbs-/-:CT | |
| --- | --- | --- | --- | --- |
| Gene name | CT | Cbs-/- | Ly6Chi | Ly6Clo |
| Atf3 | 2.51 | 0.91 | -0.25 | 1.14 |
| Bcl2a1b | -3.24 | -2.11 | 0.35 | -0.06 |
| C1qa | -0.53 | -1.00 | -0.10 | -0.74 |
| C1qb | -0.66 | -0.32 | -0.08 | -0.88 |
| C1qc | -0.82 | -1.33 | -0.13 | -1.07 |
| C3ar1 | 0.90 | 0.74 | -0.70 | -0.61 |
| C5ar1 | -0.24 | -0.05 | -0.95 | -1.02 |
| Ccl2 | 4.36 | 5.99 | 0.33 | -0.62 |
| Ccl3 | -3.04 | -1.41 | -1.15 | -0.42 |
| Ccl4 | -3.20 | -1.45 | 1.29 | -0.51 |
| Ccrl2 | -0.15 | -0.26 | 0.01 | 0.09 |
| Cd14 | 1.92 | 2.57 | 0.06 | -1.21 |
| Cd81 | -2.44 | -1.85 | -0.50 | 0.30 |
| Cd83 | -3.82 | -3.64 | 1.61 | 0.32 |
| Cdkn1a | 1.42 | 0.89 | 0.41 | -0.44 |
| Cebpb | 0.85 | 0.59 | -0.94 | 0.29 |
| Cited2 | 1.63 | 1.26 | -0.15 | 0.13 |
| Csf1r | 1.06 | 0.40 | -0.17 | 0.14 |
| Cxcl10 | 4.05 | 5.47 | 0.79 | -1.77 |
| Cxcl16 | 0.00 | -0.16 | -0.70 | -0.65 |
| Cxcl2 | 2.37 | -3.15 | -1.07 | 0.85 |
| Dusp1 | 1.27 | 0.76 | -0.61 | -1.08 |
| Egr1 | 0.41 | 0.36 | -0.26 | -0.41 |
| Fcrls | -1.47 | -3.81 | -1.20 | -0.29 |
| Fos | 3.32 | 3.04 | -0.81 | 3.51 |
| Fosb | 2.56 | 1.20 | 0.03 | 1.03 |
| Hspa1a | 0.47 | -1.04 | -0.69 | 0.80 |
| Ier5 | -0.10 | -0.69 | -0.12 | 0.33 |
| Jun | -1.35 | -1.58 | -0.16 | -0.49 |
| Junb | 1.00 | 1.09 | -0.35 | -0.10 |
| Marcksl1 | 0.50 | 0.17 | -0.54 | -0.02 |
| Ms4a7 | 2.04 | 0.77 | -0.81 | 0.17 |
| Mt1 | 0.60 | 1.13 | -1.32 | -1.51 |
| Nfkbia | 0.01 | -0.03 | -0.39 | -0.41 |
| Nfkbiz | 0.07 | -0.50 | -0.23 | 0.28 |
| Nlrp3 | 2.29 | 1.66 | 0.02 | 0.29 |
| Phlda1 | -0.39 | 0.16 | 0.38 | -0.16 |
| Plek | -0.25 | -0.12 | 0.03 | -0.03 |
| Socs3 | 0.06 | -0.13 | -0.33 | -0.20 |
| Tlr2 | 1.49 | 1.64 | 0.08 | -0.78 |
| Tnf | -0.25 | -1.00 | -0.31 | 0.37 |
| Tnfsf9 | 0.75 | 0.86 | 0.29 | -0.10 |
| Ubc | -0.02 | 0.19 | -0.06 | -0.29 |
| Zfp36 | 0.83 | 0.75 | 0.14 | -0.37 |

## Slide 12
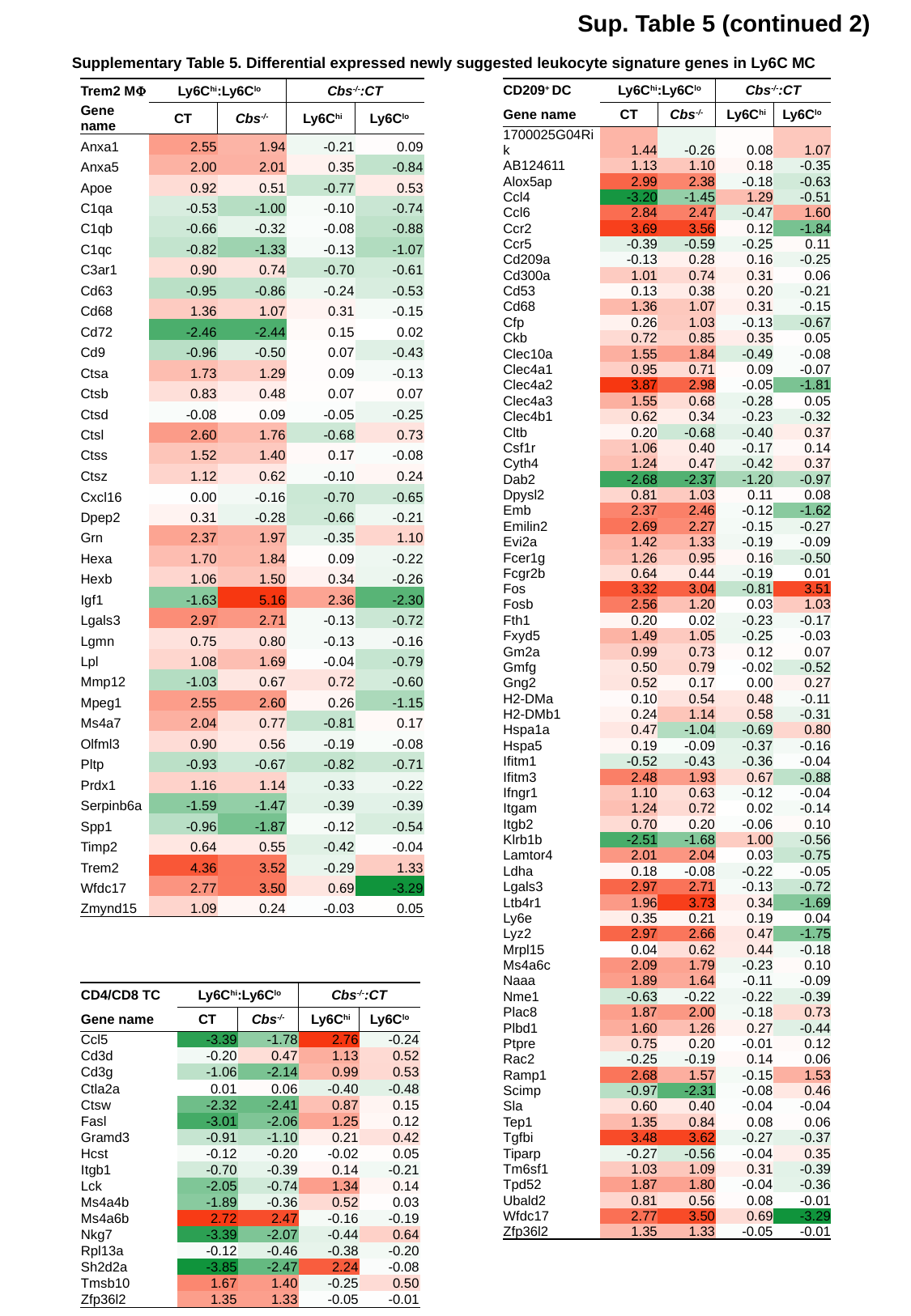

Sup. Table 5 (continued 2)
Supplementary Table 5. Differential expressed newly suggested leukocyte signature genes in Ly6C MC
| Trem2 M | Ly6Chi:Ly6Clo | | Cbs-/-:CT | |
| --- | --- | --- | --- | --- |
| Gene name | CT | Cbs-/- | Ly6Chi | Ly6Clo |
| Anxa1 | 2.55 | 1.94 | -0.21 | 0.09 |
| Anxa5 | 2.00 | 2.01 | 0.35 | -0.84 |
| Apoe | 0.92 | 0.51 | -0.77 | 0.53 |
| C1qa | -0.53 | -1.00 | -0.10 | -0.74 |
| C1qb | -0.66 | -0.32 | -0.08 | -0.88 |
| C1qc | -0.82 | -1.33 | -0.13 | -1.07 |
| C3ar1 | 0.90 | 0.74 | -0.70 | -0.61 |
| Cd63 | -0.95 | -0.86 | -0.24 | -0.53 |
| Cd68 | 1.36 | 1.07 | 0.31 | -0.15 |
| Cd72 | -2.46 | -2.44 | 0.15 | 0.02 |
| Cd9 | -0.96 | -0.50 | 0.07 | -0.43 |
| Ctsa | 1.73 | 1.29 | 0.09 | -0.13 |
| Ctsb | 0.83 | 0.48 | 0.07 | 0.07 |
| Ctsd | -0.08 | 0.09 | -0.05 | -0.25 |
| Ctsl | 2.60 | 1.76 | -0.68 | 0.73 |
| Ctss | 1.52 | 1.40 | 0.17 | -0.08 |
| Ctsz | 1.12 | 0.62 | -0.10 | 0.24 |
| Cxcl16 | 0.00 | -0.16 | -0.70 | -0.65 |
| Dpep2 | 0.31 | -0.28 | -0.66 | -0.21 |
| Grn | 2.37 | 1.97 | -0.35 | 1.10 |
| Hexa | 1.70 | 1.84 | 0.09 | -0.22 |
| Hexb | 1.06 | 1.50 | 0.34 | -0.26 |
| Igf1 | -1.63 | 5.16 | 2.36 | -2.30 |
| Lgals3 | 2.97 | 2.71 | -0.13 | -0.72 |
| Lgmn | 0.75 | 0.80 | -0.13 | -0.16 |
| Lpl | 1.08 | 1.69 | -0.04 | -0.79 |
| Mmp12 | -1.03 | 0.67 | 0.72 | -0.60 |
| Mpeg1 | 2.55 | 2.60 | 0.26 | -1.15 |
| Ms4a7 | 2.04 | 0.77 | -0.81 | 0.17 |
| Olfml3 | 0.90 | 0.56 | -0.19 | -0.08 |
| Pltp | -0.93 | -0.67 | -0.82 | -0.71 |
| Prdx1 | 1.16 | 1.14 | -0.33 | -0.22 |
| Serpinb6a | -1.59 | -1.47 | -0.39 | -0.39 |
| Spp1 | -0.96 | -1.87 | -0.12 | -0.54 |
| Timp2 | 0.64 | 0.55 | -0.42 | -0.04 |
| Trem2 | 4.36 | 3.52 | -0.29 | 1.33 |
| Wfdc17 | 2.77 | 3.50 | 0.69 | -3.29 |
| Zmynd15 | 1.09 | 0.24 | -0.03 | 0.05 |
| CD209+ DC | Ly6Chi:Ly6Clo | | Cbs-/-:CT | |
| --- | --- | --- | --- | --- |
| Gene name | CT | Cbs-/- | Ly6Chi | Ly6Clo |
| 1700025G04Rik | 1.44 | -0.26 | 0.08 | 1.07 |
| AB124611 | 1.13 | 1.10 | 0.18 | -0.35 |
| Alox5ap | 2.99 | 2.38 | -0.18 | -0.63 |
| Ccl4 | -3.20 | -1.45 | 1.29 | -0.51 |
| Ccl6 | 2.84 | 2.47 | -0.47 | 1.60 |
| Ccr2 | 3.69 | 3.56 | 0.12 | -1.84 |
| Ccr5 | -0.39 | -0.59 | -0.25 | 0.11 |
| Cd209a | -0.13 | 0.28 | 0.16 | -0.25 |
| Cd300a | 1.01 | 0.74 | 0.31 | 0.06 |
| Cd53 | 0.13 | 0.38 | 0.20 | -0.21 |
| Cd68 | 1.36 | 1.07 | 0.31 | -0.15 |
| Cfp | 0.26 | 1.03 | -0.13 | -0.67 |
| Ckb | 0.72 | 0.85 | 0.35 | 0.05 |
| Clec10a | 1.55 | 1.84 | -0.49 | -0.08 |
| Clec4a1 | 0.95 | 0.71 | 0.09 | -0.07 |
| Clec4a2 | 3.87 | 2.98 | -0.05 | -1.81 |
| Clec4a3 | 1.55 | 0.68 | -0.28 | 0.05 |
| Clec4b1 | 0.62 | 0.34 | -0.23 | -0.32 |
| Cltb | 0.20 | -0.68 | -0.40 | 0.37 |
| Csf1r | 1.06 | 0.40 | -0.17 | 0.14 |
| Cyth4 | 1.24 | 0.47 | -0.42 | 0.37 |
| Dab2 | -2.68 | -2.37 | -1.20 | -0.97 |
| Dpysl2 | 0.81 | 1.03 | 0.11 | 0.08 |
| Emb | 2.37 | 2.46 | -0.12 | -1.62 |
| Emilin2 | 2.69 | 2.27 | -0.15 | -0.27 |
| Evi2a | 1.42 | 1.33 | -0.19 | -0.09 |
| Fcer1g | 1.26 | 0.95 | 0.16 | -0.50 |
| Fcgr2b | 0.64 | 0.44 | -0.19 | 0.01 |
| Fos | 3.32 | 3.04 | -0.81 | 3.51 |
| Fosb | 2.56 | 1.20 | 0.03 | 1.03 |
| Fth1 | 0.20 | 0.02 | -0.23 | -0.17 |
| Fxyd5 | 1.49 | 1.05 | -0.25 | -0.03 |
| Gm2a | 0.99 | 0.73 | 0.12 | 0.07 |
| Gmfg | 0.50 | 0.79 | -0.02 | -0.52 |
| Gng2 | 0.52 | 0.17 | 0.00 | 0.27 |
| H2-DMa | 0.10 | 0.54 | 0.48 | -0.11 |
| H2-DMb1 | 0.24 | 1.14 | 0.58 | -0.31 |
| Hspa1a | 0.47 | -1.04 | -0.69 | 0.80 |
| Hspa5 | 0.19 | -0.09 | -0.37 | -0.16 |
| Ifitm1 | -0.52 | -0.43 | -0.36 | -0.04 |
| Ifitm3 | 2.48 | 1.93 | 0.67 | -0.88 |
| Ifngr1 | 1.10 | 0.63 | -0.12 | -0.04 |
| Itgam | 1.24 | 0.72 | 0.02 | -0.14 |
| Itgb2 | 0.70 | 0.20 | -0.06 | 0.10 |
| Klrb1b | -2.51 | -1.68 | 1.00 | -0.56 |
| Lamtor4 | 2.01 | 2.04 | 0.03 | -0.75 |
| Ldha | 0.18 | -0.08 | -0.22 | -0.05 |
| Lgals3 | 2.97 | 2.71 | -0.13 | -0.72 |
| Ltb4r1 | 1.96 | 3.73 | 0.34 | -1.69 |
| Ly6e | 0.35 | 0.21 | 0.19 | 0.04 |
| Lyz2 | 2.97 | 2.66 | 0.47 | -1.75 |
| Mrpl15 | 0.04 | 0.62 | 0.44 | -0.18 |
| Ms4a6c | 2.09 | 1.79 | -0.23 | 0.10 |
| Naaa | 1.89 | 1.64 | -0.11 | -0.09 |
| Nme1 | -0.63 | -0.22 | -0.22 | -0.39 |
| Plac8 | 1.87 | 2.00 | -0.18 | 0.73 |
| Plbd1 | 1.60 | 1.26 | 0.27 | -0.44 |
| Ptpre | 0.75 | 0.20 | -0.01 | 0.12 |
| Rac2 | -0.25 | -0.19 | 0.14 | 0.06 |
| Ramp1 | 2.68 | 1.57 | -0.15 | 1.53 |
| Scimp | -0.97 | -2.31 | -0.08 | 0.46 |
| Sla | 0.60 | 0.40 | -0.04 | -0.04 |
| Tep1 | 1.35 | 0.84 | 0.08 | 0.06 |
| Tgfbi | 3.48 | 3.62 | -0.27 | -0.37 |
| Tiparp | -0.27 | -0.56 | -0.04 | 0.35 |
| Tm6sf1 | 1.03 | 1.09 | 0.31 | -0.39 |
| Tpd52 | 1.87 | 1.80 | -0.04 | -0.36 |
| Ubald2 | 0.81 | 0.56 | 0.08 | -0.01 |
| Wfdc17 | 2.77 | 3.50 | 0.69 | -3.29 |
| Zfp36l2 | 1.35 | 1.33 | -0.05 | -0.01 |
| CD4/CD8 TC | Ly6Chi:Ly6Clo | | Cbs-/-:CT | |
| --- | --- | --- | --- | --- |
| Gene name | CT | Cbs-/- | Ly6Chi | Ly6Clo |
| Ccl5 | -3.39 | -1.78 | 2.76 | -0.24 |
| Cd3d | -0.20 | 0.47 | 1.13 | 0.52 |
| Cd3g | -1.06 | -2.14 | 0.99 | 0.53 |
| Ctla2a | 0.01 | 0.06 | -0.40 | -0.48 |
| Ctsw | -2.32 | -2.41 | 0.87 | 0.15 |
| Fasl | -3.01 | -2.06 | 1.25 | 0.12 |
| Gramd3 | -0.91 | -1.10 | 0.21 | 0.42 |
| Hcst | -0.12 | -0.20 | -0.02 | 0.05 |
| Itgb1 | -0.70 | -0.39 | 0.14 | -0.21 |
| Lck | -2.05 | -0.74 | 1.34 | 0.14 |
| Ms4a4b | -1.89 | -0.36 | 0.52 | 0.03 |
| Ms4a6b | 2.72 | 2.47 | -0.16 | -0.19 |
| Nkg7 | -3.39 | -2.07 | -0.44 | 0.64 |
| Rpl13a | -0.12 | -0.46 | -0.38 | -0.20 |
| Sh2d2a | -3.85 | -2.47 | 2.24 | -0.08 |
| Tmsb10 | 1.67 | 1.40 | -0.25 | 0.50 |
| Zfp36l2 | 1.35 | 1.33 | -0.05 | -0.01 |

## Slide 13
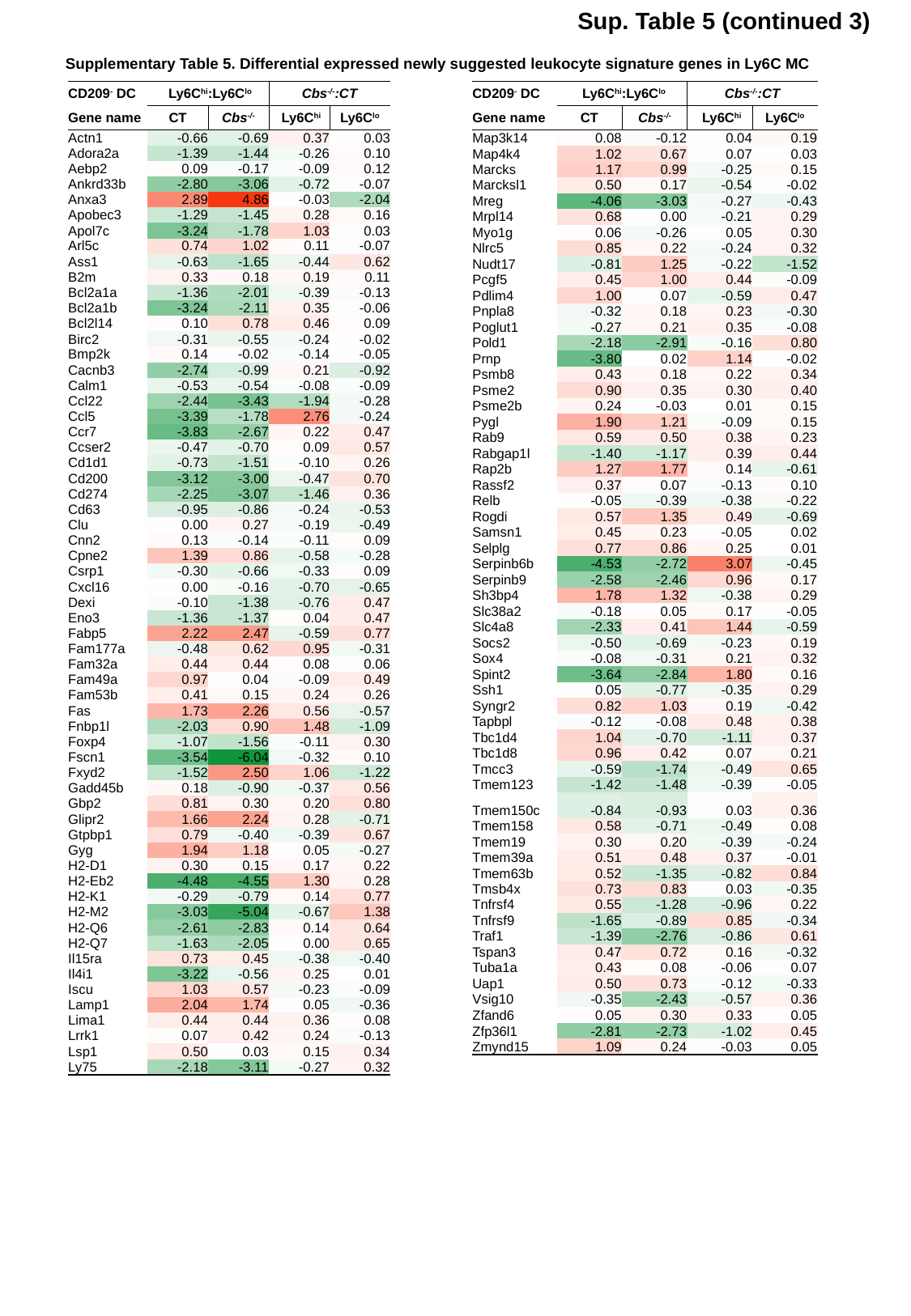

Sup. Table 5 (continued 3)
Supplementary Table 5. Differential expressed newly suggested leukocyte signature genes in Ly6C MC
| CD209- DC | Ly6Chi:Ly6Clo | | Cbs-/-:CT | |
| --- | --- | --- | --- | --- |
| Gene name | CT | Cbs-/- | Ly6Chi | Ly6Clo |
| Actn1 | -0.66 | -0.69 | 0.37 | 0.03 |
| Adora2a | -1.39 | -1.44 | -0.26 | 0.10 |
| Aebp2 | 0.09 | -0.17 | -0.09 | 0.12 |
| Ankrd33b | -2.80 | -3.06 | -0.72 | -0.07 |
| Anxa3 | 2.89 | 4.86 | -0.03 | -2.04 |
| Apobec3 | -1.29 | -1.45 | 0.28 | 0.16 |
| Apol7c | -3.24 | -1.78 | 1.03 | 0.03 |
| Arl5c | 0.74 | 1.02 | 0.11 | -0.07 |
| Ass1 | -0.63 | -1.65 | -0.44 | 0.62 |
| B2m | 0.33 | 0.18 | 0.19 | 0.11 |
| Bcl2a1a | -1.36 | -2.01 | -0.39 | -0.13 |
| Bcl2a1b | -3.24 | -2.11 | 0.35 | -0.06 |
| Bcl2l14 | 0.10 | 0.78 | 0.46 | 0.09 |
| Birc2 | -0.31 | -0.55 | -0.24 | -0.02 |
| Bmp2k | 0.14 | -0.02 | -0.14 | -0.05 |
| Cacnb3 | -2.74 | -0.99 | 0.21 | -0.92 |
| Calm1 | -0.53 | -0.54 | -0.08 | -0.09 |
| Ccl22 | -2.44 | -3.43 | -1.94 | -0.28 |
| Ccl5 | -3.39 | -1.78 | 2.76 | -0.24 |
| Ccr7 | -3.83 | -2.67 | 0.22 | 0.47 |
| Ccser2 | -0.47 | -0.70 | 0.09 | 0.57 |
| Cd1d1 | -0.73 | -1.51 | -0.10 | 0.26 |
| Cd200 | -3.12 | -3.00 | -0.47 | 0.70 |
| Cd274 | -2.25 | -3.07 | -1.46 | 0.36 |
| Cd63 | -0.95 | -0.86 | -0.24 | -0.53 |
| Clu | 0.00 | 0.27 | -0.19 | -0.49 |
| Cnn2 | 0.13 | -0.14 | -0.11 | 0.09 |
| Cpne2 | 1.39 | 0.86 | -0.58 | -0.28 |
| Csrp1 | -0.30 | -0.66 | -0.33 | 0.09 |
| Cxcl16 | 0.00 | -0.16 | -0.70 | -0.65 |
| Dexi | -0.10 | -1.38 | -0.76 | 0.47 |
| Eno3 | -1.36 | -1.37 | 0.04 | 0.47 |
| Fabp5 | 2.22 | 2.47 | -0.59 | 0.77 |
| Fam177a | -0.48 | 0.62 | 0.95 | -0.31 |
| Fam32a | 0.44 | 0.44 | 0.08 | 0.06 |
| Fam49a | 0.97 | 0.04 | -0.09 | 0.49 |
| Fam53b | 0.41 | 0.15 | 0.24 | 0.26 |
| Fas | 1.73 | 2.26 | 0.56 | -0.57 |
| Fnbp1l | -2.03 | 0.90 | 1.48 | -1.09 |
| Foxp4 | -1.07 | -1.56 | -0.11 | 0.30 |
| Fscn1 | -3.54 | -6.04 | -0.32 | 0.10 |
| Fxyd2 | -1.52 | 2.50 | 1.06 | -1.22 |
| Gadd45b | 0.18 | -0.90 | -0.37 | 0.56 |
| Gbp2 | 0.81 | 0.30 | 0.20 | 0.80 |
| Glipr2 | 1.66 | 2.24 | 0.28 | -0.71 |
| Gtpbp1 | 0.79 | -0.40 | -0.39 | 0.67 |
| Gyg | 1.94 | 1.18 | 0.05 | -0.27 |
| H2-D1 | 0.30 | 0.15 | 0.17 | 0.22 |
| H2-Eb2 | -4.48 | -4.55 | 1.30 | 0.28 |
| H2-K1 | -0.29 | -0.79 | 0.14 | 0.77 |
| H2-M2 | -3.03 | -5.04 | -0.67 | 1.38 |
| H2-Q6 | -2.61 | -2.83 | 0.14 | 0.64 |
| H2-Q7 | -1.63 | -2.05 | 0.00 | 0.65 |
| Il15ra | 0.73 | 0.45 | -0.38 | -0.40 |
| Il4i1 | -3.22 | -0.56 | 0.25 | 0.01 |
| Iscu | 1.03 | 0.57 | -0.23 | -0.09 |
| Lamp1 | 2.04 | 1.74 | 0.05 | -0.36 |
| Lima1 | 0.44 | 0.44 | 0.36 | 0.08 |
| Lrrk1 | 0.07 | 0.42 | 0.24 | -0.13 |
| Lsp1 | 0.50 | 0.03 | 0.15 | 0.34 |
| Ly75 | -2.18 | -3.11 | -0.27 | 0.32 |
| CD209- DC | Ly6Chi:Ly6Clo | | Cbs-/-:CT | |
| --- | --- | --- | --- | --- |
| Gene name | CT | Cbs-/- | Ly6Chi | Ly6Clo |
| Map3k14 | 0.08 | -0.12 | 0.04 | 0.19 |
| Map4k4 | 1.02 | 0.67 | 0.07 | 0.03 |
| Marcks | 1.17 | 0.99 | -0.25 | 0.15 |
| Marcksl1 | 0.50 | 0.17 | -0.54 | -0.02 |
| Mreg | -4.06 | -3.03 | -0.27 | -0.43 |
| Mrpl14 | 0.68 | 0.00 | -0.21 | 0.29 |
| Myo1g | 0.06 | -0.26 | 0.05 | 0.30 |
| Nlrc5 | 0.85 | 0.22 | -0.24 | 0.32 |
| Nudt17 | -0.81 | 1.25 | -0.22 | -1.52 |
| Pcgf5 | 0.45 | 1.00 | 0.44 | -0.09 |
| Pdlim4 | 1.00 | 0.07 | -0.59 | 0.47 |
| Pnpla8 | -0.32 | 0.18 | 0.23 | -0.30 |
| Poglut1 | -0.27 | 0.21 | 0.35 | -0.08 |
| Pold1 | -2.18 | -2.91 | -0.16 | 0.80 |
| Prnp | -3.80 | 0.02 | 1.14 | -0.02 |
| Psmb8 | 0.43 | 0.18 | 0.22 | 0.34 |
| Psme2 | 0.90 | 0.35 | 0.30 | 0.40 |
| Psme2b | 0.24 | -0.03 | 0.01 | 0.15 |
| Pygl | 1.90 | 1.21 | -0.09 | 0.15 |
| Rab9 | 0.59 | 0.50 | 0.38 | 0.23 |
| Rabgap1l | -1.40 | -1.17 | 0.39 | 0.44 |
| Rap2b | 1.27 | 1.77 | 0.14 | -0.61 |
| Rassf2 | 0.37 | 0.07 | -0.13 | 0.10 |
| Relb | -0.05 | -0.39 | -0.38 | -0.22 |
| Rogdi | 0.57 | 1.35 | 0.49 | -0.69 |
| Samsn1 | 0.45 | 0.23 | -0.05 | 0.02 |
| Selplg | 0.77 | 0.86 | 0.25 | 0.01 |
| Serpinb6b | -4.53 | -2.72 | 3.07 | -0.45 |
| Serpinb9 | -2.58 | -2.46 | 0.96 | 0.17 |
| Sh3bp4 | 1.78 | 1.32 | -0.38 | 0.29 |
| Slc38a2 | -0.18 | 0.05 | 0.17 | -0.05 |
| Slc4a8 | -2.33 | 0.41 | 1.44 | -0.59 |
| Socs2 | -0.50 | -0.69 | -0.23 | 0.19 |
| Sox4 | -0.08 | -0.31 | 0.21 | 0.32 |
| Spint2 | -3.64 | -2.84 | 1.80 | 0.16 |
| Ssh1 | 0.05 | -0.77 | -0.35 | 0.29 |
| Syngr2 | 0.82 | 1.03 | 0.19 | -0.42 |
| Tapbpl | -0.12 | -0.08 | 0.48 | 0.38 |
| Tbc1d4 | 1.04 | -0.70 | -1.11 | 0.37 |
| Tbc1d8 | 0.96 | 0.42 | 0.07 | 0.21 |
| Tmcc3 | -0.59 | -1.74 | -0.49 | 0.65 |
| Tmem123 | -1.42 | -1.48 | -0.39 | -0.05 |
| Tmem150c | -0.84 | -0.93 | 0.03 | 0.36 |
| Tmem158 | 0.58 | -0.71 | -0.49 | 0.08 |
| Tmem19 | 0.30 | 0.20 | -0.39 | -0.24 |
| Tmem39a | 0.51 | 0.48 | 0.37 | -0.01 |
| Tmem63b | 0.52 | -1.35 | -0.82 | 0.84 |
| Tmsb4x | 0.73 | 0.83 | 0.03 | -0.35 |
| Tnfrsf4 | 0.55 | -1.28 | -0.96 | 0.22 |
| Tnfrsf9 | -1.65 | -0.89 | 0.85 | -0.34 |
| Traf1 | -1.39 | -2.76 | -0.86 | 0.61 |
| Tspan3 | 0.47 | 0.72 | 0.16 | -0.32 |
| Tuba1a | 0.43 | 0.08 | -0.06 | 0.07 |
| Uap1 | 0.50 | 0.73 | -0.12 | -0.33 |
| Vsig10 | -0.35 | -2.43 | -0.57 | 0.36 |
| Zfand6 | 0.05 | 0.30 | 0.33 | 0.05 |
| Zfp36l1 | -2.81 | -2.73 | -1.02 | 0.45 |
| Zmynd15 | 1.09 | 0.24 | -0.03 | 0.05 |

## Slide 14
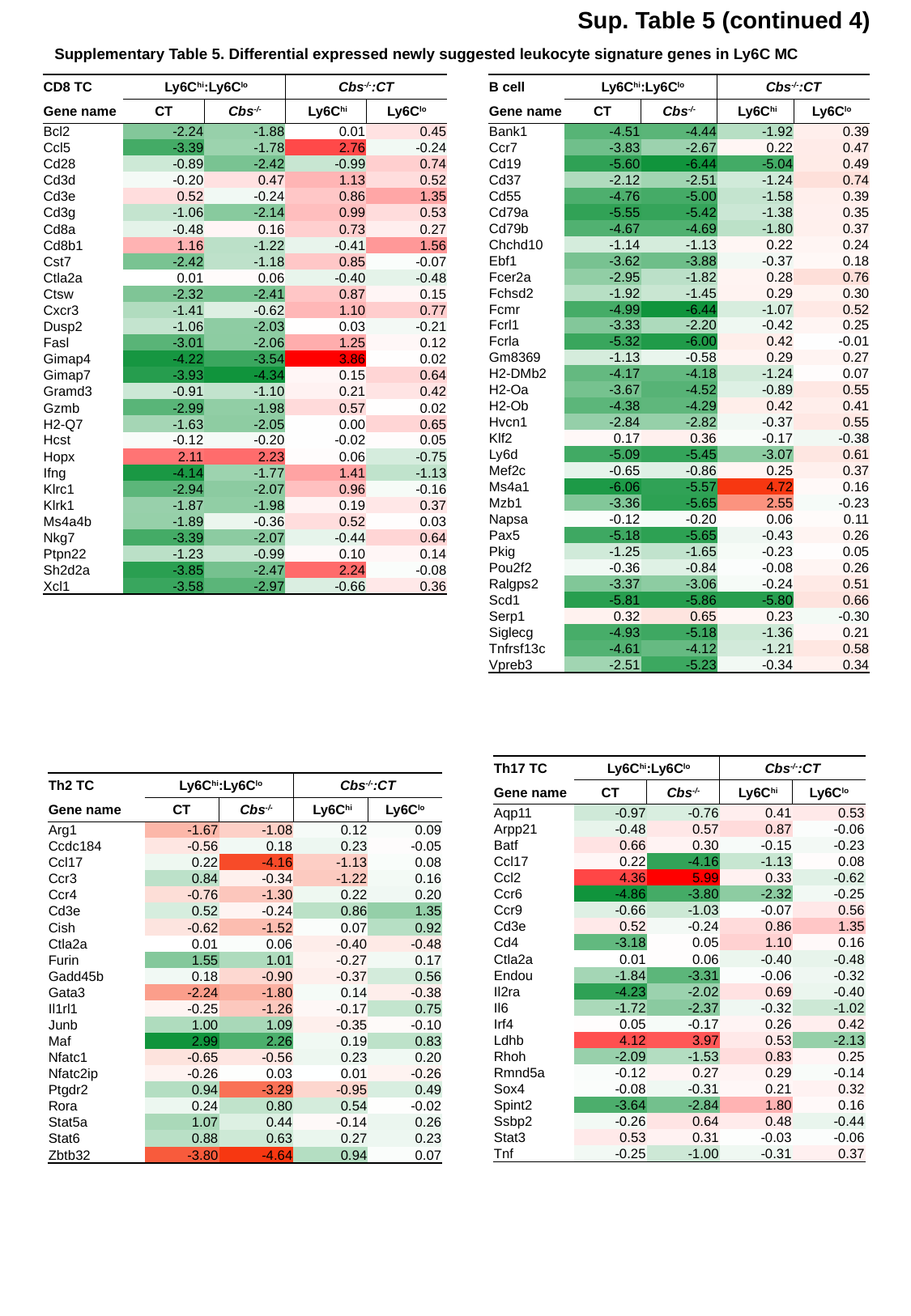

Sup. Table 5 (continued 4)
Supplementary Table 5. Differential expressed newly suggested leukocyte signature genes in Ly6C MC
| CD8 TC | Ly6Chi:Ly6Clo | | Cbs-/-:CT | |
| --- | --- | --- | --- | --- |
| Gene name | CT | Cbs-/- | Ly6Chi | Ly6Clo |
| Bcl2 | -2.24 | -1.88 | 0.01 | 0.45 |
| Ccl5 | -3.39 | -1.78 | 2.76 | -0.24 |
| Cd28 | -0.89 | -2.42 | -0.99 | 0.74 |
| Cd3d | -0.20 | 0.47 | 1.13 | 0.52 |
| Cd3e | 0.52 | -0.24 | 0.86 | 1.35 |
| Cd3g | -1.06 | -2.14 | 0.99 | 0.53 |
| Cd8a | -0.48 | 0.16 | 0.73 | 0.27 |
| Cd8b1 | 1.16 | -1.22 | -0.41 | 1.56 |
| Cst7 | -2.42 | -1.18 | 0.85 | -0.07 |
| Ctla2a | 0.01 | 0.06 | -0.40 | -0.48 |
| Ctsw | -2.32 | -2.41 | 0.87 | 0.15 |
| Cxcr3 | -1.41 | -0.62 | 1.10 | 0.77 |
| Dusp2 | -1.06 | -2.03 | 0.03 | -0.21 |
| Fasl | -3.01 | -2.06 | 1.25 | 0.12 |
| Gimap4 | -4.22 | -3.54 | 3.86 | 0.02 |
| Gimap7 | -3.93 | -4.34 | 0.15 | 0.64 |
| Gramd3 | -0.91 | -1.10 | 0.21 | 0.42 |
| Gzmb | -2.99 | -1.98 | 0.57 | 0.02 |
| H2-Q7 | -1.63 | -2.05 | 0.00 | 0.65 |
| Hcst | -0.12 | -0.20 | -0.02 | 0.05 |
| Hopx | 2.11 | 2.23 | 0.06 | -0.75 |
| Ifng | -4.14 | -1.77 | 1.41 | -1.13 |
| Klrc1 | -2.94 | -2.07 | 0.96 | -0.16 |
| Klrk1 | -1.87 | -1.98 | 0.19 | 0.37 |
| Ms4a4b | -1.89 | -0.36 | 0.52 | 0.03 |
| Nkg7 | -3.39 | -2.07 | -0.44 | 0.64 |
| Ptpn22 | -1.23 | -0.99 | 0.10 | 0.14 |
| Sh2d2a | -3.85 | -2.47 | 2.24 | -0.08 |
| Xcl1 | -3.58 | -2.97 | -0.66 | 0.36 |
| B cell | Ly6Chi:Ly6Clo | | Cbs-/-:CT | |
| --- | --- | --- | --- | --- |
| Gene name | CT | Cbs-/- | Ly6Chi | Ly6Clo |
| Bank1 | -4.51 | -4.44 | -1.92 | 0.39 |
| Ccr7 | -3.83 | -2.67 | 0.22 | 0.47 |
| Cd19 | -5.60 | -6.44 | -5.04 | 0.49 |
| Cd37 | -2.12 | -2.51 | -1.24 | 0.74 |
| Cd55 | -4.76 | -5.00 | -1.58 | 0.39 |
| Cd79a | -5.55 | -5.42 | -1.38 | 0.35 |
| Cd79b | -4.67 | -4.69 | -1.80 | 0.37 |
| Chchd10 | -1.14 | -1.13 | 0.22 | 0.24 |
| Ebf1 | -3.62 | -3.88 | -0.37 | 0.18 |
| Fcer2a | -2.95 | -1.82 | 0.28 | 0.76 |
| Fchsd2 | -1.92 | -1.45 | 0.29 | 0.30 |
| Fcmr | -4.99 | -6.44 | -1.07 | 0.52 |
| Fcrl1 | -3.33 | -2.20 | -0.42 | 0.25 |
| Fcrla | -5.32 | -6.00 | 0.42 | -0.01 |
| Gm8369 | -1.13 | -0.58 | 0.29 | 0.27 |
| H2-DMb2 | -4.17 | -4.18 | -1.24 | 0.07 |
| H2-Oa | -3.67 | -4.52 | -0.89 | 0.55 |
| H2-Ob | -4.38 | -4.29 | 0.42 | 0.41 |
| Hvcn1 | -2.84 | -2.82 | -0.37 | 0.55 |
| Klf2 | 0.17 | 0.36 | -0.17 | -0.38 |
| Ly6d | -5.09 | -5.45 | -3.07 | 0.61 |
| Mef2c | -0.65 | -0.86 | 0.25 | 0.37 |
| Ms4a1 | -6.06 | -5.57 | 4.72 | 0.16 |
| Mzb1 | -3.36 | -5.65 | 2.55 | -0.23 |
| Napsa | -0.12 | -0.20 | 0.06 | 0.11 |
| Pax5 | -5.18 | -5.65 | -0.43 | 0.26 |
| Pkig | -1.25 | -1.65 | -0.23 | 0.05 |
| Pou2f2 | -0.36 | -0.84 | -0.08 | 0.26 |
| Ralgps2 | -3.37 | -3.06 | -0.24 | 0.51 |
| Scd1 | -5.81 | -5.86 | -5.80 | 0.66 |
| Serp1 | 0.32 | 0.65 | 0.23 | -0.30 |
| Siglecg | -4.93 | -5.18 | -1.36 | 0.21 |
| Tnfrsf13c | -4.61 | -4.12 | -1.21 | 0.58 |
| Vpreb3 | -2.51 | -5.23 | -0.34 | 0.34 |
| Th17 TC | Ly6Chi:Ly6Clo | | Cbs-/-:CT | |
| --- | --- | --- | --- | --- |
| Gene name | CT | Cbs-/- | Ly6Chi | Ly6Clo |
| Aqp11 | -0.97 | -0.76 | 0.41 | 0.53 |
| Arpp21 | -0.48 | 0.57 | 0.87 | -0.06 |
| Batf | 0.66 | 0.30 | -0.15 | -0.23 |
| Ccl17 | 0.22 | -4.16 | -1.13 | 0.08 |
| Ccl2 | 4.36 | 5.99 | 0.33 | -0.62 |
| Ccr6 | -4.86 | -3.80 | -2.32 | -0.25 |
| Ccr9 | -0.66 | -1.03 | -0.07 | 0.56 |
| Cd3e | 0.52 | -0.24 | 0.86 | 1.35 |
| Cd4 | -3.18 | 0.05 | 1.10 | 0.16 |
| Ctla2a | 0.01 | 0.06 | -0.40 | -0.48 |
| Endou | -1.84 | -3.31 | -0.06 | -0.32 |
| Il2ra | -4.23 | -2.02 | 0.69 | -0.40 |
| Il6 | -1.72 | -2.37 | -0.32 | -1.02 |
| Irf4 | 0.05 | -0.17 | 0.26 | 0.42 |
| Ldhb | 4.12 | 3.97 | 0.53 | -2.13 |
| Rhoh | -2.09 | -1.53 | 0.83 | 0.25 |
| Rmnd5a | -0.12 | 0.27 | 0.29 | -0.14 |
| Sox4 | -0.08 | -0.31 | 0.21 | 0.32 |
| Spint2 | -3.64 | -2.84 | 1.80 | 0.16 |
| Ssbp2 | -0.26 | 0.64 | 0.48 | -0.44 |
| Stat3 | 0.53 | 0.31 | -0.03 | -0.06 |
| Tnf | -0.25 | -1.00 | -0.31 | 0.37 |
| Th2 TC | Ly6Chi:Ly6Clo | | Cbs-/-:CT | |
| --- | --- | --- | --- | --- |
| Gene name | CT | Cbs-/- | Ly6Chi | Ly6Clo |
| Arg1 | -1.67 | -1.08 | 0.12 | 0.09 |
| Ccdc184 | -0.56 | 0.18 | 0.23 | -0.05 |
| Ccl17 | 0.22 | -4.16 | -1.13 | 0.08 |
| Ccr3 | 0.84 | -0.34 | -1.22 | 0.16 |
| Ccr4 | -0.76 | -1.30 | 0.22 | 0.20 |
| Cd3e | 0.52 | -0.24 | 0.86 | 1.35 |
| Cish | -0.62 | -1.52 | 0.07 | 0.92 |
| Ctla2a | 0.01 | 0.06 | -0.40 | -0.48 |
| Furin | 1.55 | 1.01 | -0.27 | 0.17 |
| Gadd45b | 0.18 | -0.90 | -0.37 | 0.56 |
| Gata3 | -2.24 | -1.80 | 0.14 | -0.38 |
| Il1rl1 | -0.25 | -1.26 | -0.17 | 0.75 |
| Junb | 1.00 | 1.09 | -0.35 | -0.10 |
| Maf | 2.99 | 2.26 | 0.19 | 0.83 |
| Nfatc1 | -0.65 | -0.56 | 0.23 | 0.20 |
| Nfatc2ip | -0.26 | 0.03 | 0.01 | -0.26 |
| Ptgdr2 | 0.94 | -3.29 | -0.95 | 0.49 |
| Rora | 0.24 | 0.80 | 0.54 | -0.02 |
| Stat5a | 1.07 | 0.44 | -0.14 | 0.26 |
| Stat6 | 0.88 | 0.63 | 0.27 | 0.23 |
| Zbtb32 | -3.80 | -4.64 | 0.94 | 0.07 |

## Slide 15
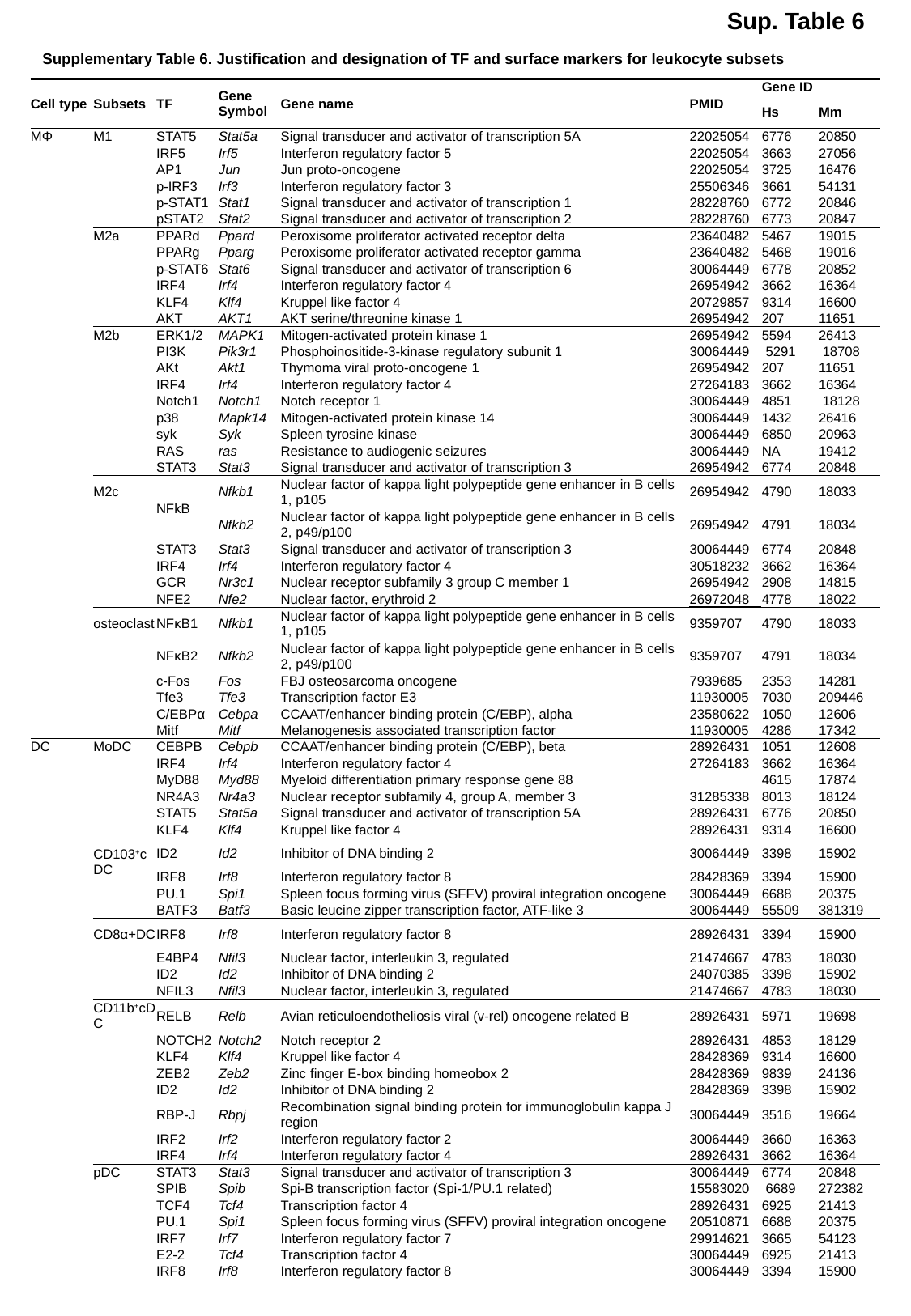

Sup. Table 6
Supplementary Table 6. Justification and designation of TF and surface markers for leukocyte subsets
| Cell type | Subsets | TF | Gene Symbol | Gene name | PMID | Gene ID | |
| --- | --- | --- | --- | --- | --- | --- | --- |
| | | | | | | Hs | Mm |
| MΦ | M1 | STAT5 | Stat5a | Signal transducer and activator of transcription 5A | 22025054 | 6776 | 20850 |
| | | IRF5 | Irf5 | Interferon regulatory factor 5 | 22025054 | 3663 | 27056 |
| | | AP1 | Jun | Jun proto-oncogene | 22025054 | 3725 | 16476 |
| | | p-IRF3 | Irf3 | Interferon regulatory factor 3 | 25506346 | 3661 | 54131 |
| | | p-STAT1 | Stat1 | Signal transducer and activator of transcription 1 | 28228760 | 6772 | 20846 |
| | | pSTAT2 | Stat2 | Signal transducer and activator of transcription 2 | 28228760 | 6773 | 20847 |
| | M2a | PPARd | Ppard | Peroxisome proliferator activated receptor delta | 23640482 | 5467 | 19015 |
| | | PPARg | Pparg | Peroxisome proliferator activated receptor gamma | 23640482 | 5468 | 19016 |
| | | p-STAT6 | Stat6 | Signal transducer and activator of transcription 6 | 30064449 | 6778 | 20852 |
| | | IRF4 | Irf4 | Interferon regulatory factor 4 | 26954942 | 3662 | 16364 |
| | | KLF4 | Klf4 | Kruppel like factor 4 | 20729857 | 9314 | 16600 |
| | | AKT | AKT1 | AKT serine/threonine kinase 1 | 26954942 | 207 | 11651 |
| | M2b | ERK1/2 | MAPK1 | Mitogen-activated protein kinase 1 | 26954942 | 5594 | 26413 |
| | | PI3K | Pik3r1 | Phosphoinositide-3-kinase regulatory subunit 1 | 30064449 | 5291 | 18708 |
| | | AKt | Akt1 | Thymoma viral proto-oncogene 1 | 26954942 | 207 | 11651 |
| | | IRF4 | Irf4 | Interferon regulatory factor 4 | 27264183 | 3662 | 16364 |
| | | Notch1 | Notch1 | Notch receptor 1 | 30064449 | 4851 | 18128 |
| | | p38 | Mapk14 | Mitogen-activated protein kinase 14 | 30064449 | 1432 | 26416 |
| | | syk | Syk | Spleen tyrosine kinase | 30064449 | 6850 | 20963 |
| | | RAS | ras | Resistance to audiogenic seizures | 30064449 | NA | 19412 |
| | | STAT3 | Stat3 | Signal transducer and activator of transcription 3 | 26954942 | 6774 | 20848 |
| | M2c | NFkB | Nfkb1 | Nuclear factor of kappa light polypeptide gene enhancer in B cells 1, p105 | 26954942 | 4790 | 18033 |
| | | | Nfkb2 | Nuclear factor of kappa light polypeptide gene enhancer in B cells 2, p49/p100 | 26954942 | 4791 | 18034 |
| | | STAT3 | Stat3 | Signal transducer and activator of transcription 3 | 30064449 | 6774 | 20848 |
| | | IRF4 | Irf4 | Interferon regulatory factor 4 | 30518232 | 3662 | 16364 |
| | | GCR | Nr3c1 | Nuclear receptor subfamily 3 group C member 1 | 26954942 | 2908 | 14815 |
| | | NFE2 | Nfe2 | Nuclear factor, erythroid 2 | 26972048 | 4778 | 18022 |
| | osteoclast | NFκB1 | Nfkb1 | Nuclear factor of kappa light polypeptide gene enhancer in B cells 1, p105 | 9359707 | 4790 | 18033 |
| | | NFκB2 | Nfkb2 | Nuclear factor of kappa light polypeptide gene enhancer in B cells 2, p49/p100 | 9359707 | 4791 | 18034 |
| | | c-Fos | Fos | FBJ osteosarcoma oncogene | 7939685 | 2353 | 14281 |
| | | Tfe3 | Tfe3 | Transcription factor E3 | 11930005 | 7030 | 209446 |
| | | C/EBPα | Cebpa | CCAAT/enhancer binding protein (C/EBP), alpha | 23580622 | 1050 | 12606 |
| | | Mitf | Mitf | Melanogenesis associated transcription factor | 11930005 | 4286 | 17342 |
| DC | MoDC | CEBPB | Cebpb | CCAAT/enhancer binding protein (C/EBP), beta | 28926431 | 1051 | 12608 |
| | | IRF4 | Irf4 | Interferon regulatory factor 4 | 27264183 | 3662 | 16364 |
| | | MyD88 | Myd88 | Myeloid differentiation primary response gene 88 | | 4615 | 17874 |
| | | NR4A3 | Nr4a3 | Nuclear receptor subfamily 4, group A, member 3 | 31285338 | 8013 | 18124 |
| | | STAT5 | Stat5a | Signal transducer and activator of transcription 5A | 28926431 | 6776 | 20850 |
| | | KLF4 | Klf4 | Kruppel like factor 4 | 28926431 | 9314 | 16600 |
| | CD103+cDC | ID2 | Id2 | Inhibitor of DNA binding 2 | 30064449 | 3398 | 15902 |
| | | IRF8 | Irf8 | Interferon regulatory factor 8 | 28428369 | 3394 | 15900 |
| | | PU.1 | Spi1 | Spleen focus forming virus (SFFV) proviral integration oncogene | 30064449 | 6688 | 20375 |
| | | BATF3 | Batf3 | Basic leucine zipper transcription factor, ATF-like 3 | 30064449 | 55509 | 381319 |
| | CD8α+DC | IRF8 | Irf8 | Interferon regulatory factor 8 | 28926431 | 3394 | 15900 |
| | | E4BP4 | Nfil3 | Nuclear factor, interleukin 3, regulated | 21474667 | 4783 | 18030 |
| | | ID2 | Id2 | Inhibitor of DNA binding 2 | 24070385 | 3398 | 15902 |
| | | NFIL3 | Nfil3 | Nuclear factor, interleukin 3, regulated | 21474667 | 4783 | 18030 |
| | CD11b+cDC | RELB | Relb | Avian reticuloendotheliosis viral (v-rel) oncogene related B | 28926431 | 5971 | 19698 |
| | | NOTCH2 | Notch2 | Notch receptor 2 | 28926431 | 4853 | 18129 |
| | | KLF4 | Klf4 | Kruppel like factor 4 | 28428369 | 9314 | 16600 |
| | | ZEB2 | Zeb2 | Zinc finger E-box binding homeobox 2 | 28428369 | 9839 | 24136 |
| | | ID2 | Id2 | Inhibitor of DNA binding 2 | 28428369 | 3398 | 15902 |
| | | RBP-J | Rbpj | Recombination signal binding protein for immunoglobulin kappa J region | 30064449 | 3516 | 19664 |
| | | IRF2 | Irf2 | Interferon regulatory factor 2 | 30064449 | 3660 | 16363 |
| | | IRF4 | Irf4 | Interferon regulatory factor 4 | 28926431 | 3662 | 16364 |
| | pDC | STAT3 | Stat3 | Signal transducer and activator of transcription 3 | 30064449 | 6774 | 20848 |
| | | SPIB | Spib | Spi-B transcription factor (Spi-1/PU.1 related) | 15583020 | 6689 | 272382 |
| | | TCF4 | Tcf4 | Transcription factor 4 | 28926431 | 6925 | 21413 |
| | | PU.1 | Spi1 | Spleen focus forming virus (SFFV) proviral integration oncogene | 20510871 | 6688 | 20375 |
| | | IRF7 | Irf7 | Interferon regulatory factor 7 | 29914621 | 3665 | 54123 |
| | | E2-2 | Tcf4 | Transcription factor 4 | 30064449 | 6925 | 21413 |
| | | IRF8 | Irf8 | Interferon regulatory factor 8 | 30064449 | 3394 | 15900 |

## Slide 16
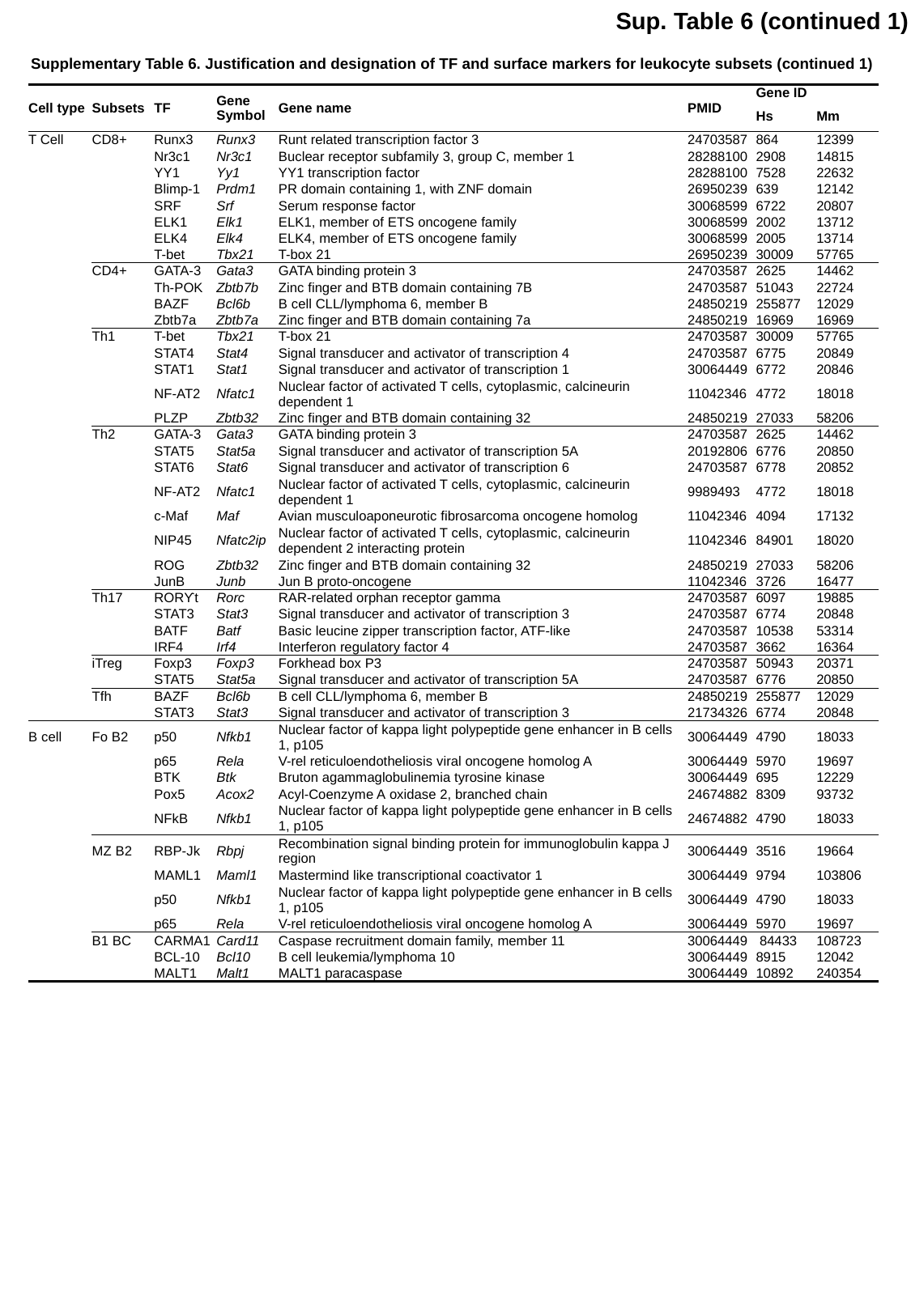

Sup. Table 6 (continued 1)
Supplementary Table 6. Justification and designation of TF and surface markers for leukocyte subsets (continued 1)
| Cell type | Subsets | TF | Gene Symbol | Gene name | PMID | Gene ID | |
| --- | --- | --- | --- | --- | --- | --- | --- |
| | | | | | | Hs | Mm |
| T Cell | CD8+ | Runx3 | Runx3 | Runt related transcription factor 3 | 24703587 | 864 | 12399 |
| | | Nr3c1 | Nr3c1 | Buclear receptor subfamily 3, group C, member 1 | 28288100 | 2908 | 14815 |
| | | YY1 | Yy1 | YY1 transcription factor | 28288100 | 7528 | 22632 |
| | | Blimp-1 | Prdm1 | PR domain containing 1, with ZNF domain | 26950239 | 639 | 12142 |
| | | SRF | Srf | Serum response factor | 30068599 | 6722 | 20807 |
| | | ELK1 | Elk1 | ELK1, member of ETS oncogene family | 30068599 | 2002 | 13712 |
| | | ELK4 | Elk4 | ELK4, member of ETS oncogene family | 30068599 | 2005 | 13714 |
| | | T-bet | Tbx21 | T-box 21 | 26950239 | 30009 | 57765 |
| | CD4+ | GATA-3 | Gata3 | GATA binding protein 3 | 24703587 | 2625 | 14462 |
| | | Th-POK | Zbtb7b | Zinc finger and BTB domain containing 7B | 24703587 | 51043 | 22724 |
| | | BAZF | Bcl6b | B cell CLL/lymphoma 6, member B | 24850219 | 255877 | 12029 |
| | | Zbtb7a | Zbtb7a | Zinc finger and BTB domain containing 7a | 24850219 | 16969 | 16969 |
| | Th1 | T-bet | Tbx21 | T-box 21 | 24703587 | 30009 | 57765 |
| | | STAT4 | Stat4 | Signal transducer and activator of transcription 4 | 24703587 | 6775 | 20849 |
| | | STAT1 | Stat1 | Signal transducer and activator of transcription 1 | 30064449 | 6772 | 20846 |
| | | NF-AT2 | Nfatc1 | Nuclear factor of activated T cells, cytoplasmic, calcineurin dependent 1 | 11042346 | 4772 | 18018 |
| | | PLZP | Zbtb32 | Zinc finger and BTB domain containing 32 | 24850219 | 27033 | 58206 |
| | Th2 | GATA-3 | Gata3 | GATA binding protein 3 | 24703587 | 2625 | 14462 |
| | | STAT5 | Stat5a | Signal transducer and activator of transcription 5A | 20192806 | 6776 | 20850 |
| | | STAT6 | Stat6 | Signal transducer and activator of transcription 6 | 24703587 | 6778 | 20852 |
| | | NF-AT2 | Nfatc1 | Nuclear factor of activated T cells, cytoplasmic, calcineurin dependent 1 | 9989493 | 4772 | 18018 |
| | | c-Maf | Maf | Avian musculoaponeurotic fibrosarcoma oncogene homolog | 11042346 | 4094 | 17132 |
| | | NIP45 | Nfatc2ip | Nuclear factor of activated T cells, cytoplasmic, calcineurin dependent 2 interacting protein | 11042346 | 84901 | 18020 |
| | | ROG | Zbtb32 | Zinc finger and BTB domain containing 32 | 24850219 | 27033 | 58206 |
| | | JunB | Junb | Jun B proto-oncogene | 11042346 | 3726 | 16477 |
| | Th17 | RORϒt | Rorc | RAR-related orphan receptor gamma | 24703587 | 6097 | 19885 |
| | | STAT3 | Stat3 | Signal transducer and activator of transcription 3 | 24703587 | 6774 | 20848 |
| | | BATF | Batf | Basic leucine zipper transcription factor, ATF-like | 24703587 | 10538 | 53314 |
| | | IRF4 | Irf4 | Interferon regulatory factor 4 | 24703587 | 3662 | 16364 |
| | iTreg | Foxp3 | Foxp3 | Forkhead box P3 | 24703587 | 50943 | 20371 |
| | | STAT5 | Stat5a | Signal transducer and activator of transcription 5A | 24703587 | 6776 | 20850 |
| | Tfh | BAZF | Bcl6b | B cell CLL/lymphoma 6, member B | 24850219 | 255877 | 12029 |
| | | STAT3 | Stat3 | Signal transducer and activator of transcription 3 | 21734326 | 6774 | 20848 |
| B cell | Fo B2 | p50 | Nfkb1 | Nuclear factor of kappa light polypeptide gene enhancer in B cells 1, p105 | 30064449 | 4790 | 18033 |
| | | p65 | Rela | V-rel reticuloendotheliosis viral oncogene homolog A | 30064449 | 5970 | 19697 |
| | | BTK | Btk | Bruton agammaglobulinemia tyrosine kinase | 30064449 | 695 | 12229 |
| | | Pox5 | Acox2 | Acyl-Coenzyme A oxidase 2, branched chain | 24674882 | 8309 | 93732 |
| | | NFkB | Nfkb1 | Nuclear factor of kappa light polypeptide gene enhancer in B cells 1, p105 | 24674882 | 4790 | 18033 |
| | MZ B2 | RBP-Jk | Rbpj | Recombination signal binding protein for immunoglobulin kappa J region | 30064449 | 3516 | 19664 |
| | | MAML1 | Maml1 | Mastermind like transcriptional coactivator 1 | 30064449 | 9794 | 103806 |
| | | p50 | Nfkb1 | Nuclear factor of kappa light polypeptide gene enhancer in B cells 1, p105 | 30064449 | 4790 | 18033 |
| | | p65 | Rela | V-rel reticuloendotheliosis viral oncogene homolog A | 30064449 | 5970 | 19697 |
| | B1 BC | CARMA1 | Card11 | Caspase recruitment domain family, member 11 | 30064449 | 84433 | 108723 |
| | | BCL-10 | Bcl10 | B cell leukemia/lymphoma 10 | 30064449 | 8915 | 12042 |
| | | MALT1 | Malt1 | MALT1 paracaspase | 30064449 | 10892 | 240354 |

## Slide 17
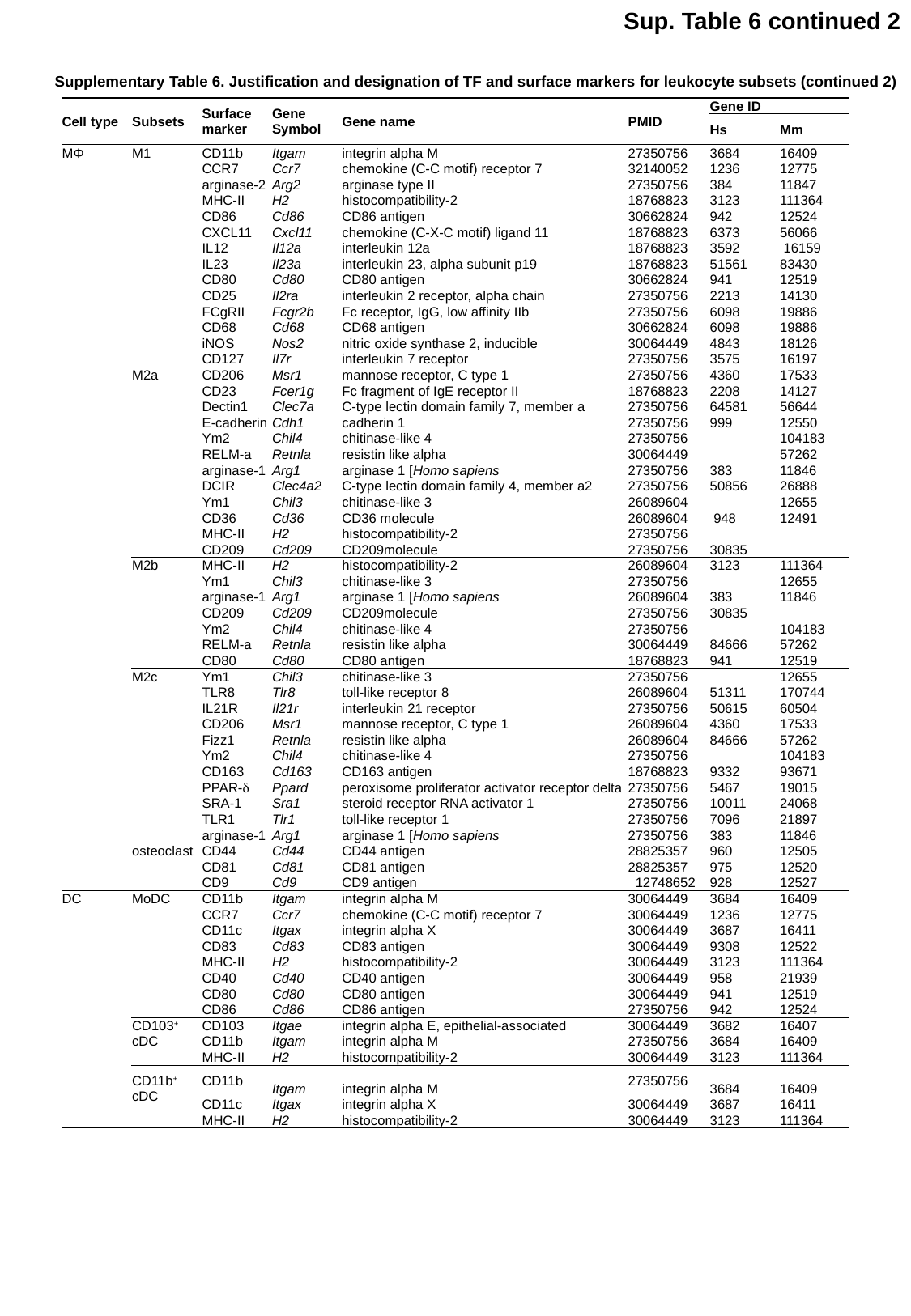

Sup. Table 6 continued 2
Supplementary Table 6. Justification and designation of TF and surface markers for leukocyte subsets (continued 2)
| Cell type | Subsets | Surface marker | Gene Symbol | Gene name | PMID | Gene ID | |
| --- | --- | --- | --- | --- | --- | --- | --- |
| | | | | | | Hs | Mm |
| MΦ | M1 | CD11b | Itgam | integrin alpha M | 27350756 | 3684 | 16409 |
| | | CCR7 | Ccr7 | chemokine (C-C motif) receptor 7 | 32140052 | 1236 | 12775 |
| | | arginase-2 | Arg2 | arginase type II | 27350756 | 384 | 11847 |
| | | MHC-II | H2 | histocompatibility-2 | 18768823 | 3123 | 111364 |
| | | CD86 | Cd86 | CD86 antigen | 30662824 | 942 | 12524 |
| | | CXCL11 | Cxcl11 | chemokine (C-X-C motif) ligand 11 | 18768823 | 6373 | 56066 |
| | | IL12 | Il12a | interleukin 12a | 18768823 | 3592 | 16159 |
| | | IL23 | Il23a | interleukin 23, alpha subunit p19 | 18768823 | 51561 | 83430 |
| | | CD80 | Cd80 | CD80 antigen | 30662824 | 941 | 12519 |
| | | CD25 | Il2ra | interleukin 2 receptor, alpha chain | 27350756 | 2213 | 14130 |
| | | FCgRII | Fcgr2b | Fc receptor, IgG, low affinity IIb | 27350756 | 6098 | 19886 |
| | | CD68 | Cd68 | CD68 antigen | 30662824 | 6098 | 19886 |
| | | iNOS | Nos2 | nitric oxide synthase 2, inducible | 30064449 | 4843 | 18126 |
| | | CD127 | Il7r | interleukin 7 receptor | 27350756 | 3575 | 16197 |
| | M2a | CD206 | Msr1 | mannose receptor, C type 1 | 27350756 | 4360 | 17533 |
| | | CD23 | Fcer1g | Fc fragment of IgE receptor II | 18768823 | 2208 | 14127 |
| | | Dectin1 | Clec7a | C-type lectin domain family 7, member a | 27350756 | 64581 | 56644 |
| | | E-cadherin | Cdh1 | cadherin 1 | 27350756 | 999 | 12550 |
| | | Ym2 | Chil4 | chitinase-like 4 | 27350756 | | 104183 |
| | | RELM-a | Retnla | resistin like alpha | 30064449 | | 57262 |
| | | arginase-1 | Arg1 | arginase 1 [Homo sapiens | 27350756 | 383 | 11846 |
| | | DCIR | Clec4a2 | C-type lectin domain family 4, member a2 | 27350756 | 50856 | 26888 |
| | | Ym1 | Chil3 | chitinase-like 3 | 26089604 | | 12655 |
| | | CD36 | Cd36 | CD36 molecule | 26089604 | 948 | 12491 |
| | | MHC-II | H2 | histocompatibility-2 | 27350756 | | |
| | | CD209 | Cd209 | CD209molecule | 27350756 | 30835 | |
| | M2b | MHC-II | H2 | histocompatibility-2 | 26089604 | 3123 | 111364 |
| | | Ym1 | Chil3 | chitinase-like 3 | 27350756 | | 12655 |
| | | arginase-1 | Arg1 | arginase 1 [Homo sapiens | 26089604 | 383 | 11846 |
| | | CD209 | Cd209 | CD209molecule | 27350756 | 30835 | |
| | | Ym2 | Chil4 | chitinase-like 4 | 27350756 | | 104183 |
| | | RELM-a | Retnla | resistin like alpha | 30064449 | 84666 | 57262 |
| | | CD80 | Cd80 | CD80 antigen | 18768823 | 941 | 12519 |
| | M2c | Ym1 | Chil3 | chitinase-like 3 | 27350756 | | 12655 |
| | | TLR8 | Tlr8 | toll-like receptor 8 | 26089604 | 51311 | 170744 |
| | | IL21R | Il21r | interleukin 21 receptor | 27350756 | 50615 | 60504 |
| | | CD206 | Msr1 | mannose receptor, C type 1 | 26089604 | 4360 | 17533 |
| | | Fizz1 | Retnla | resistin like alpha | 26089604 | 84666 | 57262 |
| | | Ym2 | Chil4 | chitinase-like 4 | 27350756 | | 104183 |
| | | CD163 | Cd163 | CD163 antigen | 18768823 | 9332 | 93671 |
| | | PPAR- | Ppard | peroxisome proliferator activator receptor delta | 27350756 | 5467 | 19015 |
| | | SRA-1 | Sra1 | steroid receptor RNA activator 1 | 27350756 | 10011 | 24068 |
| | | TLR1 | Tlr1 | toll-like receptor 1 | 27350756 | 7096 | 21897 |
| | | arginase-1 | Arg1 | arginase 1 [Homo sapiens | 27350756 | 383 | 11846 |
| | osteoclast | CD44 | Cd44 | CD44 antigen | 28825357 | 960 | 12505 |
| | | CD81 | Cd81 | CD81 antigen | 28825357 | 975 | 12520 |
| | | CD9 | Cd9 | CD9 antigen | 12748652 | 928 | 12527 |
| DC | MoDC | CD11b | Itgam | integrin alpha M | 30064449 | 3684 | 16409 |
| | | CCR7 | Ccr7 | chemokine (C-C motif) receptor 7 | 30064449 | 1236 | 12775 |
| | | CD11c | Itgax | integrin alpha X | 30064449 | 3687 | 16411 |
| | | CD83 | Cd83 | CD83 antigen | 30064449 | 9308 | 12522 |
| | | MHC-II | H2 | histocompatibility-2 | 30064449 | 3123 | 111364 |
| | | CD40 | Cd40 | CD40 antigen | 30064449 | 958 | 21939 |
| | | CD80 | Cd80 | CD80 antigen | 30064449 | 941 | 12519 |
| | | CD86 | Cd86 | CD86 antigen | 27350756 | 942 | 12524 |
| | CD103+ cDC | CD103 | Itgae | integrin alpha E, epithelial-associated | 30064449 | 3682 | 16407 |
| | | CD11b | Itgam | integrin alpha M | 27350756 | 3684 | 16409 |
| | | MHC-II | H2 | histocompatibility-2 | 30064449 | 3123 | 111364 |
| | CD11b+ cDC | CD11b | Itgam | integrin alpha M | 27350756 | 3684 | 16409 |
| | | CD11c | Itgax | integrin alpha X | 30064449 | 3687 | 16411 |
| | | MHC-II | H2 | histocompatibility-2 | 30064449 | 3123 | 111364 |

## Slide 18
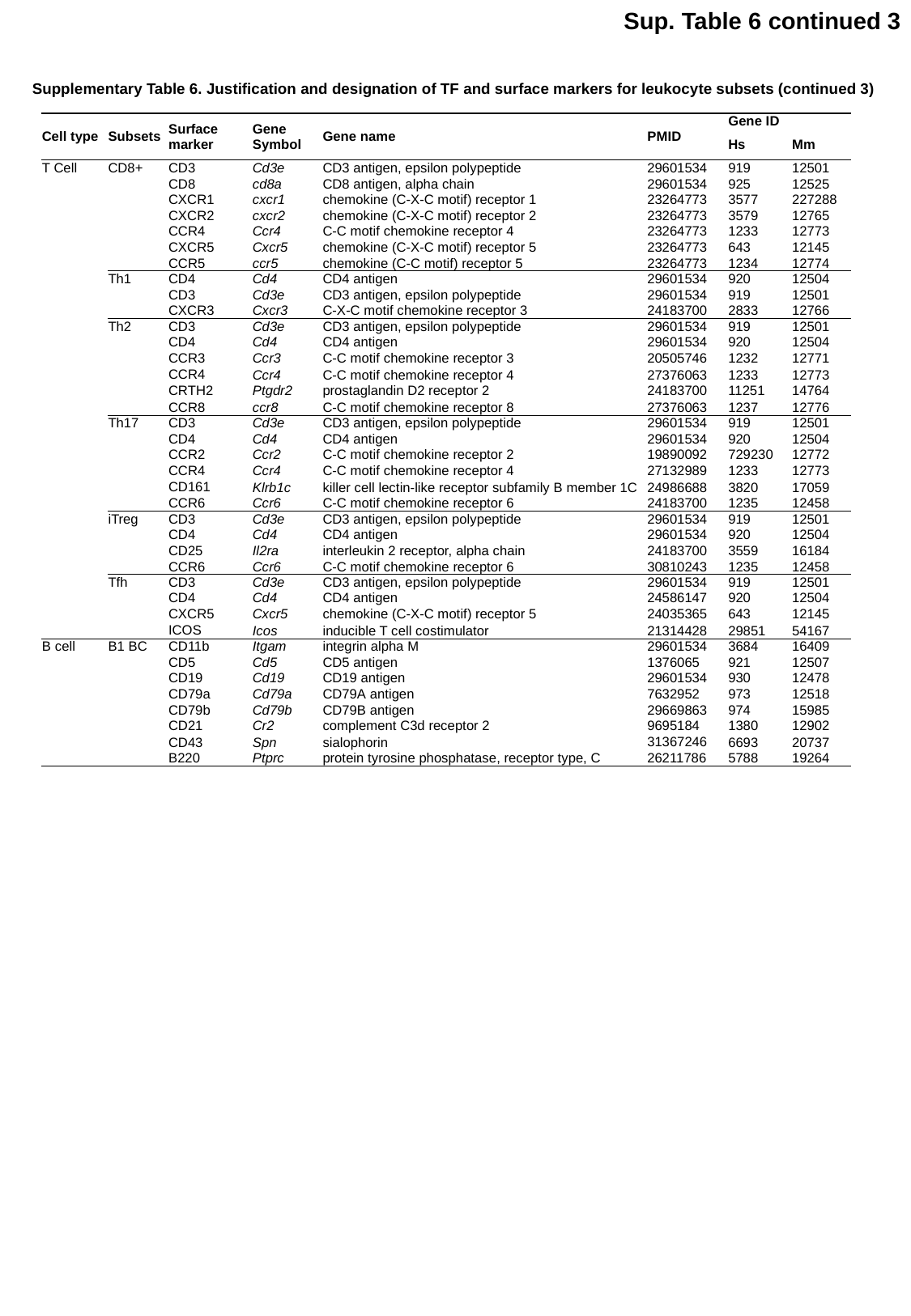

Sup. Table 6 continued 3
Supplementary Table 6. Justification and designation of TF and surface markers for leukocyte subsets (continued 3)
| Cell type | Subsets | Surface marker | Gene Symbol | Gene name | PMID | Gene ID | |
| --- | --- | --- | --- | --- | --- | --- | --- |
| | | | | | | Hs | Mm |
| T Cell | CD8+ | CD3 | Cd3e | CD3 antigen, epsilon polypeptide | 29601534 | 919 | 12501 |
| | | CD8 | cd8a | CD8 antigen, alpha chain | 29601534 | 925 | 12525 |
| | | CXCR1 | cxcr1 | chemokine (C-X-C motif) receptor 1 | 23264773 | 3577 | 227288 |
| | | CXCR2 | cxcr2 | chemokine (C-X-C motif) receptor 2 | 23264773 | 3579 | 12765 |
| | | CCR4 | Ccr4 | C-C motif chemokine receptor 4 | 23264773 | 1233 | 12773 |
| | | CXCR5 | Cxcr5 | chemokine (C-X-C motif) receptor 5 | 23264773 | 643 | 12145 |
| | | CCR5 | ccr5 | chemokine (C-C motif) receptor 5 | 23264773 | 1234 | 12774 |
| | Th1 | CD4 | Cd4 | CD4 antigen | 29601534 | 920 | 12504 |
| | | CD3 | Cd3e | CD3 antigen, epsilon polypeptide | 29601534 | 919 | 12501 |
| | | CXCR3 | Cxcr3 | C-X-C motif chemokine receptor 3 | 24183700 | 2833 | 12766 |
| | Th2 | CD3 | Cd3e | CD3 antigen, epsilon polypeptide | 29601534 | 919 | 12501 |
| | | CD4 | Cd4 | CD4 antigen | 29601534 | 920 | 12504 |
| | | CCR3 | Ccr3 | C-C motif chemokine receptor 3 | 20505746 | 1232 | 12771 |
| | | CCR4 | Ccr4 | C-C motif chemokine receptor 4 | 27376063 | 1233 | 12773 |
| | | CRTH2 | Ptgdr2 | prostaglandin D2 receptor 2 | 24183700 | 11251 | 14764 |
| | | CCR8 | ccr8 | C-C motif chemokine receptor 8 | 27376063 | 1237 | 12776 |
| | Th17 | CD3 | Cd3e | CD3 antigen, epsilon polypeptide | 29601534 | 919 | 12501 |
| | | CD4 | Cd4 | CD4 antigen | 29601534 | 920 | 12504 |
| | | CCR2 | Ccr2 | C-C motif chemokine receptor 2 | 19890092 | 729230 | 12772 |
| | | CCR4 | Ccr4 | C-C motif chemokine receptor 4 | 27132989 | 1233 | 12773 |
| | | CD161 | Klrb1c | killer cell lectin-like receptor subfamily B member 1C | 24986688 | 3820 | 17059 |
| | | CCR6 | Ccr6 | C-C motif chemokine receptor 6 | 24183700 | 1235 | 12458 |
| | iTreg | CD3 | Cd3e | CD3 antigen, epsilon polypeptide | 29601534 | 919 | 12501 |
| | | CD4 | Cd4 | CD4 antigen | 29601534 | 920 | 12504 |
| | | CD25 | Il2ra | interleukin 2 receptor, alpha chain | 24183700 | 3559 | 16184 |
| | | CCR6 | Ccr6 | C-C motif chemokine receptor 6 | 30810243 | 1235 | 12458 |
| | Tfh | CD3 | Cd3e | CD3 antigen, epsilon polypeptide | 29601534 | 919 | 12501 |
| | | CD4 | Cd4 | CD4 antigen | 24586147 | 920 | 12504 |
| | | CXCR5 | Cxcr5 | chemokine (C-X-C motif) receptor 5 | 24035365 | 643 | 12145 |
| | | ICOS | Icos | inducible T cell costimulator | 21314428 | 29851 | 54167 |
| B cell | B1 BC | CD11b | Itgam | integrin alpha M | 29601534 | 3684 | 16409 |
| | | CD5 | Cd5 | CD5 antigen | 1376065 | 921 | 12507 |
| | | CD19 | Cd19 | CD19 antigen | 29601534 | 930 | 12478 |
| | | CD79a | Cd79a | CD79A antigen | 7632952 | 973 | 12518 |
| | | CD79b | Cd79b | CD79B antigen | 29669863 | 974 | 15985 |
| | | CD21 | Cr2 | complement C3d receptor 2 | 9695184 | 1380 | 12902 |
| | | CD43 | Spn | sialophorin | 31367246 | 6693 | 20737 |
| | | B220 | Ptprc | protein tyrosine phosphatase, receptor type, C | 26211786 | 5788 | 19264 |

## Slide 19
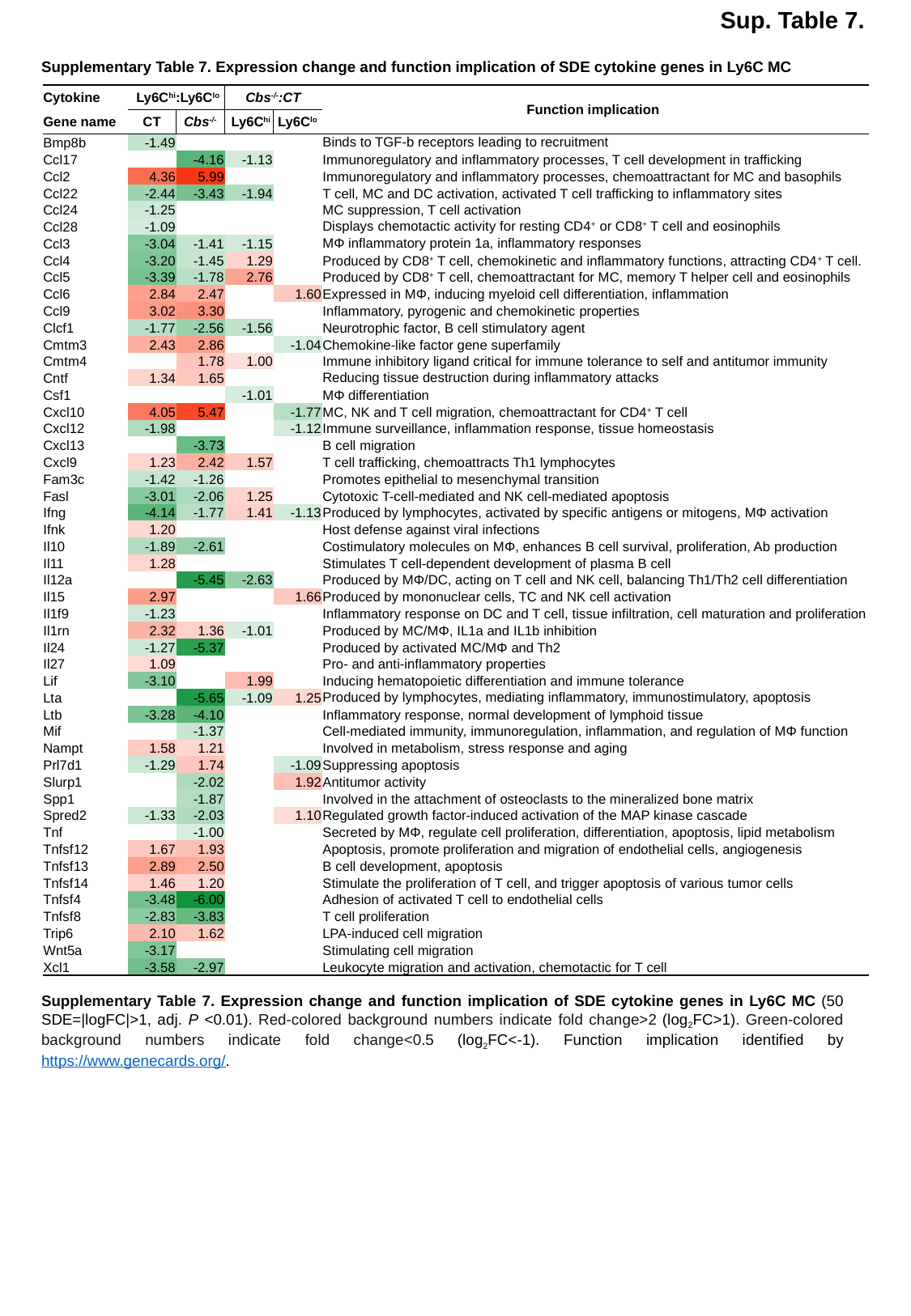

Sup. Table 7.
Supplementary Table 7. Expression change and function implication of SDE cytokine genes in Ly6C MC
| Cytokine | Ly6Chi:Ly6Clo | | Cbs-/-:CT | | Function implication |
| --- | --- | --- | --- | --- | --- |
| Gene name | CT | Cbs-/- | Ly6Chi | Ly6Clo | |
| Bmp8b | -1.49 | | | | Binds to TGF-b receptors leading to recruitment |
| Ccl17 | | -4.16 | -1.13 | | Immunoregulatory and inflammatory processes, T cell development in trafficking |
| Ccl2 | 4.36 | 5.99 | | | Immunoregulatory and inflammatory processes, chemoattractant for MC and basophils |
| Ccl22 | -2.44 | -3.43 | -1.94 | | T cell, MC and DC activation, activated T cell trafficking to inflammatory sites |
| Ccl24 | -1.25 | | | | MC suppression, T cell activation |
| Ccl28 | -1.09 | | | | Displays chemotactic activity for resting CD4+ or CD8+ T cell and eosinophils |
| Ccl3 | -3.04 | -1.41 | -1.15 | | MΦ inflammatory protein 1a, inflammatory responses |
| Ccl4 | -3.20 | -1.45 | 1.29 | | Produced by CD8+ T cell, chemokinetic and inflammatory functions, attracting CD4+ T cell. |
| Ccl5 | -3.39 | -1.78 | 2.76 | | Produced by CD8+ T cell, chemoattractant for MC, memory T helper cell and eosinophils |
| Ccl6 | 2.84 | 2.47 | | 1.60 | Expressed in MΦ, inducing myeloid cell differentiation, inflammation |
| Ccl9 | 3.02 | 3.30 | | | Inflammatory, pyrogenic and chemokinetic properties |
| Clcf1 | -1.77 | -2.56 | -1.56 | | Neurotrophic factor, B cell stimulatory agent |
| Cmtm3 | 2.43 | 2.86 | | -1.04 | Chemokine-like factor gene superfamily |
| Cmtm4 | | 1.78 | 1.00 | | Immune inhibitory ligand critical for immune tolerance to self and antitumor immunity |
| Cntf | 1.34 | 1.65 | | | Reducing tissue destruction during inflammatory attacks |
| Csf1 | | | -1.01 | | MΦ differentiation |
| Cxcl10 | 4.05 | 5.47 | | -1.77 | MC, NK and T cell migration, chemoattractant for CD4+ T cell |
| Cxcl12 | -1.98 | | | -1.12 | Immune surveillance, inflammation response, tissue homeostasis |
| Cxcl13 | | -3.73 | | | B cell migration |
| Cxcl9 | 1.23 | 2.42 | 1.57 | | T cell trafficking, chemoattracts Th1 lymphocytes |
| Fam3c | -1.42 | -1.26 | | | Promotes epithelial to mesenchymal transition |
| Fasl | -3.01 | -2.06 | 1.25 | | Cytotoxic T-cell-mediated and NK cell-mediated apoptosis |
| Ifng | -4.14 | -1.77 | 1.41 | -1.13 | Produced by lymphocytes, activated by specific antigens or mitogens, MΦ activation |
| Ifnk | 1.20 | | | | Host defense against viral infections |
| Il10 | -1.89 | -2.61 | | | Costimulatory molecules on MΦ, enhances B cell survival, proliferation, Ab production |
| Il11 | 1.28 | | | | Stimulates T cell-dependent development of plasma B cell |
| Il12a | | -5.45 | -2.63 | | Produced by MΦ/DC, acting on T cell and NK cell, balancing Th1/Th2 cell differentiation |
| Il15 | 2.97 | | | 1.66 | Produced by mononuclear cells, TC and NK cell activation |
| Il1f9 | -1.23 | | | | Inflammatory response on DC and T cell, tissue infiltration, cell maturation and proliferation |
| Il1rn | 2.32 | 1.36 | -1.01 | | Produced by MC/MΦ, IL1a and IL1b inhibition |
| Il24 | -1.27 | -5.37 | | | Produced by activated MC/MΦ and Th2 |
| Il27 | 1.09 | | | | Pro- and anti-inflammatory properties |
| Lif | -3.10 | | 1.99 | | Inducing hematopoietic differentiation and immune tolerance |
| Lta | | -5.65 | -1.09 | 1.25 | Produced by lymphocytes, mediating inflammatory, immunostimulatory, apoptosis |
| Ltb | -3.28 | -4.10 | | | Inflammatory response, normal development of lymphoid tissue |
| Mif | | -1.37 | | | Cell-mediated immunity, immunoregulation, inflammation, and regulation of MΦ function |
| Nampt | 1.58 | 1.21 | | | Involved in metabolism, stress response and aging |
| Prl7d1 | -1.29 | 1.74 | | -1.09 | Suppressing apoptosis |
| Slurp1 | | -2.02 | | 1.92 | Antitumor activity |
| Spp1 | | -1.87 | | | Involved in the attachment of osteoclasts to the mineralized bone matrix |
| Spred2 | -1.33 | -2.03 | | 1.10 | Regulated growth factor-induced activation of the MAP kinase cascade |
| Tnf | | -1.00 | | | Secreted by MΦ, regulate cell proliferation, differentiation, apoptosis, lipid metabolism |
| Tnfsf12 | 1.67 | 1.93 | | | Apoptosis, promote proliferation and migration of endothelial cells, angiogenesis |
| Tnfsf13 | 2.89 | 2.50 | | | B cell development, apoptosis |
| Tnfsf14 | 1.46 | 1.20 | | | Stimulate the proliferation of T cell, and trigger apoptosis of various tumor cells |
| Tnfsf4 | -3.48 | -6.00 | | | Adhesion of activated T cell to endothelial cells |
| Tnfsf8 | -2.83 | -3.83 | | | T cell proliferation |
| Trip6 | 2.10 | 1.62 | | | LPA-induced cell migration |
| Wnt5a | -3.17 | | | | Stimulating cell migration |
| Xcl1 | -3.58 | -2.97 | | | Leukocyte migration and activation, chemotactic for T cell |
Supplementary Table 7. Expression change and function implication of SDE cytokine genes in Ly6C MC (50 SDE=|logFC|>1, adj. P <0.01). Red-colored background numbers indicate fold change>2 (log2FC>1). Green-colored background numbers indicate fold change<0.5 (log2FC<-1). Function implication identified by https://www.genecards.org/.
