# Supplementary material for: Immunological Feature and Transcriptional Signaling of Ly6C Monocyte Subsets From Transcriptome Analysis in Control and Hyperhomocysteinemic Mice
Source: Front Immunol. 2021 Feb 25;12:632333. doi: 10.3389/fimmu.2021.632333 (PMC7947624; doi:10.3389/fimmu.2021.632333)
Supplement: Supplementary file 2 [file Table_1.docx]

**Supplemental method**

**HHcy mice —** The Tg-h*CBS* *Cbs*^-^*^/^*^-^ mice were created as described previously [[1](#_ENREF_1), [2](#_ENREF_2)]. The human CBS transgene (*Tg-hCBS*) was introduced in *Cbs*^-^*^/^*^-^ mice to rescue neonatal lethality and is under the control of a Zn-inducible metallothionein promoter [[2](#_ENREF_2)]. Mice were all born to mothers drinking ZnCl_2_ water (25 mM) to induce transgene expression [[1](#_ENREF_1), [2](#_ENREF_2)]. ZnCl_2_ was withdrawn after weaning at 1 month of age to allow the development of HHcy. Animals were fed standard rodent chow diet and sacrificed at 22 weeks for blood collection after euthanization. Mouse protocols were approved by the Temple University Institutional Animal Care and Use Committee.

**Hcy measurement —** Mouse blood was collected into 4 mM ethylenediaminetetraacetic acid (EDTA)-coated tubes. A total of 50 μL of plasma was batched and stored at -20 °C for Hcy measurement as previously described [[3](#_ENREF_3)]. In brief, total Hcy levels were tested by liquid chromatography-electrospray ionization-tandem mass spectrometry.

**Flow cytometry and cell sorting —** Mouse peripheral blood was collected into 1 ml phosphate‐buffered saline (PBS) containing 5μM EDTA in fluorescence-activated cell sorting (FACS) tube. White blood cells (WBC) were isolated by using (Ammonium-Chloride-Potassium) ACK lysing buffer (NH4Cl 0.15 M, KHCO3 10.0 mM, Na2 EDTA 0.1 mM) to lyse red blood cells. WBC from 11 mice were pooled and stained with antibodies against CD11b-Brilliant Violet 421 (myeloid cell marker, 0.25 μg/100 μL, clone M1/70), Ly6G-acticated protein C(APC)/Cy7 (granulocyte marker; 0.25 μg/100 μL, clone 1A8), Ly6C-APC (inflammatory MC marker, 0.25 μg/100 μL, clone HK1.4, BD Pharmingen, San Diego, CA), and subjected for flow cytometry cell sorting. CD11b^+^ Ly6G^-^ Ly6C^high^ and CD11b^+^ Ly6G^-^ Ly6C^low^ MC were sorted on a BD FACSAria III cell sorter. Fluorescent activated cells were analyzed offline with FlowJo software (Tree Star Inc, Ashland, OR, version 10) and compiled using Prism software (GraphPad, version 6). All populations were routinely backgated to verify gating and purity.

References:

[1] Zhang D, Jiang X, Fang P, Yan Y, Song J, Gupta S, et al. Hyperhomocysteinemia promotes inflammatory monocyte generation and accelerates atherosclerosis in transgenic cystathionine beta-synthase-deficient mice. Circulation 2009;120:1893-1902.

[2] Wang L, Jhee KH, Hua X, DiBello PM, Jacobsen DW, Kruger WD. Modulation of cystathionine beta-synthase level regulates total serum homocysteine in mice. Circulation research 2004;94:1318-1324.

[3] Ducros V, Belva-Besnet H, Casetta B, Favier A. A robust liquid chromatography tandem mass spectrometry method for total plasma homocysteine determination in clinical practice. Clinical chemistry and laboratory medicine 2006;44:987-990.
